# Spotlight on Mechanosterics: A Bulky Macrocycle Promotes Functional Group Reactivity in a [2]Rotaxane

**DOI:** 10.1021/jacs.5c08210

**Published:** 2025-07-17

**Authors:** Thomas Pickl, Claire Stark, Diego Briganti, Massimiliano Curcio, Alexander Pöthig

**Affiliations:** † Catalysis Research Center (CRC) & TUM School of Natural Sciences, Department of Chemistry, 9184 Technical University of Munich, Ernst-Otto-Fischer Str. 1, 85747 Garching, Germany; ‡ Department of Industrial Chemistry “Toso Montanari”, 9296 University of Bologna, Via Piero Gobetti 85, 40129 Bologna, Italy

## Abstract

Macrocycles typically hinder the reactivity of adjacent functional groups in mechanically interlocked molecules due to steric shielding. Herein, we report a [2]­rotaxane in which a bulky macrocycle in fact accelerates deprotection of a Fmoc-derived stopper by 36-fold compared to a non-interlocked control. We rationalize this by a preorganization of the macrocycle and the stopper, exposing its reactive site for base abstraction. This is evidenced by extensive NMR, SC-XRD, and DFT studies, revealing highly directional CH−π interactions and hydrogen bonding between the interlocked components. Our findings highlight and structurally rationalize how entanglement can instead *promote* reactivity through precise spatial control. This concept paves the way for designing molecular machines with functional, reactivity-enhancing components.

## Graphical abstract

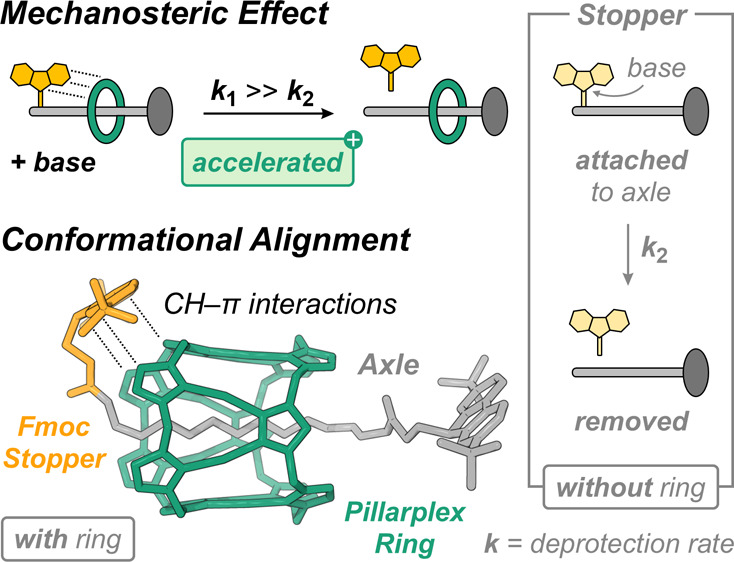

Molecular machines drive a wide range of essential biological processes, inspiring the development of artificial analogs at the nanoscale.^1^ Mechanically interlocked molecules (MIMs) have emerged as versatile synthetic platforms in this context, offering precise control over molecular motion and reactivity through mechanical bonding.^2^ A key challenge in advancing functional MIMs lies in understanding how this entanglement influences the reactivity of nearby functional groups.

One major objective is to exploit the conformational constraints imposed by mechanical bonding to create finely tuned chemical microenvironments, which underpin the function of mechanically interlocked catalysts.^3–7^ Through conformational alignment of their components, specific reaction pathways can be selectively promoted.^8^ This principle extends beyond catalysis: precise control over local reactivity within MIMs is crucial for imparting directionality to the dissipative processes that drive autonomous molecular machines.^9^

In this context, the 9-fluorenylmethoxycarbonyl (Fmoc) group is a key component, prominently used as a cleavable stopper or “bump” in molecular pumps and motors due to its well-established protection chemistry (Figure 1A).^10–13^ Previous studies have shown that its reactivity can be modulated by an adjacent macrocycle, which affects the rate of Fmoc (de)­protection through steric shielding.^10–12^ However, beyond these steric considerations, the detailed influence of mechanical bonding on Fmoc reactivity is poorly studied.

**Figure 1:**
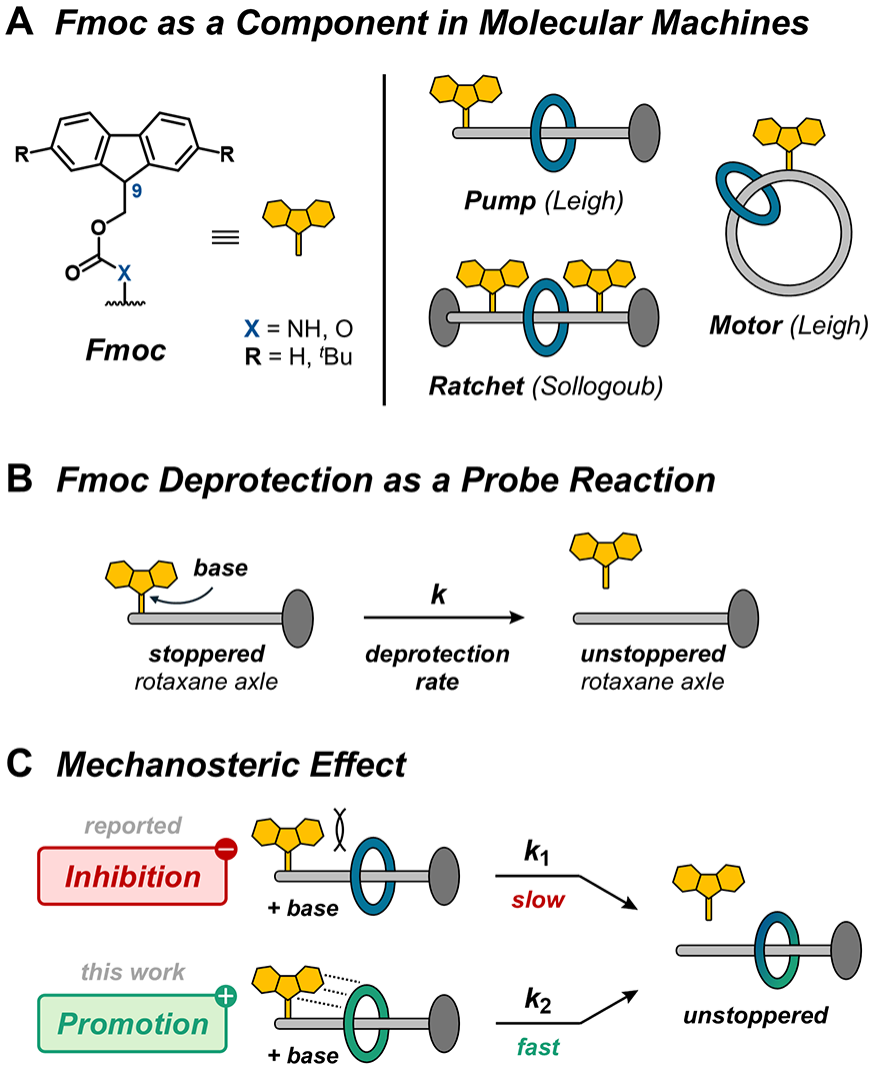
(A) The Fmoc group as a cleavable component in molecular machines. (B) Base-induced Fmoc deprotection in a non-interlocked reference to probe the influence of a macrocycle close to the Fmoc group. (C) *Mechanosteric effect*: Fmoc deprotection can be either slowed down (negative effect, *k*_1_) or accelerated (positive effect, *k*_2_) through specific interactions with the macrocycle.

More broadly, the prevailing assumption that mechanical bonds near reactive sites in MIMs inherently *inhibit* their reactivity remains largely unchallenged. To date, only two systems have been reported in which a macrocycle is employed to *promote* functional group reactivity within a MIM (in both cases supposedly via hydrogen bonding).^14–17^ While kinetic analyses confirmed a rate enhancement in both cases, the phenomenon is still poorly rationalized from a structural perspective and comprehensive studies remain elusive.

In this work, we systematically investigate the macrocycle-induced *promotion* of reactivity in a [2]­rotaxane consisting of Fmoc* stoppers and a pillarplex-based macrocycle.^18^ Our system features drastically accelerated Fmoc* deprotection, enabled by conformational preorganization between the ring and stopper through highly directional interactions. These findings redefine the role of the macrocycle in Fmoc-based MIMs, from a passive steric barrier to an active modulator of local reactivity. Acknowledging the detrimental impact of highly specific interactions between components in MIMs, we propose the term “mechanosteric effect” to describe the steric modulation of reactivity imposed by mechanical bonding, either promoting (*positive* effect) or inhibiting (*negative* effect) a reaction (Figure 1C). In principle, fine-tuning of the kinetics between positive and negative mechanosteric effects could potentially enable the design of autonomous molecular motors relying on protection/deprotection reactions.

We synthesized the [2]­rotaxane [Fmoc*-Rot]­[Ag_**8**_ **L**_**2**_ **]­(PF**_**6**_ **)**_**4**_ following a strategy previously established by our group.^19,20^ First, a pseudorotaxane was formed by stirring pillarplex **[Ag**_**8**_ **L**_**2**_ **]­(PF**_**6**_ **)**_**4**_ with 1,12-diaminododecane in acetonitrile. Subsequent protection of the terminal amines with 2,7-^*t*^Bu_2_-substituted Fmoc *N*-hydroxysuccinimide (Fmoc*-OSu) afforded the stoppered MIM in 68% isolated yield (Figure 2A). The interlocked structure was confirmed by 2D NMR experiments (SI, Section 3), and the composition of the rotaxane was supported by elemental analysis.

**Figure 2:**
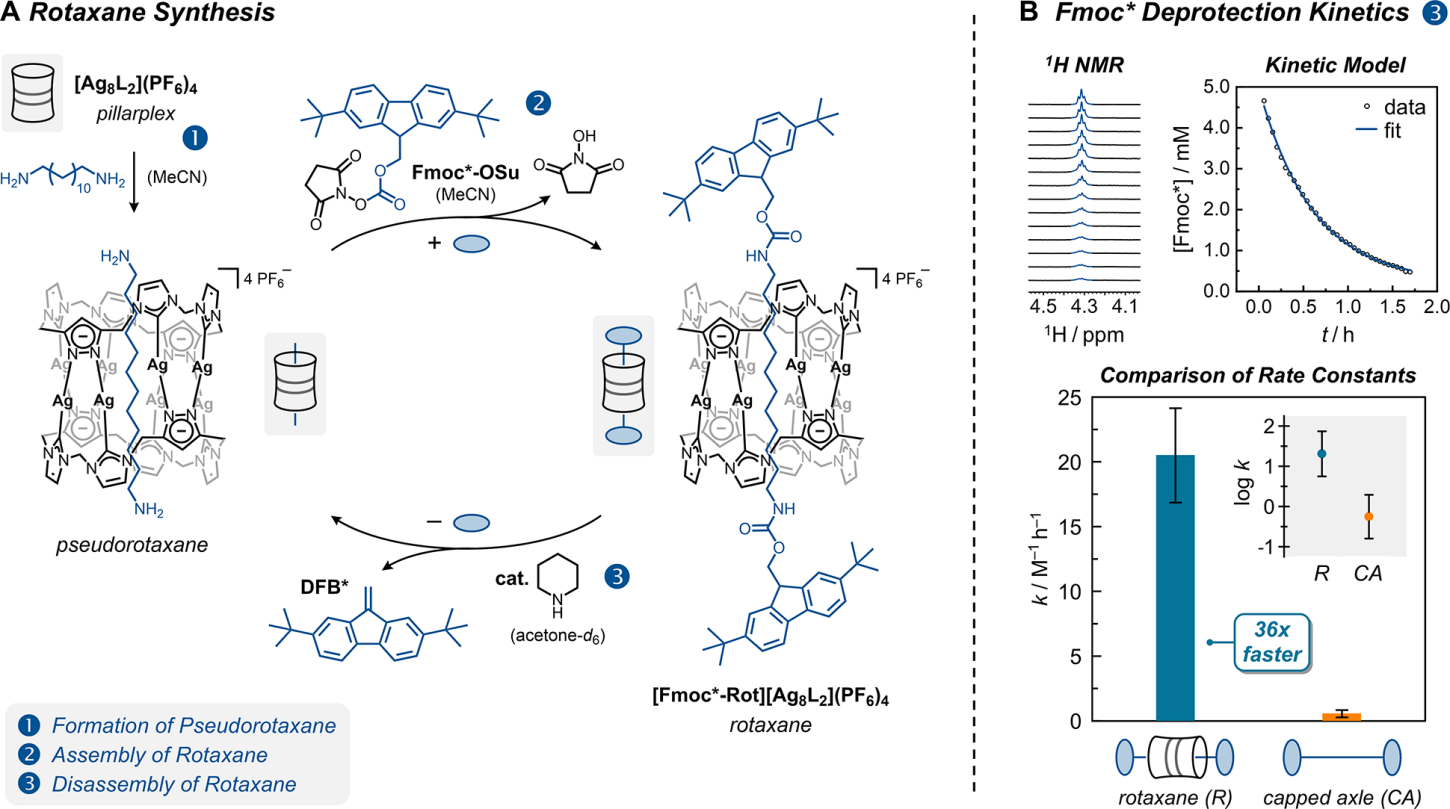
(A) 1: Synthesis of [2]­rotaxane [Fmoc*-Rot]­[Ag_**8**_ **L**_**2**_ **]­(PF**_**6**_ **)**_**4**_ via insertion of 1,12-diaminododecane into pillarplex **[Ag**_**8**_ **L**_**2**_ **]­(PF**_**6**_ **)**_**4**_, 2: Stoppering with Fmoc* *N*-hydroxysuccinimide (Fmoc*-OSu), 3: Piperidine-induced Fmoc* deprotection triggers rotaxane disassembly. (B) Kinetic analysis of Fmoc* deprotection in acetone-*d*_6_ for the [2]­rotaxane (4.8–6.0 mM) and its non-interlocked control [Fmoc*-NH-(CH_**2**_ **)**_**6**_ **]**_**2**_ (5.2–7.2 mM) in the presence of piperidine (16.3–23.4 equiv), monitored by ^1^H NMR (500 MHz, 295 K) via the decay of the C^9^–H resonance. Monoexponential fits of the data indicate pseudo-first-order kinetics in both cases and a 36-fold faster deprotection for the rotaxane.

In earlier work, we showed that lowering the pH selectively triggers interconversion of the ring component in pillarplex rotaxanes.^19^ In this study, we explored elevated pH as an orthogonal stimulus to selectively target the stoppered axle. In light of the base sensitivity of the Fmoc* group, we envisioned that exposure to piperidine (as a pH stimulus) would enable selective removal of the stopper, allowing reversible interconversion between the pseudorotaxane and the fully interlocked rotaxane. This approach offers modular switching of individual rotaxane components as an essential step toward the construction of complex molecular machines based on pillarplex scaffolds. Qualitatively, experiments in acetone-*d*_6_ confirmed that the Fmoc* stoppers in [Fmoc*-Rot]­[Ag_**8**_ **L**_**2**_ **]­(PF**_**6**_ **)**_**4**_ can be cleaved in the presence of piperidine, triggering rotaxane disassembly (Figure 2A and Figure S46). To quantitatively assess how the bulky macrocyclic ring influences Fmoc* reactivity, we also synthesized the corresponding non-interlocked analogue of the rotaxane, [Fmoc*-NH-(CH_**2**_ **)**_**6**_ **]**_**2**_, as a reference compound (SI, Section 3). The Fmoc* deprotection kinetics of both the rotaxane and the capped axle were then monitored by ^1^H NMR spectroscopy in acetone-*d*_6_.

Fmoc deprotection is typically assumed to proceed in two steps via a base-promoted E1cB mechanism.^21,22^ Deprotonation at the 9-position (C^9^–H) of the fluorene ring system triggers elimination of the carbamate group, forming the unprotected amine and dibenzofulvene (DBF) upon release of CO_2_. Owing to the chemical similarity between Fmoc and Fmoc*, we expect a comparable reaction pathway for their deprotection (Figure S1). Indeed, upon addition of piperidine to the rotaxane and non-interlocked capped axle, ^1^H NMR tracking of the methine (C^9^–H) proton signal over time afforded monoexponential decay profiles (Figure 2B and Figures S2 and S3), consistent with pseudo-first-order kinetics. This suggests that only one of the two steps in Fmoc* deprotection is rate-limiting, as reported for analogous Fmoc deprotection.^23^ Hereby, in an aprotic and moderately polar solvent such as acetone, the rate-determining step is likely to be the initial deprotonation.^23,24^

Strikingly, the Fmoc* deprotection proceeded significantly faster in the rotaxane than in the capped axle,^25^ revealing a pronounced difference in reactivity (Figure 2B and SI, Section 4). Specifically, [Fmoc*-Rot]­[Ag_**8**_ **L**_**2**_ **]­(PF**_**6**_ **)**_**4**_ exhibits a second-order rate constant *k* of 20.5 M^–1^ h^–1^ [CI_95%_: 16.9, 24.1], compared to just 0.56 M^–1^ h^–1^ [CI_95%_: 0.27, 0.85] for non-interlocked [Fmoc*-NH-(CH_**2**_ **)**_**6**_ **]**_**2**_. This corresponds to a 36-fold rate enhancement of the rotaxane, which is unprecedented in light of previous observations: so far, the presence of bulky macrocycles near an Fmoc group in MIMs has been associated with reduced reactivity due to steric shielding, corresponding to a negative mechanostereochemical influence on the reaction rate.^10,12^ In contrast, our system demonstrates that a large macrocyclic ring can actively promote Fmoc* cleavage despite its considerable steric bulk. This unexpected rate enhancement highlights that mechanical bonds can be exploited not only to constrain motion, but also to precisely modulate local reactivity by the deliberate choice of stopper–ring pairings.

To understand the origin of this positive mechanosteric effect, we investigated the spatial arrangement of the rotaxane components. A hypothesis explaining the accelerated Fmoc* deprotection in the pillarplex rotaxane was developed through analysis of its solid-state structure. Single-crystal X-ray diffraction of [Fmoc*-Rot]­[Ag_**8**_ **L**_**2**_ **]­(PF**_**6**_ **)**_**4**_ revealed the presence of different conformational isomers, reflecting a high degree of flexibility in the orientation of the Fmoc* stopper (Figure S49). Among two isolated solid-state structures, one solvatomorph features pronounced intramolecular interactions between the stopper and the pillarplex (Figure 3). In this conformation, a bulky Fmoc* group wraps around the aromatic rim of the macrocycle. As a result, the reactive C^9^–H proton of the fluorenyl moiety is exposed (Figure 3B), effectively preorganizing the system for potential base-mediated deprotonation. This “wrapped” conformation results from a combination of moderately strong CH−π interactions between the fluorenyl π system and C–H protons of the pillarplex ligand, anchoring the Fmoc* group to the pillarplex rim. Weak hydrogen bonding between a carbamate oxygen and the ligand backbone may also add stabilization (Figure 3B). Underpinning these effects are structural and electronic characteristics of pillarplexes, most notably their extended π-surface and electron-deficient rim.^26^ In solution, such a conformational alignment would likely reduce the entropic penalty of the Fmoc* deprotection, thus providing a structural rationale for the unusually fast deprotection observed in the rotaxane.

**Figure 3:**
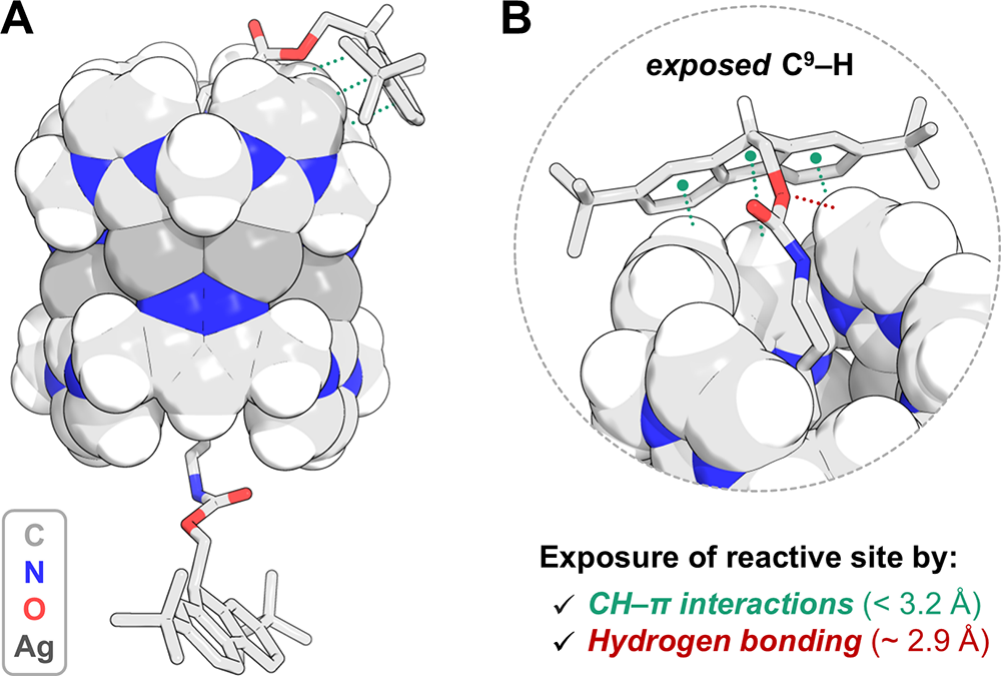
(A) SC-XRD structure of [Fmoc*-Rot]­[Ag_**8**_ **L**_**2**_ **]­(PF**_**6**_ **)**_**4**_ (solvatomorph B). (B) Perspective (partial) view of the rotaxane, highlighting the “wrapped” conformation stabilized by a combination of intramolecular CH−π interactions (green) and hydrogen bonds (red) between Fmoc* and the pillarplex rim (axle shown as capped sticks, pillarplex as spheres). Counterions, solvent molecules, and selected hydrogen atoms were omitted for clarity.

To assess whether the “wrapped” conformation observed in the solid state persists in solution, we turned to NMR spectroscopy. The ^1^H NMR spectrum of [Fmoc*-Rot]­[Ag_**8**_ **L**_**2**_ **]­(PF**_**6**_ **)**_**4**_ in acetone-*d*_6_ shows a single set of signals for the pillarplex and the Fmoc* stoppers (Figure S24 and Figure S33). Notably, significant deviations in the chemical shifts of key resonances between the Fmoc-stoppered rotaxane and parent pillarplex **[Ag**_**8**_ **L**_**2**_ **]­(PF**_**6**_ **)**_**4**_ were found (Δδ = |δ_pillarplex_ – δ_rotaxane_|).^18^ Specifically, the pyrazolate proton (H_h_, Δδ = 0.18 ppm), the adjacent *N*-heterocyclic carbene (NHC) proton (H_j_, Δδ = 0.25 ppm), and one of the methylene protons bridging both of the heterocycles (H_i_, Δδ = 0.16 ppm) exhibit substantial upfield shifts (Figure 4A). These pronounced changes are consistent with shielding from aromatic ring currents,^27^ supporting the persistence of the “wrapped” conformation in solution. In accordance with the solid-state structure, only those protons positioned directly beneath the aromatic fluorenyl ring are affected (Figure 3B), providing strong evidence for CH−π interactions in solution. Notably, all other ligand-associated signals remain virtually unchanged (Δδ ≤ 0.04 ppm). Along this line, we examined a control [2]­rotaxane, **[Amide-Rot]­[Ag**_**8**_ **L**_**2**_ **]­(PF**_**6**_ **)**_**4**_, bearing 3,5-^*t*^Bu_2_-benzamide stoppers in place of Fmoc* (SI, Section 3). The ^1^H NMR spectrum of this amide-based rotaxane shows no upfield shift with respect to the parent pillarplex, indicating an *absence* of CH−π interactions between this stopper and the pillarplex rim (Figure S45). This reinforces that the specific stopper–macrocycle pairing between the pillarplex and the Fmoc* group provides the conformational preorganization of the rotaxane components.

**Figure 4:**
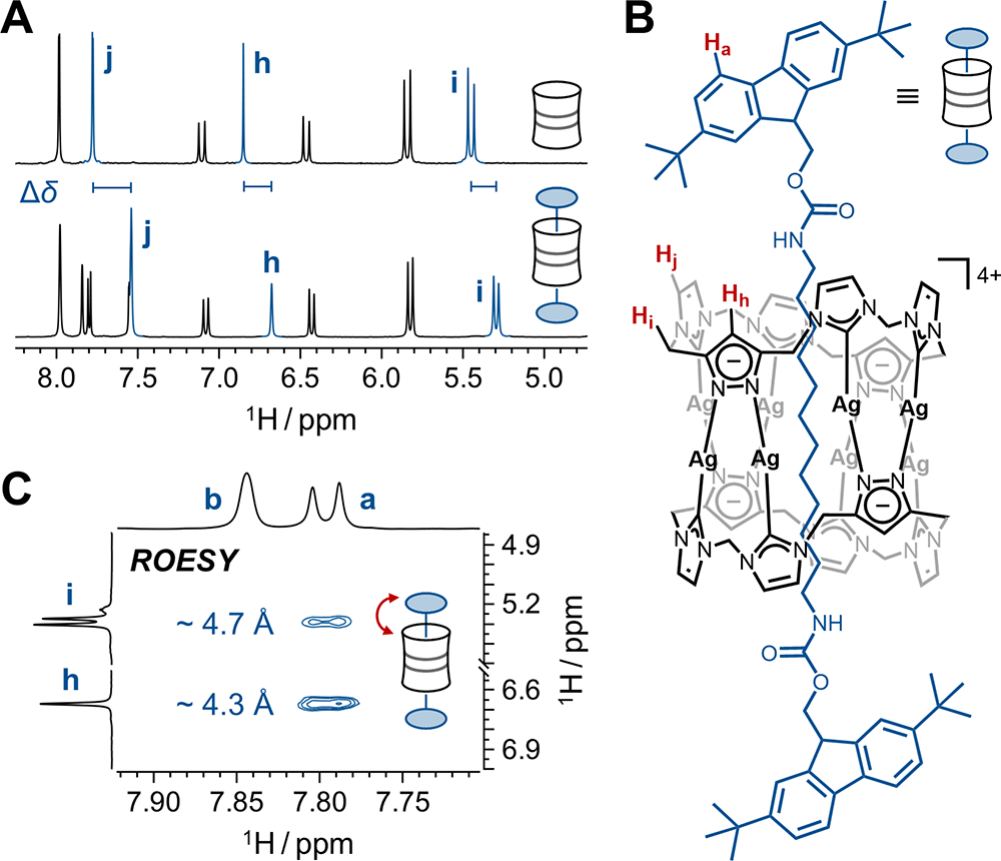
(A) Partial ^1^H NMR spectra of pillarplex **[Ag**_**8**_ **L**_**2**_ **]­(PF**_**6**_ **)**_**4**_ and rotaxane [Fmoc*-Rot]­[Ag_**8**_ **L**_**2**_ **]­(PF**_**6**_ **)**_**4**_, highlighting pillarplex-associated protons H_h_, H_i_, and H_j_ and their chemical shift differences (Δδ) in blue. (B) Schematic representation of [Fmoc*-Rot]­[Ag_**8**_ **L**_**2**_ **]­(PF**_**6**_ **)**_**4**_ with selected protons (among all symmetry-equivalent H_a_, H_h_, H_i_, and H_j_) shown in red (pillarplex = black; stoppered axle = blue). (C) Partial ^1^H,^1^H 2D ROESY spectrum of [Fmoc*-Rot]­[Ag_**8**_ **L**_**2**_ **]­(PF**_**6**_ **)**_**4**_ showing cross-peaks for H_a_···H_h_ and H_a_···H_i_ correlations. Estimated upper bounds for interproton distances are indicated in blue.

The persistence of the “wrapped” conformation of Fmoc* rotaxane [Fmoc*-Rot]­[Ag_**8**_ **L**_**2**_ **]­(PF**_**6**_ **)**_**4**_ in solution was further supported by 2D ^1^H,^1^H ROESY experiments. Cross-peaks between Fmoc*- and pillarplex-associated protons confirm spatial proximity of both components in solution. Specifically, the clearly resolved fluorenyl proton H_a_ (7.80 ppm) shows cross-relaxation with both the pyrazolate proton H_h_ (6.67 ppm) and the bridging methylene group H_i_ (5.29 ppm) discussed above (Figure 4C, Note: the expected third correlation between H_a_ and the NHC backbone proton H_j_ cannot be resolved due to resonance overlap). For a quantitative estimation of the interproton distances in the rotaxane, ROESY intensities were calibrated using reference distances derived from the solid-state structure of [Fmoc*-Rot]­[Ag_**8**_ **L**_**2**_ **]­(PF**_**6**_ **)**_**4**_ (SI, Section 5). Specifically, the fluorenyl–pyrazolate distance H_a_···H_h_ and the fluorenyl–methylene distance H_a_···H_i_ were estimated at ca. 4.7 Å and 4.3 Å, respectively (Figure 4C and Table S4). These values are fully consistent with the “wrapped” geometry observed in the crystal structure, and support that the same key CH−π interactions are predominant in solution.

DFT calculations further corroborate that the experimentally observed “wrapped” rotaxane conformer is thermodynamically preferred. Conformational sampling of a simplified host–guest model revealed that conformers lacking close CH−π or hydrogen bonding interactions were markedly destabilized compared to the “wrapped” orientation of the Fmoc* group around the pillarplex rim (SI, Section 9). These results provide an explanation for its persistence both in the solid state and in solution.

In summary, we have highlighted and structurally rationalized the *positive* mechanosteric effect, i.e. how macrocycles can actively promote, rather than inhibit, the reactivity of adjacent functional groups within MIMs. In a Fmoc*-stoppered [2]­rotaxane, [Fmoc*-Rot]­[Ag_**8**_ **L**_**2**_ **]­(PF**_**6**_ **)**_**4**_, conformational preorganization significantly accelerates base-induced deprotection by 36-fold compared to a non-interlocked control. Solution and solid-state studies, backed up by computations, collectively support the conclusion that the rate enhancement arises from a “wrapped” conformation stabilized primarily by CH−π interactions. Control experiments with an amide-stoppered rotaxane confirm that these CH−π interactions are intrinsic to the Fmoc*–pillarplex pairing. Beyond identifying a new functional role for pillarplex-based macrocycles in MIMs, this work showcases the potential of fine-tuning the mechanosteric effect as a design principle for autonomous molecular machines.

## Supplementary Material







## Data Availability

The raw NMR data for the kinetic studies in this publication are publicly available on Zenodo at 10.5281/zenodo.15692776.

## Notes

Synthetic procedures, characterization data such as NMR spectra, HR-MS spectra, crystal structure data (SC-XRD), kinetic analysis, computational details, additional figures, and discussion (PDF)
Atomic coordinates of sampled conformers obtained by using the GOAT algorithm at the GFN2-xTB level of theory (XYZ)
Atomic coordinates of the reoptimized subset of conformers at the r^2^SCAN-3c level of theory (XYZ)
