# Supplementary material for: Spotlight on Mechanosterics: A Bulky Macrocycle Promotes Functional Group Reactivity in a [2]Rotaxane
Source: J Am Chem Soc. 2025 Jul 17;147(31):27192–6. doi: 10.1021/jacs.5c08210 (PMC12333370; doi:10.1021/jacs.5c08210)
Supplement: Supplementary file 1 [file ja5c08210_si_001.pdf]

Supporting Information

for

Spotlight on Mechanosterics: A Bulky Macrocycle Promotes Functional Group Reactivity in a [2]Rotaxane

Thomas Pickl,[a] Claire Stark,[a] Diego Briganti,[a,b] Massimiliano Curcio,[b] and Alexander Pöthig,[a]*

a) Technical University of Munich, TUM School of Natural Sciences and Catalysis Research Center (CRC), Ernst-Otto-Fischer Str. 1, 85748 Garching (Germany).

b) Department of Industrial Chemistry "Toso Montanari", University of Bologna, Via Piero Gobetti 85, 40129 Bologna (Italy).

Correspondence to: alexander.poethig@tum.de

TABLE OF CONTENTS

1. General Information 1
2. Analytical Methods 1
3. Experimental Procedures 3
4. Kinetic Studies 10
5. Interproton Distance Determination by NMR 17
6. NMR Spectra 19
7. HR-MS Spectra 41
8. Crystallographic Details 42
9. Computational Details 45
10. References 49

1. General Information

All syntheses were performed under an Argon atmosphere (4.6, *Westfalen*) using standard Schlenk techniques or within an *MBraun* Labmaster Pro SP glovebox, unless stated otherwise. HPLC-grade acetonitrile (MeCN) and diethyl ether (Et_2O) were purchased from *Sigma Aldrich*, dried using an *MBraun* solvent purification system and stored over 3 Å molecular sieves. Other solvents were purchased from *Sigma Aldrich* in HPLC grade and used as received without further purification. MeCN-d_3 (99.8 atom% D) and benzene-d_6 (99.6 atom% D) were obtained from *Eurisotop* and *Sigma Aldrich*, respectively, and dried over activated 3 Å molecular sieves prior to use. Dry acetone-d_6 (99.5 atom% D) was purchased from Sigma Aldrich and used without further purification. Macrocycle **$H_6L(PF_6)_4$**,[1] pillarplex **$[Ag_8L_2](PF_6)_4$**,[2] 2,7-di-*tert*-butyl-9-fluorenylmethanol (**Fmoc*-OH**),[3] and 3,5-di-*tert*-butylbenzoic anhydride[4] were synthetized according to previously reported procedures. 1,12-diaminododecane was purchased from *Sigma Aldrich* and purified by sublimation (85°C, 0.05 mbar) prior to use. 2,7-Di-*tert*-butylfluorene (*TCI*), N,N-diisopropylethylamine (*TCI*), N,N′-disuccinimidyl carbonate (*Acros Organics*), pyridine (*Merck*) and KOH (85 wt%, *Grüssing*) *were purchased* from commercial suppliers and used without further purification.

2. Analytical Methods

NMR spectra were recorded on a *Bruker* AV-400, AVHD-400, AVHD-500 or AV-III-500 (equipped with a QNP cryo probe) NMR spectrometer in MeCN-d_3 or acetone-d_6. Chemical shifts δ are given in parts per million (ppm) and scalar coupling constants ^nJ are given in Hertz (Hz). ^{1}H and ^{13}C NMR chemical shifts are reported relative to the residual solvent signal of MeCN-d_3 (^{1}H: δ = 1.94 ppm, ^{13}C: δ = 1.32 ppm), acetone-d_6 (^{1}H: δ = 2.05 ppm, ^{13}C: δ = 29.84 ppm), or benzene-d_6 (^{1}H: δ = 7.16 ppm, ^{13}C: δ = 128.06 ppm), with respect to tetramethylsilane. ^{19}F NMR and ^{31}P shifts are reported relative to CCl_3F and H_3PO_4, respectively, as implemented in *MestReNova*. Diastereotopic protons are marked with an apostrophe (*e.g.* H_a and $H_{a'}$) and were assigned *via* ^{1}H,^{1}H ROESY experiments. Hydrogen-bound ^{13}C nuclei were assigned by phase-sensitive ^{1}H,^{13}C HSQC experiments with CH and CH_3 signals shown in blue and CH_2 signals colored red. ^{1}H,^{13}C HMBC spectroscopy allowed for the assignment of the remaining quaternary carbons. The following abbreviations are used for reporting the multiplicity of NMR resonances: s = singlet, bs = broad singlet, d = doublet, t = triplet, q = quartet, hept = heptet, and m = multiplet.

HR-HESI-MS (ESI$^+$) spectra were recorded on a *Thermo Fisher* Exactive Plus Orbitrap mass spectrometer equipped with a *Thermo Fisher* HESI source. Samples were prepared as ~ 100 µg/mL solutions in acetonitrile, syringe filtered and injected with an ionisation voltage of 3.80 kV. Peaks were fitted with Gaussian functions and compared to isotopic fine structures calculated by the web-interface of the R package *enviPat*.[5]

Elemental analyses were performed by the Elemental Analysis Laboratory of the Catalysis Research Center at the Technical University of Munich on a *Eurovector* EA3000 CHNS combustion analyzer. Roughly 1 mg of sample was weighed on a *Sartorius* CP2P microbalance (± 1 µg resolution), then dynamically spontaneously combusted in a tin boat at 1800 °C, separated by gas chromatography, and detected with a thermal conductivity detector. For calibration, sulfanilamide and 2,5-bis-(5-*tert*-butyl-2-benzoxazol-2-yl)-thiophenone (BBOT) were used as (NIST-certified) reference materials. The presented values are single or double determinations.

3. Experimental Procedures

Fmoc*-OSu

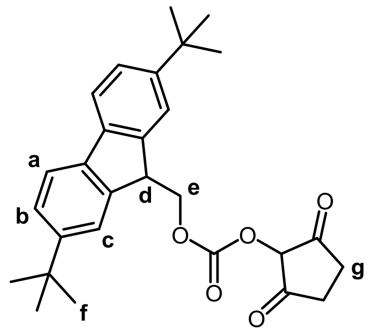

Under an inert atmosphere, **Fmoc*-OH** (283 mg, 0.92 mmol, 1.0 eq.) was weighed into a preheated Schlenk tube and dissolved in anhydrous acetonitrile (4 mL) under stirring. To the resulting colorless (occasionally faint yellow) solution, *N*,*N'*-disuccinimidyl carbonate (401 mg, 1.38 mmol, 1.5 eq.; 88 wt% pure, containing *N*-hydroxysuccinimide as an impurity), was added, resulting in a faint yellow solution. Pyridine (222 µL, 2.75 mmol, 3.0 eq.) was then added, and the mixture was stirred under an inert atmosphere for 15 h (TLC typically indicated complete consumption of **Fmoc*-OH** already after approx. 8 h). Upon completion of the reaction, all volatiles were removed under reduced pressure, and the resulting off-white solid was redissolved in a minimum amount of acetonitrile. After dilution with diethyl ether (100 mL), the solution was washed with 0.1 M HCl (2 × 45 mL). The organic phase was concentrated to dryness, redissolved in acetonitrile (20 mL), and washed with hexane (3 × 30 mL, top layer: acetonitrile). The combined acetonitrile phases were concentrated to dryness and dried *in vacuo* for several days, affording **Fmoc*-OSu** as a white solid (348 mg, 84%). The product can be stored for extended periods at 4 °C under ambient atmosphere. The analytical data are in agreement with the literature.[3]

NOTE: Small amounts (≤ 0.2 eq.) of *N*-hydroxysuccinimide (HOSu) may occasionally remain in the product. However, this impurity does not interfere with subsequent amine protections involving **Fmoc*-OSu**, as HOSu is also a byproduct of these reactions.

^{1}H NMR (400.1 MHz, MeCN-d_3, 298 K): δ [ppm] = 7.72 (d, ^3J = 8.0 Hz, 2H, H_a), 7.70 (*virt.* t, $^4J \approx {}^4J$ = 1.9 Hz, 2H, H_c), 7.49 (dd, ^3J = 8.0 Hz, ^4J = 1.9 Hz, 2H, H_b), 4.82 (d, ^3J = 5.6 Hz, 2H, H_e), 4.31 (t, ^3J = 5.6 Hz, 1H, H_d), 2.67 (s, 4H, H_g), 1.37 (s, 18H, H_f).

EA (%) for **Fmoc*-OSu** · 0.05 HOSu · 0.15 Et_2O: calc. C 71.59, H 7.08, N 3.15; found C 71.45, H 7.00, N 3.15. Even after prolonged times of drying *in vacuo*, trace amounts of Et_2O could not be removed and remained in the sample (*cf.* Figure S6).

TLC: R_f = 0.21 (hexane/EtOAc = 3:1) [$KMnO_4$].

[Fmoc*-NH-(CH$_2$)$_6$]$_2$

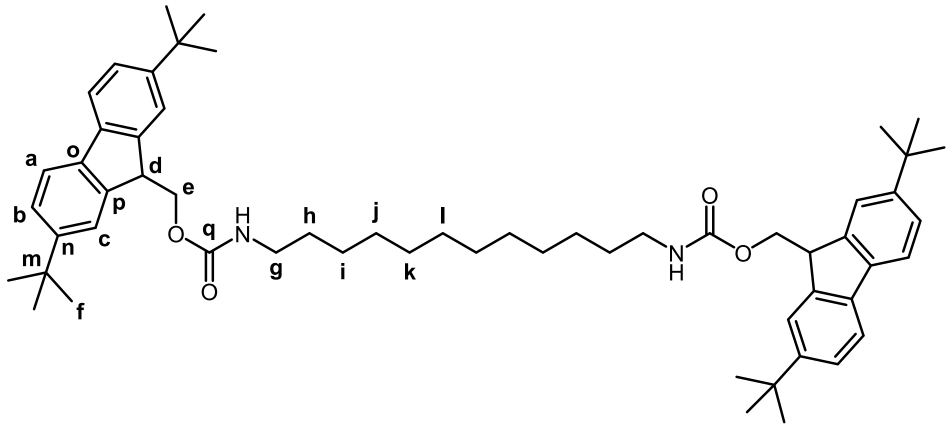

Under inert conditions, 1,12-diaminododecane (21.5 mg, 107.3 µmol, 1.0 eq.) and **Fmoc*-OSu** (121.1 mg, 269.3 µmol, 2.5 eq.) were weighed into a preheated Schlenk tube. Anhydrous acetonitrile (4.5 mL) was added, and the resulting colorless suspension was briefly ultrasonicated. The mixture was then stirred for 16 h under an inert atmosphere, forming a clear, colorless solution. After removing the solvent under reduced pressure, a colorless oil was obtained, containing primarily the desired product along with residual **Fmoc*-OSu** and, occasionally, unreacted diamine or **DBF***. The crude oil was purified by flash column chromatography (d = 2.5 cm, h = 20 cm; hexane/Et$_2$O = 5:1). After removing all volatiles under reduced pressure, the product was subjected to azeotropic drying with acetone (5 × 3 mL) and finally benzene to eliminate residual solvent. The product was then dried *in vacuo* for several days, yielding a colorless, amorphous solid in 87% yield (81.0 mg, 93.2 µmol).

^{1}H NMR (500.4 MHz, benzene-d_6, 300 K): δ [ppm] = 7.72 (s, 4H, H$_c$), 7.67 (d, ^3J = 8.0 Hz, 4H, H$_a$), 7.40 (dd, ^3J = 8.0 Hz, ^4J = 1.9 Hz, 4H, H$_b$), 4.66 (d, ^3J = 5.9 Hz, 4H, H$_e$), 4.18 (t, ^3J = 5.9 Hz, 2H, H$_d$), 3.97 (t, ^3J = 6.1 Hz, 2H, NH), 2.90 (q, ^3J = 6.8 Hz, 4H, H$_g$), 1.35 (s, 36H, H$_f$), 1.28–0.98 (m, 20H, H$_{h-l}$).

At 298 K, a second set of signals for H$_d$ (4.25 ppm), H$_e$ (4.60 ppm), and NH (4.09 ppm) suggests a conformational change in the Fmoc* moiety that occurs more slowly than the timescale of the ^{1}H NMR experiment. This is corroborated by variable temperature NMR studies. With increasing temperature, the pairs of resonances coalesce into a single set of signals, highlighting the dynamic nature of the conformational equilibrium.

^{13}C NMR (125.8 MHz, benzene-d_6, 300 K): δ [ppm] = 156.3 (C$_q$), 150.0 (C$_n$), 144.9 (C$_p$), 139.5 (C$_o$), 125.1 (C$_b$), 122.1 (C$_c$), 119.7 (C$_a$), 66.2 (C$_e$), 48.3 (C$_d$), 41.1 (C$_g$), 35.0 (C$_m$), 31.8 (C$_f$), 30.3 (C$_{h-l}$), 30.01 (C$_{h-l}$), 29.96 (C$_{h-l}$), 29.7 (C$_{h-l}$), 26.9 (C$_{h-l}$).

EA (%) for **[Fmoc*-NH-(CH$_2$)$_6$]$_2$** · 0.1 HOSu: calc. C 79.64, H 9.21, N 3.34; found C 79.56, H 9.11, N 3.25.

TLC: R_f = 0.74 (hexane/EtOAc = 3:1) [UV, KMnO$_4$].

2,7-Di-*tert*-butyl-dibenzofulvene (DBF*)

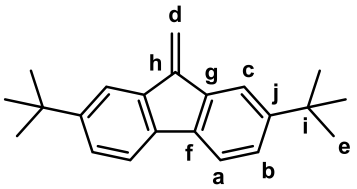

The synthesis of 2,7-di-*tert*-butyl-dibenzofulvene (**DBF***) was adapted from a previously reported method for the preparation of dibenzofulvene (DBF) from 9-fluorenylmethanol.[6]

Under ambient conditions, 2,7-di-*tert*-butyl-9-fluorenylmethanol (150.0 mg, 486.3 µmol, 1.0 eq.) and finely ground potassium hydroxide (96.3 mg, 85 wt%, 1.46 mmol, 3.0 eq.) were weighed into a glass vial. Anhydrous methanol (3 mL) was added, and the resulting suspension was heated to 60 °C. The reaction mixture was stirred at this temperature for 2 h until thin-layer-chromatography (TLC) indicated complete consumption of 2,7-di-*tert*-butyl-9-fluorenylmethanol. During the reaction, precipitation of insoluble **DBF*** polymer was observed. The reaction mixture was poured into a separatory funnel, diluted with hexane (25 mL) and washed with water until the wash water reached a neutral pH, indicating the complete removal of excess KOH. The organic layer was filtered over $MgSO_4$ to remove traces of water and residual polymeric precipitate. The resulting faint yellow filtrate was concentrated to dryness, and the product was dried *in vacuo* for 3 h, yielding off-white to faint yellow crystals in 90% yield (127.7 mg, 439.7 µmol). **DBF*** is prone to polymerization and was thus stored under the exclusion of light at –40 °C.

^{1}H NMR (500.4 MHz, MeCN-d_3, 300 K): δ [ppm] = 7.87 (d, ^4J = 1.9 Hz, 2H, H_c), 7.63 (d, ^3J = 8.0 Hz, 2H, H_a), 7.45 (dd, ^3J = 8.0 Hz, ^4J = 1.9 Hz, 2H, H_b), 6.19 (s, 2H, H_d), 1.37 (s, 18H, H_e).

^{13}C NMR (125.8 MHz, MeCN-d_3, 300 K): δ [ppm] = 151.2 (C_j), 144.7 (C_h), 139.0 (C_g), 138.4 (C_f), 127.1 (C_b), 120.2 (C_a), 119.0 (C_c), 108.4 (C_d), 35.6 (C_i), 31.7 (C_e).

EA (%): calc. C 90.98, H 9.02; found C 91.31, H 8.69.

TLC: R_f = 0.40 (hexane) [UV, $KMnO_4$].

[Fmoc*-Rot][Ag_8L_2](PF_6)$_4$

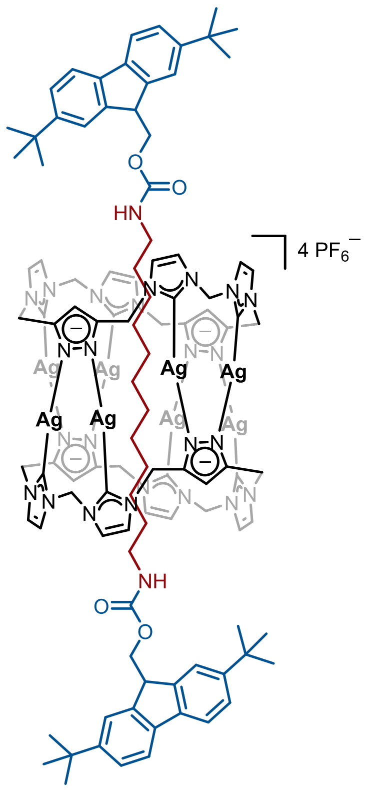

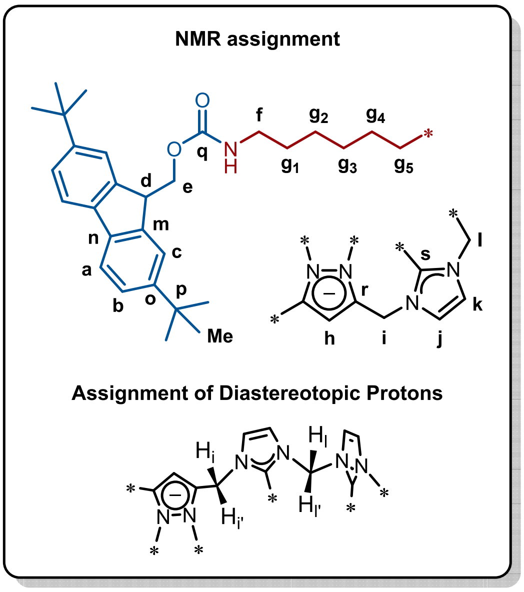

Under inert conditions, pillarplex **[Ag_8L_2](PF_6)$_4$** (100.0 mg, 41.7 µmol, 1.0 eq.) and 1,12-diaminododecane (19.2 mg, 95.8 µmol, 2.3 eq.) were weighed into a preheated Schlenk tube and suspended in anhydrous acetonitrile (10 mL). The mixture was briefly ultrasonicated and then stirred for 30 min under the exclusion of light to facilitate insertion of the diamine into the pillarplex pore. **Fmoc*-OSu** (86.2 mg, 191.8 µmol, 4.6 eq.) was subsequently added, and the mixture was stirred for an additional 16 h under the exclusion of light. Notably, the succinimidyl carbonate was chosen over the corresponding chloroformate (**Fmoc*-Cl**) to prevent the release of chloride ions near the Ag^{I}-based cavitand, which could otherwise cause decomposition by formation of AgCl. The resulting pale yellow, clear solution was transferred to a round-bottom flask, and diethyl ether (200 mL) was added to precipitate a white solid. The crude product was collected by filtration and further purified by washing with diethyl ether (10 mL) and hexane (3 × 10 mL), followed by a fractional precipitation from acetone with diethyl ether. After drying *in vacuo*, **[Fmoc*-Rot][Ag_8L_2](PF_6)$_4$** was obtained as a white solid in 68% yield (93.2 mg, 28.5 µmol).

^{1}H NMR (500.1 MHz, MeCN-d_3, 298 K): δ [ppm] = 7.82 (s, 4H, H_c), 7.76 (d, ^3J = 8.0 Hz, 4H, H_a), 7.55 (dd, ^3J = 8.0 Hz, ^4J = 2.0 Hz, 4H, H_b), 7.53 (*virt.* t, $^3J \approx {}^4J$ = 1.9 Hz, 8H, H_k), 7.19 (*virt.* t, $^3J \approx {}^4J$ = 1.9 Hz, 8H, H_j), 6.58 (d, ^2J = 14.9 Hz, 4H, $H_{l'}$), 6.43 (s, 4H, H_h), 5.99 (d, ^2J = 14.9 Hz, 4H, H_l), 5.53 (d, ^2J = 15.3 Hz, 8H, $H_{i'}$), 5.07 (d, ^2J = 15.2 Hz, 8H, H_i), 4.68 (d, ^3J = 5.5 Hz, 4H, H_e), 4.42 (t, ^3J = 5.5 Hz, 2H, NH), 4.32 (t, ^3J = 5.5 Hz, 2H, H_d), 2.18 (q, ^3J = ^3J = 6.1 Hz, 4H, H_f), 1.40 (s, 36H, H_{Me}), –0.27 (br s, 8H, H_g), –0.65 (br s, 4H, H_g), –1.03 (br s, 8H, H_g).

^{1}**H NMR** (500.1 MHz, acetone-d_6, 298 K): δ [ppm] = 7.97 (s, 8H, H_k), 7.84 (s, 4H, H_c), 7.80 (d, ^{3}J = 8.0 Hz, 4H, H_a), 7.54 (dd, ^{3}J = 8.0 Hz, ^{4}J = 1.5 Hz, 4H, H_b), 7.53 (s, 8H, H_j), 7.08 (d, ^{2}J = 14.8 Hz, 4H, $H_{l'}$), 6.67 (s, 4H, H_h), 6.42 (d, ^{2}J = 14.8 Hz, 4H, H_l), 5.82 (d, ^{2}J = 15.1 Hz, 8H, $H_{i'}$), 5.29 (d, ^{2}J = 15.1 Hz, 8H, H_i), 5.23 (t, ^{3}J = 5.5 Hz, 2H, NH), 4.69 (d, ^{3}J = 5.5 Hz, 4H, H_e), 4.31 (t, ^{3}J = 5.5 Hz, 2H, H_d), 2.33 (m, 4H, H_f), 1.39 (s, 36H, H_{Me}), 0.18 (bs, 4H, H_{g5}), –0.03 (bs, 4H, H_{g1}), –0.37 (bs, 4H, H_{g4}), –0.89 (bs, 8H, H_{g2} and H_{g3}).

^{13}**C NMR** (125.8 MHz, MeCN-d_3, 300 K): δ [ppm] = 179.0 (d, $^{1}J_{109Ag–13C}$ = 270.0 Hz, $^{1}J_{107Ag–13C}$ = 234.1 Hz, C_s), 156.9 (C_q), 151.3 (C_o), 150.0 (*virt.* t, $^{2}J_{109Ag-13C}$ = 13.3 Hz, C_r), 145.6 (C_m), 139.8 (C_n), 126.0 (C_b), 124.6 (d, $^{3}J_{109Ag-13C}$ = 7.5 Hz, C_j), 123.5 (d, $^{3}J_{109Ag–13C}$ = 6.4 Hz, C_k), 122.9 (C_c), 120.6 (C_a), 104.0 (C_h), 66.6 (C_e), 65.4 (C_l), 49.4 (C_i), 48.4 (C_d), 41.2 (C_f), 35.7 (C_p), 33.6 (C_g), 32.5 (C_g), 31.9 (C_{Me}), 31.3 (C_g), 30.0 (C_g), 27.3 (C_g).

^{13}**C NMR** (125.8 MHz, acetone-d_6, 300 K): δ [ppm] = 179.2 (d, $^{1}J_{109Ag–13C}$ = 269.7 Hz, $^{1}J_{107Ag–13C}$ = 234.0 Hz, C_s), 156.9 (C_q), 150.9 (C_o), 150.1 (*virt.* t, $^{2}J_{109Ag-13C}$ = 13.1 Hz, C_r), 145.5 (C_m), 139.8 (C_n), 125.8 (C_b), 124.6 (bs, C_j), 123.7 (bs, C_k), 122.7 (C_c), 120.4 (C_a), 104.2 (C_h), 66.6 (C_e), 65.4 (C_l), 49.3 (C_i), 48.3 (C_d), 41.3 (C_f), 35.6 (C_p), 34.1 (C_{g5}), 32.8 (C_{g4}), 31.9 (C_{Me}), 31.2 (C_{g2} or C_{g3}), 30.4 (C_{g1}), 27.5 (C_{g2} or C_{g3}).

^{19}**F NMR** (470.8 MHz, MeCN-d_3, 300 K): δ [ppm] = –72.7 (d, ^{1}J = 706.6 Hz, PF_6).

^{19}**F NMR** (470.8 MHz, acetone-d_6, 300 K): δ [ppm] = –72.0 (d, ^{1}J = 708.7 Hz, PF_6).

^{31}**P NMR** (202.6 MHz, MeCN-d_3, 300 K): δ [ppm] = –144.9 (hept, ^{1}J = 706.6 Hz, PF_6).

^{31}**P NMR** (202.6 MHz, acetone-d_6, 300 K): δ [ppm] = –144.2 (hept, ^{1}J = 708.7 Hz, PF_6).

EA (%) for **[Fmoc*-Rot][Ag_8L_2](PF_6)$_4$** · 0.5 Et_2O: calc. C 39.23, H 3.93, N 11.01; found C 39.04, H 4.01, N 11.21. Even after prolonged times of drying *in vacuo*, ~0.5 eq. of Et_2O remained in the sample (*cf.* Figure S16).

[Amide-Rot][Ag$_8$L$_2$](PF$_6$)$_4$

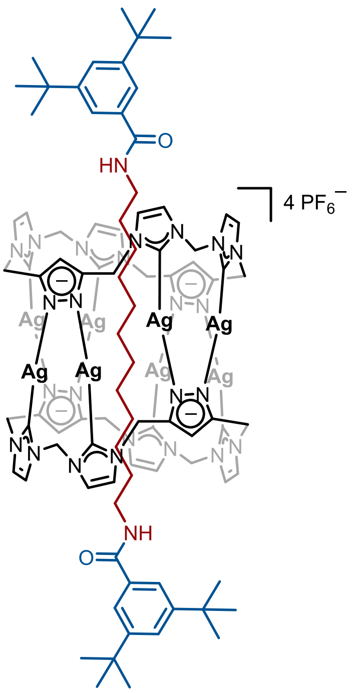

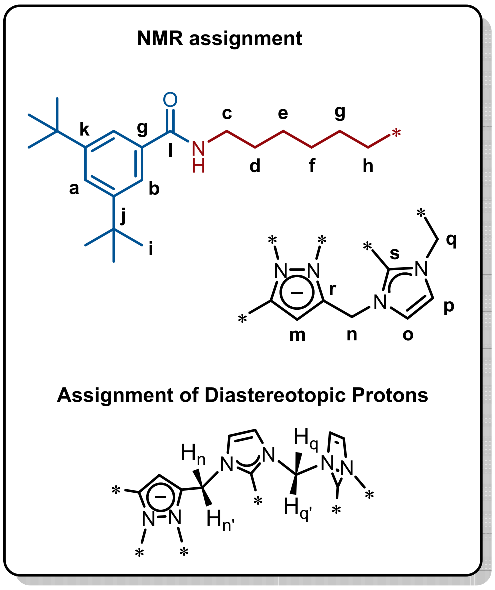

Under inert conditions, pillarplex **[Ag$_8$L$_2$](PF$_6$)$_4$** (52.0 mg, 21.7 µmol, 1.0 eq.) and 1,12-diaminododecane (10.9 mg, 54.2 µmol, 2.5 eq.) were weighed into a preheated Schlenk tube and suspended in anhydrous acetonitrile (2.5 mL). The mixture was briefly ultrasonicated and then stirred for 30 min under the exclusion of light to ensure insertion of the diamine into the pillarplex pore. Subsequently, *N*,*N*-diisopropylethylamine (18.9 µL, 108.3 µmol, 5.0 eq.) and 3,5-di-*tert*-butylbenzoic anhydride (48.8 mg, 108.3 µmol, 5.0 eq.) were added, and the reaction mixture was stirred for an additional 16 h under the exclusion of light. The resulting colorless, clear solution was transferred to a conical tube, and diethyl ether (45 mL) was added to precipitate an off-white solid. The crude product was washed with diethyl ether (15 mL) and further purified by fractional precipitation from acetone with diethyl ether. The resulting white solid was then repeatedly washed with diethyl ether until the ethereal layer no longer contained any dissolved organic salts.† After drying *in vacuo*, **[Amide-Rot][Ag$_8$L$_2$](PF$_6$)$_4$** was obtained as a white solid in 50% yield (33 mg, 10.9 µmol).

^{1}H NMR (500.1 MHz, MeCN-*d*$_3$, 298 K): δ [ppm] = 7.73 (d, ^4J = 1.7 Hz, 4H, H$_b$), 7.72 (t, ^4J = 1.7 Hz, 2H, H$_a$), 7.66 (t, $^3J \approx {}^4J$ = 1.9 Hz, 8H, H$_p$), 7.48 (*virt.* t, $^3J \approx {}^4J$ = 1.9 Hz, 8H, H$_o$), 6.83 (t, NH, 2H, ^3J = 5.8 Hz, NH), 6.68 (s, 4H, H$_m$), 6.63 (d, ^2J = 14.9 Hz, 4H, H$_{q'}$), 6.06 (d, ^2J = 14.9 Hz, 4H, H$_q$), 5.62 (d, ^2J = 15.2 Hz, 4H, H$_{n'}$), 5.24 (d, ^2J = 15.2 Hz, 4H, H$_n$), 2.58 (m, 4H, H$_c$), 1.43 (s, 36H, H$_i$), 0.31 (m, 4H, H$_h$), 0.02 (m, 4H, H$_d$), –0.67 (m, 4H, H$_g$), –1.21 (m, 4H, H$_e$), –1.46 (m, 4H, H$_f$).

^{1}H NMR (500.1 MHz, acetone-*d*$_6$, 298 K): δ [ppm] = 8.05 (s, 8H, H$_p$), 7.82 (d, ^4J = 1.6 Hz, 4H, H$_b$), 7.79 (s, 8H, H$_o$), 7.73 (t, ^4J = 1.6 Hz, 2H, H$_a$), 7.54 (t, NH, 2H, ^3J = 5.6 Hz, NH), 7.11 (d,

† likely *N*,*N*-diisopropylethylammonium 3,5-di-*tert*-butylbenzoate

^2J = 14.8 Hz, 4H, $H_{q'}$), 6.91 (s, 4H, H_m), 6.48 (d, ^2J = 14.9 Hz, 4H, H_q), 5.89 (d, ^2J = 15.2 Hz, 4H, $H_{n'}$), 5.46 (d, ^2J = 15.2 Hz, 4H, H_n), 2.70 (m, 4H, H_c), 1.41 (s, 36H, H_i), 0.63 (m, 4H, H_h), 0.23 (m, 4H, H_d), –0.37 (m, 4H, H_g), –1.00 (m, 4H, H_e), –1.21 (m, 4H, H_f).

^{13}C NMR (125.8 MHz, MeCN-d_3, 300 K): δ [ppm] = 179.1 (d, $^1J_{109Ag–13C}$ = 251.2 Hz, $^1J_{107Ag–13C}$ = 233.1 Hz, C_s), 168.3 (C_l), 152.4 (C_k), 150.0 (*virt.* t, $^2J_{109Ag-13C} \approx {}^3J_{109Ag-13C}$ = 12.5 Hz, C_r), 135.8 (C_g), 126.6 (C_a), 124.7 (d, $^3J_{109Ag–13C}$ = 6.3 Hz, C_o), 123.6 (d, $^3J_{109Ag–13C}$ = 5.9 Hz, C_p), 122.1 (C_b), 104.1 (C_m), 65.5 (C_q), 49.5 (C_n), 40.6 (C_c), 35.8 (C_j), 34.8 (C_h), 32.8 (C_g), 31.6 (C_i), 31.0 (C_d), 30.4 (C_f), 27.5 (C_e).

^{19}F NMR (470.8 MHz, MeCN-d_3, 300 K): δ [ppm] = –72.8 (d, ^1J = 706.8 Hz, PF_6).

^{31}P NMR (202.6 MHz, MeCN-d_3, 300 K): δ [ppm] = –144.6 (hept, ^1J = 706.5 Hz, PF_6).

EA (%) for **[Amide-Rot][Ag_8L_2](PF_6)$_4$** · 0.3 Et_2O: calc. C 35.85, H 3.79, N 11.92; found C 35.69, H 4.03 , N 11.58. Even after prolonged times of drying *in vacuo*, ~0.3 eq. of Et_2O remained in the sample (*cf.* Figure S34).

HR-HESI-MS (*m/z*): calc. 144.9636 [PF_6]$^-$, 613.0458 [Amide-Rot][Ag_8L_2]$^{4+}$, 866.0505 [Amide-Rot][Ag_8L_2](PF_6)$^{3+}$, 1371.5581 [Amide-Rot][Ag_8L_2](PF_6)$_2^{2+}$;
found 144.9633 [PF_6]$^-$, 613.0432 [Amide-Rot][Ag_8L_2]$^{4+}$, 866.0477 [Amide-Rot][Ag_8L_2](PF_6)$^{3+}$, 1371.5567 [Amide-Rot][Ag_8L_2](PF_6)$_2^{2+}$.

4. Kinetic Studies

The Fmoc* deprotection kinetics of **[Fmoc*-Rot][Ag_8L_2](PF_6)$_4$** and **[Fmoc*-NH-(CH_2)$_6$]$_2$** were studied using ^{1}H NMR spectroscopy.

Sample preparation

All sample preparation steps, except for the final addition of piperidine, were performed under inert conditions in an *MBraun* Labmaster Pro SP glovebox to prevent contamination from moisture and oxygen. The following procedure was applied for each kinetic experiment:

Inside the glovebox, approximately 3.2 µmol of either **[Fmoc*-Rot][Ag_8L_2](PF_6)$_4$** or **[Fmoc*-NH-(CH_2)$_6$]$_2$** in case of were accurately weighed into a J. Young NMR tube using an analytical balance (precision ±0.1 mg). Anhydrous acetone-*d*$_6$ (~520 mg, 0.6 mL) was added to the NMR tube gravimetrically as the solvent. A freshly prepared stock solution of 1,4-dioxane in acetone-*d*$_6$ (~100–200 mM) was added to the tube in approximately equimolar ratio to the Fmoc* substrate as an internal standard (^{1}H NMR chemical shift in acetone-*d*$_6$: δ_{IS} = 3.59 ppm). The precise molar ratio of the Fmoc* substrate to the IS was then determined by ^{1}H NMR.

A stock solution of piperidine in acetone-*d*$_6$ (~3 M) was freshly prepared, and 15–20 equivalents of piperidine (relative to the Fmoc* substrate) were added to the reaction gravimetrically. Before the addition, the NMR tube was positioned in the NMR spectrometer, with the magnetic field locked and shimming completed, to minimize any delay in measurement. After adding the piperidine stock solution, the tube was brief shaken and promptly placed in the spectrometer for the first ^{1}H NMR acquisition. The total time between the base addition and the start of the first measurement was approximately 30–60 seconds, and this delay was accounted for during data processing.

NMR data acquisition and processing

^{1}H NMR spectra were collected using a *Bruker* AV-400 spectrometer in acetone-*d*$_6$ at 20°C (±1°C). Data were recorded in intervals of ~3.9 minutes for **[Fmoc*-Rot][Ag_8L_2](PF_6)$_4$** and ~9.6 minutes for **[Fmoc*-NH-(CH_2)$_6$]$_2$** (including the time required for the experiment itself). For each spectrum, 32 scans were measured with an acquisition time of 4.0 seconds (total data acquisition time ~2.8 minutes).

NMR data processing was performed in *MestReNova* 15.0.1 following the same steps:

1. Each raw spectrum was processed *via* the “advised processing” routine as implemented in *MestReNova*, including the application of an apodization function along T_1 (Stanning 8), group delay (linear phase shift), and phase correction (regions analysis).
2. Exponential line broadening of 0.3 Hz was applied to enhance signal-to-noise ratio.
3. Then the spectra were referenced to the residual solvent signal of acetone-*d*$_6$.
4. Baseline correction (“ablative” mode with 3 points and 10 passes) was applied.

The well-resolved methine proton of the Fmoc* group (δ_{CH} ~ 4.2–4.3 ppm) was selected to track the deprotection kinetics, as no interfering signals from by-products or intermediates were observed in this spectral region. To ensure accurate quantification of the Fmoc* methine proton integral throughout the reaction, the integral of the internal standard (IS) was normalized to the value determined prior to piperidine addition.

The integrated methine proton signals were converted to molar concentrations based on the initial amount of Fmoc* substrate weighed into the NMR tube. These concentrations were then plotted against time (as obtained from the timestamps after each measurement). The concentration *vs.* time plots are shown in Figure S2 and Figure S3.

Determination of kinetic parameters

Mechanistic assumptions

Based on prior literature on Fmoc reactivity,[7-8] the deprotection of Fmoc* by piperidine in polar solvents is expected to follow the mechanism depicted in Figure S1.

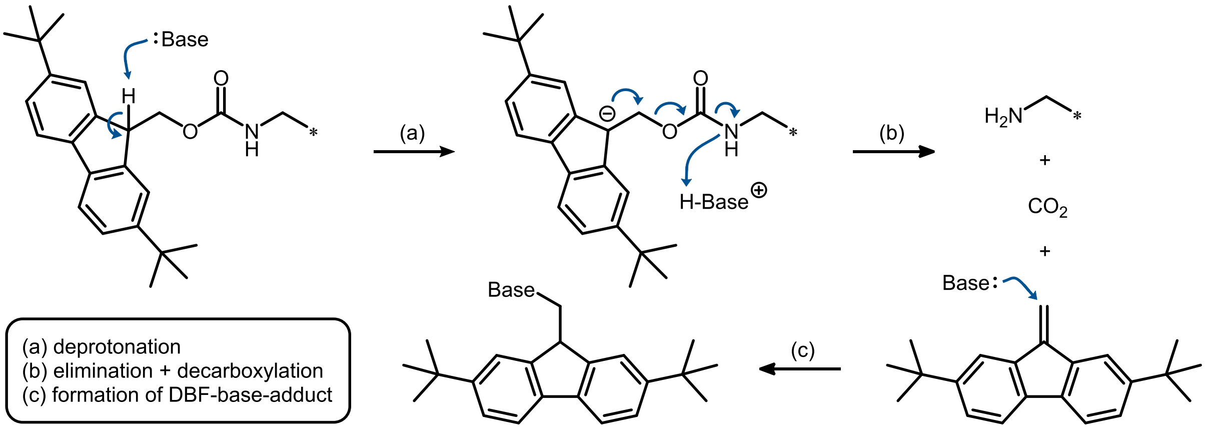

Figure S1 | Proposed mechanism for Fmoc* deprotection. The adduct formation between **DBF*** and piperidine as base (reaction c) was observed to be negligibly slow under the chosen reaction conditions.

It is generally accepted that the rate-determining step in Fmoc deprotection in moderately polar solvents is the initial abstraction of the acidic methine proton attached to the fluorenyl group by a base,[7-8] typically a secondary amine like piperidine. This generates a carbanion intermediate, which rapidly undergoes decarboxylation, liberating the protected amine in the process. Assuming that the initial deprotonation is quasi-irreversible and the subsequent elimination step is instantaneous, the rate law for the analogous Fmoc* deprotection reaction can be expressed as follows:

$$r = -\frac{d[\text{Fmoc}^*]}{dt} = -k[\text{piperidine}][\text{Fmoc}^*]$$

where, [Fmoc*] represents the molar concentration of **[Fmoc*-Rot][Ag$_8$L$_2$](PF$_6$)$_4$** or **[Fmoc*-NH-(CH$_2$)$_6$]$_2$** (M) at time *t* (h), [piperidine] is the molar concentration of piperidine (M) at time *t*,

and k is the rate constant of the reaction ($M^{-1}h^{-1}$). The reaction follows first-order kinetics with respect to both Fmoc* and piperidine, giving an overall reaction order of 2. However, if piperidine is used in large excess relative to the Fmoc* substrate, the reaction kinetics simplify and can be described by a pseudo-1st order rate law:

$$r = -k[\text{piperidine}][\text{Fmoc}^*] = -k[\text{piperidine}]_0[\text{Fmoc}^*] = -k'[\text{Fmoc}^*]$$

Here, k' is the pseudo-1st order rate constant (h^{-1}), defined as the product of the initial base concentration, $[\text{piperidine}]_0$, and the 2nd order rate constant k. The integrated form of the rate equation is:

$$[\text{Fmoc}^*] = [\text{Fmoc}^*]_0 \cdot e^{-k't}$$

Kinetic fitting

To determine k', the time-dependent concentration data, obtained from ^{1}H NMR integrations, were exported to *OriginPro* 2020 for kinetic fitting. The data were truncated at 90% conversion of **[Fmoc*-Rot][Ag_8L_2](PF_6)$_4$** and **[Fmoc*-NH-(CH_2)$_6$]$_2$** to avoid overfitting of noise, which interferes with accurate integration of the Fmoc* methine signal at low concentrations. The pseudo-rate constant k' was extracted by fitting the concentration-time data to a mono-exponential decay function:

$$y = A \cdot e^{bx} + y_0$$

Here, A represents the initial concentration $[\text{Fmoc}^*]_0$ and b corresponds to $-k'$. The deprotection kinetics of both, **[Fmoc*-Rot][Ag_8L_2](PF_6)$_4$** and **[Fmoc*-NH-(CH_2)$_6$]$_2$** were each measured at three different piperidine concentrations (ranging from ~0.07 M to ~0.12 M) to verify 1st order behavior with respect to the base. For each base concentration level, the individual k' values were determined. In all cases, the concentration-time data showed excellent agreement with the fitted function, with R^2 values of 0.996 or higher. This strongly corroborates that the system operates under pseudo-1st order kinetics.

Determination of rate constants

Once k' was determined, the 2nd order rate constant k for the Fmoc* deprotection reaction was calculated using the following relationship:

$$k = \frac{k'}{[\text{piperidine}]_0}$$

For each NMR experiment, k was calculated and averaged across the different base concentrations for both **[Fmoc*-Rot][Ag_8L_2](PF_6)$_4$** and **[Fmoc*-NH-(CH_2)$_6$]$_2$**. To account for variability in the measurements, 95% confidence intervals (CIs) were calculated using the *t*-distribution, given the small sample size (n = 3):

$$\mathrm{CI} = k_{\mathrm{ave}} \pm t_{2,0.025} \cdot \frac{\sigma}{\sqrt{n}}$$

where, k_{ave} is the average rate constant, σ is the standard deviation of the rate constants, and $t_{2,0.025}$ is the t-value for two (n–1) degrees of freedom at a 95% confidence level, and n corresponds to the number of replicates for the deprotection of either **[Fmoc*-Rot][Ag_8L_2](PF_6)$_4$** or **[Fmoc*-NH-(CH_2)$_6$]$_2$**. The data are summarized in Table S1–2.

As a control experiment, the deprotection of **[Fmoc*-NH-(CH_2)$_6$]$_2$** was also followed kinetically in the presence of roughly equimolar **[Amide-Rot][Ag_8L_2](PF_6)$_4$** to assess the influence of a pillarplex rotaxane (whose stopper cannot be cleaved by base) on the reaction kinetics, *i.e.* to study whether the reaction kinetics can be accelerated intermolecularly by pillarplexes. The data were processed in analogy to the described experiments (*vide supra*) and are summarized in Table S3.

Table S1 | Experimental and fitted kinetic parameters for the Fmoc* deprotection of **[Fmoc*-Rot][Ag_8L_2](PF_6)$_4$**.

	[Fmoc*]$_0$ / M	[piperidine]$_0$ / M	R^2	k' / h^{-1}	k / $M^{-1}h^{-1}$	k_{ave} / $M^{-1}h^{-1}$	σ	$CI_{95\%}$	Yield
1.	$5.95 \cdot 10^{-3}$	$9.90 \cdot 10^{-2}$	0.9977	2.07	20.95	20.5	1.5	[16.9, 24.1]	67%
2.	$4.81 \cdot 10^{-3}$	$8.68 \cdot 10^{-2}$	0.9988	1.64	18.86				82%
3.	$5.80 \cdot 10^{-3}$	$1.36 \cdot 10^{-1}$	0.9965	2.95	21.69				79%

Note: The yield refers to the amount of DBF formed at 90% conversion of **[Fmoc*-Rot][Ag_8L_2](PF_6)$_4$** (as the only detectible product during the kinetics. Once the characteristic Fmoc* C^9–H signal vanished over time, only DBF* remained. No piperidine adduct of DBF* was observed over the course of the measurements.*

Table S2 | Experimental and fitted kinetic parameters for the Fmoc* deprotection of **[Fmoc*-NH-(CH_2)$_6$]$_2$**.

	[Fmoc*]$_0$ / M	[piperidine]$_0$ / M	R^2	k' / h^{-1}	k / $M^{-1}h^{-1}$	k_{ave} / $M^{-1}h^{-1}$	σ	$CI_{95\%}$	Yield
1.	$5.24 \cdot 10^{-3}$	$1.06 \cdot 10^{-1}$	0.9992	$7.40 \cdot 10^{-2}$	0.69	0.56	0.12	[0.27, 0.85]	59%
2.	$5.16 \cdot 10^{-3}$	$8.42 \cdot 10^{-2}$	0.9995	$4.28 \cdot 10^{-2}$	0.51				54%
3.	$7.23 \cdot 10^{-3}$	$1.22 \cdot 10^{-1}$	0.9992	$5.92 \cdot 10^{-2}$	0.48				58%

Note: The yield refers to the amount of DBF formed at 90% conversion of **[Fmoc*-Rot][Ag_8L_2](PF_6)$_4$** (as the only detectible product during the kinetics. Once the characteristic Fmoc* C^9–H signal vanished over time, only DBF* remained. No piperidine adduct of DBF* was observed over the course of the measurements.*

Table S3 | Experimental and fitted kinetic parameters for the Fmoc* deprotection of **[Fmoc*-NH-(CH_2)$_6$]$_2$** in presence of **[Amide-Rot][Ag_8L_2](PF_6)$_4$** (~0.9 eq.).

	[Fmoc*]$_0$ / M	[piperidine]$_0$ / M	[amide-rotaxane]$_0$ / M	R^2	k' / h^{-1}	k / $M^{-1}h^{-1}$	Yield
1.	$1.02 \cdot 10^{-2}$	$1.83 \cdot 10^{-1}$	$9.38 \cdot 10^{-3}$	0.9993	$1.25 \cdot 10^{-1}$	0.68	53%

Note: The yield refers to the amount of DBF formed at 90% conversion of **[Fmoc*-Rot][Ag_8L_2](PF_6)$_4$** (as the only detectible product during the kinetics. Once the characteristic Fmoc* C^9–H signal vanished over time, only DBF* remained. No piperidine adduct of DBF* was observed over the course of the measurements.*

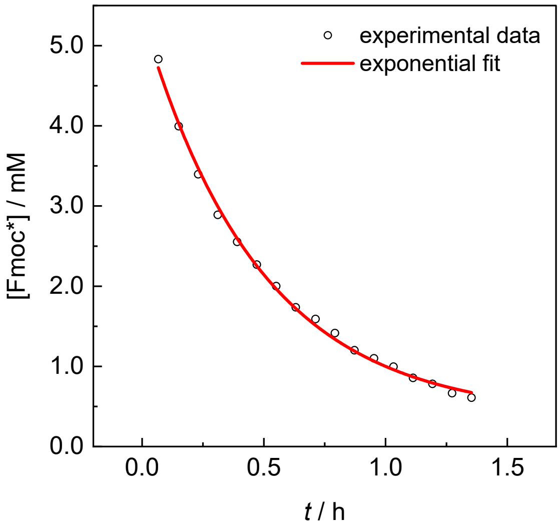

Model	exponential
Equation	y = y0 + A*exp(R0*x)
Plot	concentration vs. time
y0	3.72089E-4 ± 6.31762E-5
A	0.005 ± 6.37398E-5
R0	-2.07495 ± 0.08622
Reduced Chi-Sqr	3.98513E-9
R-Square (COD)	0.99773
Adj. R-Square	0.99741

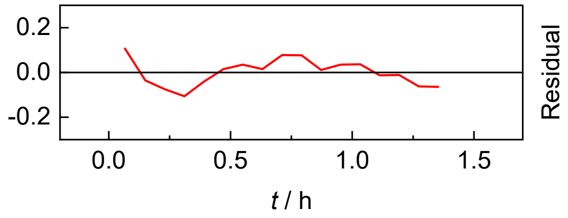

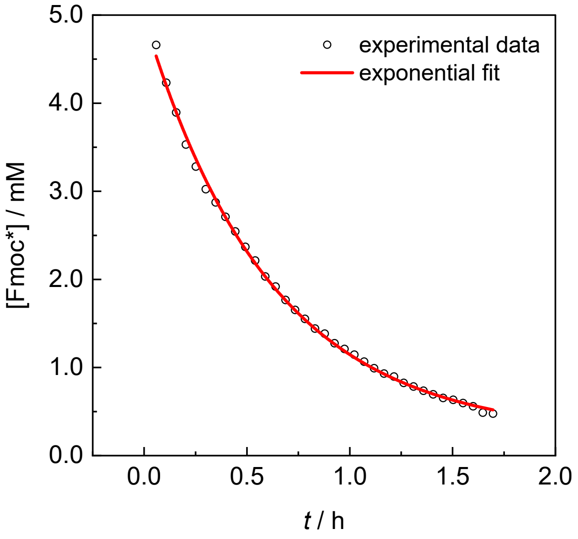

Model	exponential
Equation	y = y0 + A*exp(R0*x)
Plot	concentration vs. time
y0	2.23768E-4 ± 3.06525E-5
A	0.00474 ± 2.97606E-5
R0	-1.63662 ± 0.03386
Reduced Chi-Sqr	1.78715E-9
R-Square (COD)	0.99875
Adj. R-Square	0.99867

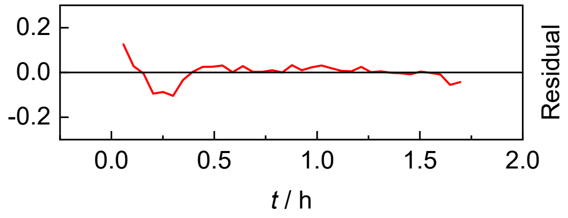

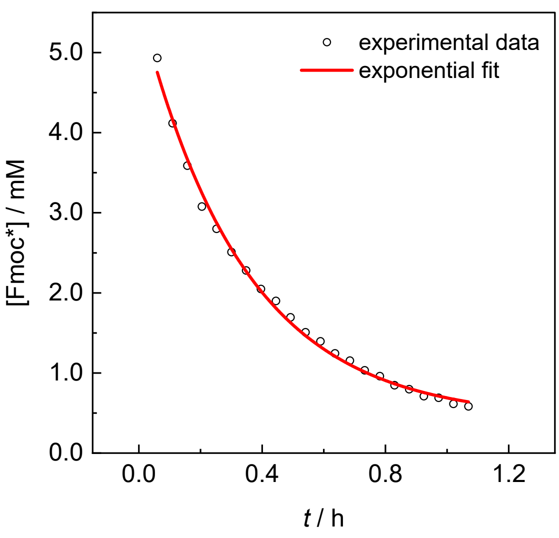

Model	exponential
Equation	y = y0 + A*exp(R0*x)
Plot	concentration vs. time
y0	4.19463E-4 ± 5.75571E-5
A	0.00517 ± 7.32611E-5
R0	-2.95382 ± 0.12295
Reduced Chi-Sqr	5.80568E-9
R-Square (COD)	0.99647
Adj. R-Square	0.9961

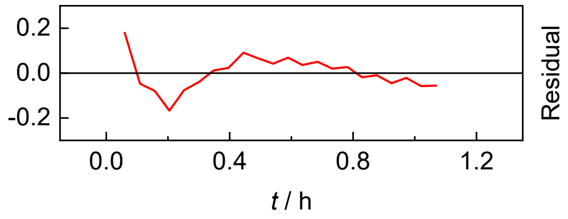

Figure S2 | Experimental data and exponential fit for the Fmoc* deprotection of **[Fmoc*-Rot][Ag_8L_2](PF_6)$_4$** at different piperidine concentrations: [piperidine]$_0$ = 9.90 · 10^{-2} M (top), 8.68 · 10^{-2} M (middle), 1.36 · 10^{-1} M (bottom).

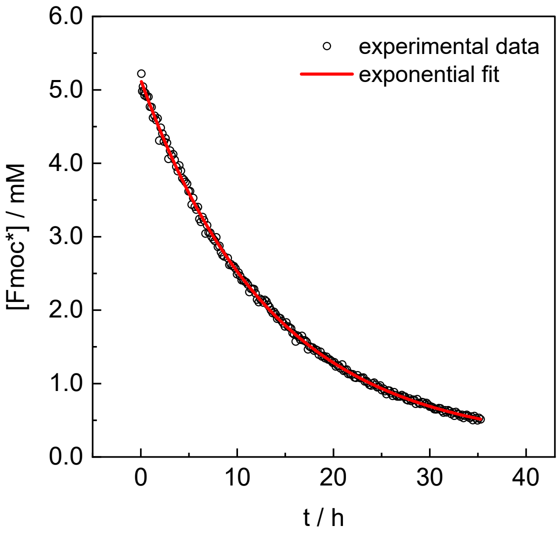

Model	exponential
Equation	y = y0 + A*exp(R0*x)
Plot	concentration vs. time
y0	1.48156E-4 ± 1.17808E-5
A	0.00499 ± 1.0761E-5
R0	-0.07399 ± 4.98137E-4
Reduced Chi-Sqr	1.45306E-9
R-Square (COD)	0.99916
Adj. R-Square	0.99915

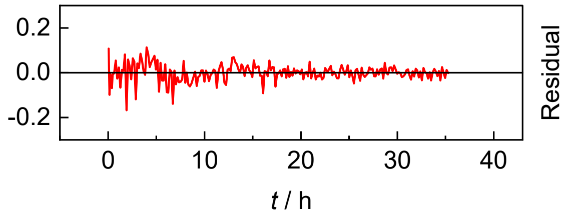

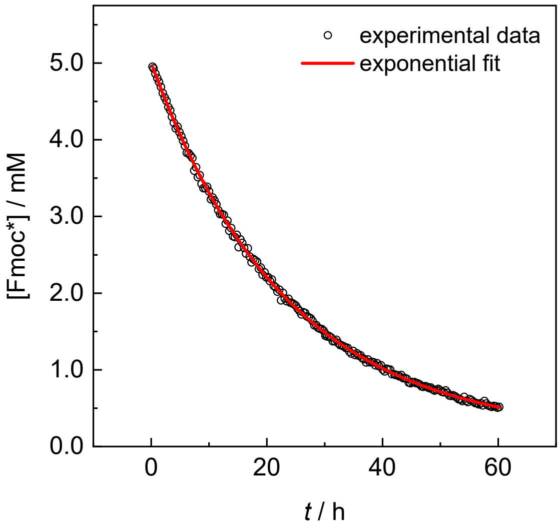

Model	exponential
Equation	y = y0 + A*exp(R0*x)
Plot	concentration vs. time
y0	1.4223E-4 ± 9.19196E-6
A	0.00486 ± 8.24954E-6
R0	-0.04282 ± 2.31524E-4
Reduced Chi-Sqr	7.08633E-10
R-Square (COD)	0.99954
Adj. R-Square	0.99954

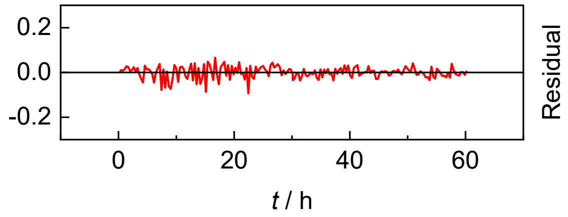

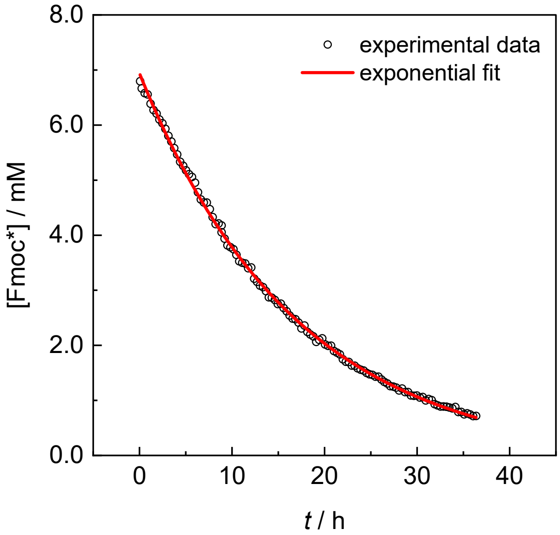

Model	exponential
Equation	y = y0 + A*exp(R0*x)
Plot	concentration vs. time
y0	-1.34357E-4 ± 3.0378E-5
A	0.00707 ± 2.53237E-5
R0	-0.05915 ± 6.29232E-4
Reduced Chi-Sqr	2.61082E-9
R-Square (COD)	0.99918
Adj. R-Square	0.99917

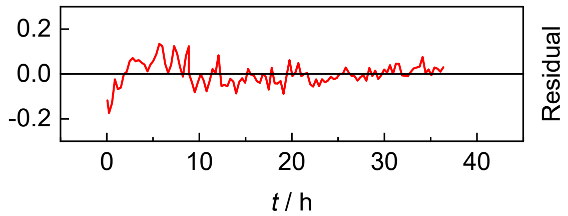

Figure S3 | Experimental data and exponential fit for the Fmoc* deprotection of **[Fmoc*-NH-$(CH_2)_6$]$_2$** at different piperidine concentrations: [piperidine]$_0$ = 1.06 · 10^{-1} M (top), 8.42 · 10^{-2} M (middle), 1.22 · 10^{-1} M (bottom).

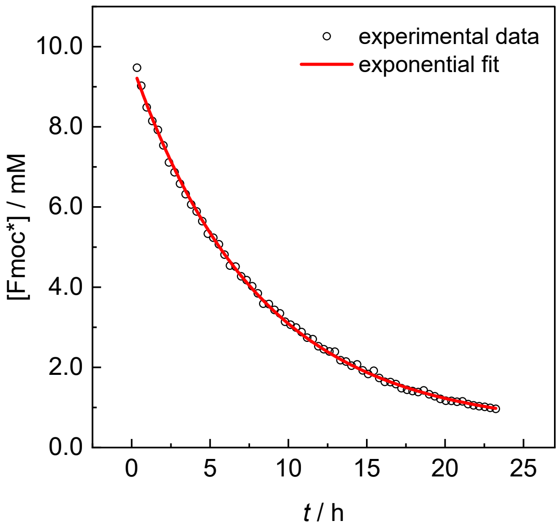

Model	exponential
Equation	y = y0 + A*exp(R0*x)
Plot	concentration vs. time
y0	4.80137E-4 ± 3.12728E-5
A	0.00911 ± 3.17541E-5
R0	-0.12508 ± 0.00137
Reduced Chi-Sqr	4.19333E-9
R-Square (COD)	0.99925
Adj. R-Square	0.99923

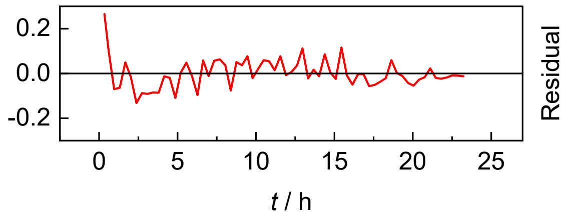

Figure S4 | Experimental data and exponential fit for the Fmoc* deprotection of **[Fmoc*-NH-$(CH_2)_6]_2$** in presence of **[Amide-Rot][Ag_8L_2]$(PF_6)_4$** (~0.9 eq.) at a piperidine concentration of $[\text{piperidine}]_0 = 1.83 \cdot 10^{-1}$ M.

5. Interproton Distance Determination by NMR

To quantify distances between spatially close protons in **[Fmoc*-Rot][Ag_8L_2](PF_6)$_4$**, a 2D ^{1}H,^{1}H ROESY spectrum was recorded in acetone-*d*$_6$ at 298 K on a *Bruker* AVHD-500 NMR spectrometer, using the *roesyphpp.2* pulse sequence (SW = 10.75 ppm, 1024 × 256 data points, NS = 24, DS = 32, D1 = 6 s, P15 = 1 s). The spectra were processed with a sine-square 90° apodization, followed by phase correction and baseline subtraction in both dimensions.

Prior to the ROESY experiment, proton T_1 values were determined *via* inversion recovery to optimize the relaxation delay. A mixing time of 1 s was found optimal for efficient ROE build-up while maintaining sufficient signal-to-noise ratio for reliable cross-peak integration. Since ROESY is less susceptible to spin diffusion than NOESY, this extended mixing time was considered appropriate for approximate distance quantification.

Reference interproton distances were selected based on the SC-XRD structure of rotaxane **[Fmoc*-Rot][Ag_8L_2](PF_6)$_4$** (solvatomorph A, chosen for its excellent data quality). Only protons in close proximity or in structurally rigid regions (*e.g.* aromatic/vicinal protons) were chosen to avoid conformational averaging (Table S4). The following proton pairs were excluded due to their unsuitability for correlation with solution-phase cross-peak integrals:

- Geminal proton pairs (H_i–$H_{i'}$ and H_l–$H_{l'}$), which are prone to TOCSY artifacts affecting the ROESY cross-peak intensities.[9-10]
- Proton pairs of structurally flexible regions (*e.g.* CH_2 protons in the linear alkyl chain of the inserted Fmoc*-protected amine), to avoid conformational averaging.
- Overlapping proton signals, where through-space coupling could interfere with distance quantification.

Interproton distances r_{ij} were estimated using the linear relation:

$$r_{ij} = k\left(a_{ij}c_{ij}\right)^{-\frac{1}{6}}$$

where a_{ij} is the uncorrected cross-peak integral of two spatially close protons *i* and *j*, and c_{ij} is a correction factor accounting for the offset dependence of the integral relative to the transmitter center (o1p).[11] The correction depends on the spin-lock power (γB_1 = 5000 Hz) and the difference between the chemical shift (ω_i) of the peak of interest and the transmitter offset (ω_0 = 1740.9 Hz), as follows:

$$c_{ij} = \frac{1}{\sin^2\theta_i \sin^2\theta_j} \qquad \text{where} \qquad \tan\theta_i = \frac{\gamma B_1}{\omega_i - \omega_0}$$

Plotting r_{ij} against $\left(a_{ij}c_{ij}\right)^{-\frac{1}{6}}$, a linear regression yielded the slope k (Å), which was then used to estimate unknown interproton distances (Table S4, Figure S5).

Table S4 | Averaged and corrected ^{1}H,^{1}H ROESY cross-peak integrals $a_{ij}c_{ij}$ (uncorrected intensities in brackets) and selected averaged SC-XRD distances (entries 1–6), as well as predicted interproton distances r_{ij} (entries A–C) based on linear regression. For proton nomenclature, see Section 3.

Entry	Proton pair	ROESY integral $a_{ij}c_{ij}$	SC-XRD distance r_{ij} / Å	Predicted distance r_{ij} / Å
1	a–b	6705.9 (4851.4)	2.34	2.35
2	c–d	1617.2 (1349.6)	3.01	2.98
3	h–i	1264.2 (1111.0)	2.98	3.10
4	i–j	3550.6 (2953.5)	2.64	2.61
5	j–k	6265.7 (4479.5)	2.46	2.38
6	k–l	3435.4 (2631.5)	2.66	2.63
A	a–h	190.7 (145.9)	–	4.26
B	a–i	108.1 (88.17)	–	4.68
C	e–k	97.2 (79.7)	–	4.76

Table S5 | Parameters used for the calculation of the correction factors c_{ij} of ROESY cross-peak integrals a_{ij}.

Proton	chemical shift δ / ppm	chemical shift ω / Hz	angle θ / rad
a	7.80	3901.0	1.163
b	7.54	3771.0	1.185
c	7.84	3921.0	1.160
d	4.31	2155.6	1.488
e	4.69	2345.6	1.450
h	6.67	3335.9	1.262
i'	5.82	2910.8	1.341
i	5.29	2645.7	1.392
j	7.53	3766.0	1.186
k	7.97	3986.0	1.149
l'	7.08	3540.9	1.225
l	6.42	3210.8	1.285

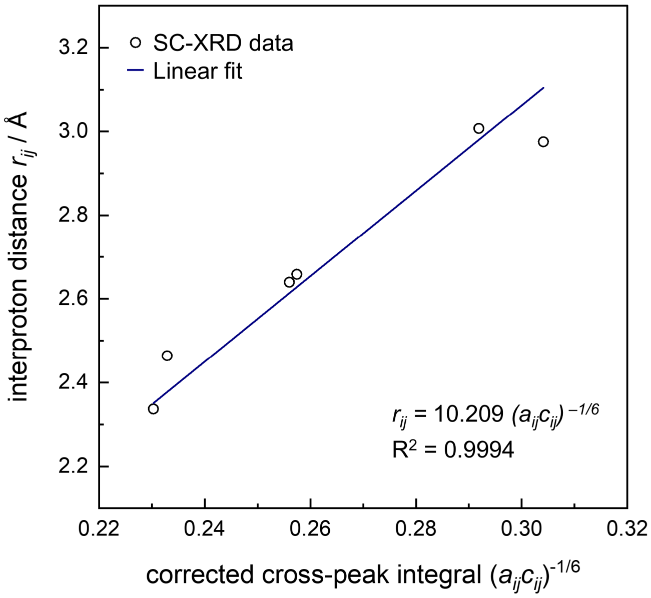

Figure S5 | Selected SC-XRD interproton distances r_{ij} plotted against ROESY cross-peak integral term $(a_{ij}c_{ij})^{-\frac{1}{6}}$. Linear regression (blue line) shows good correlation between the solid-state and solution data.

6. NMR Spectra

Fmoc*-OSu

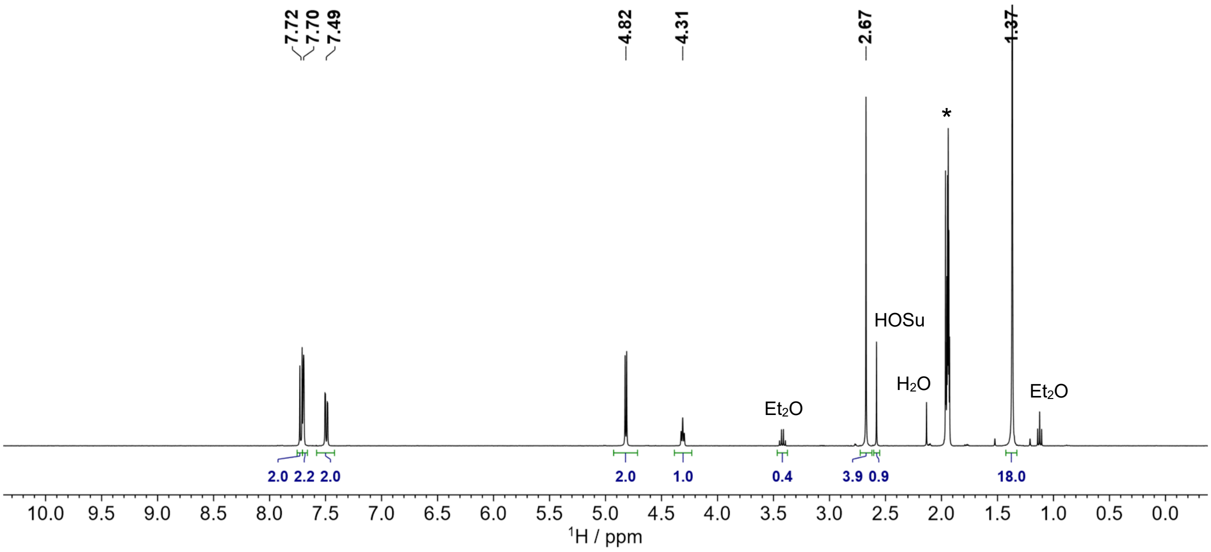

Figure S6 | ^{1}H NMR spectrum (400.1 MHz, 298 K) of **Fmoc*-OSu** in acetonitrile-d_3 (* = residual solvent peak). Residual Et_2O (~0.1 eq.) (δ = 1.12 ppm, 3.42 ppm) and *N*-hydroxysuccinimide (HOSu, ~0.2 eq.) (δ = 2.58 ppm) could not be removed even after prolonged drying *in vacuo*. The NMR solvent contained traces of water (δ = 2.13 ppm).

[Fmoc*-NH-(CH2)6]2

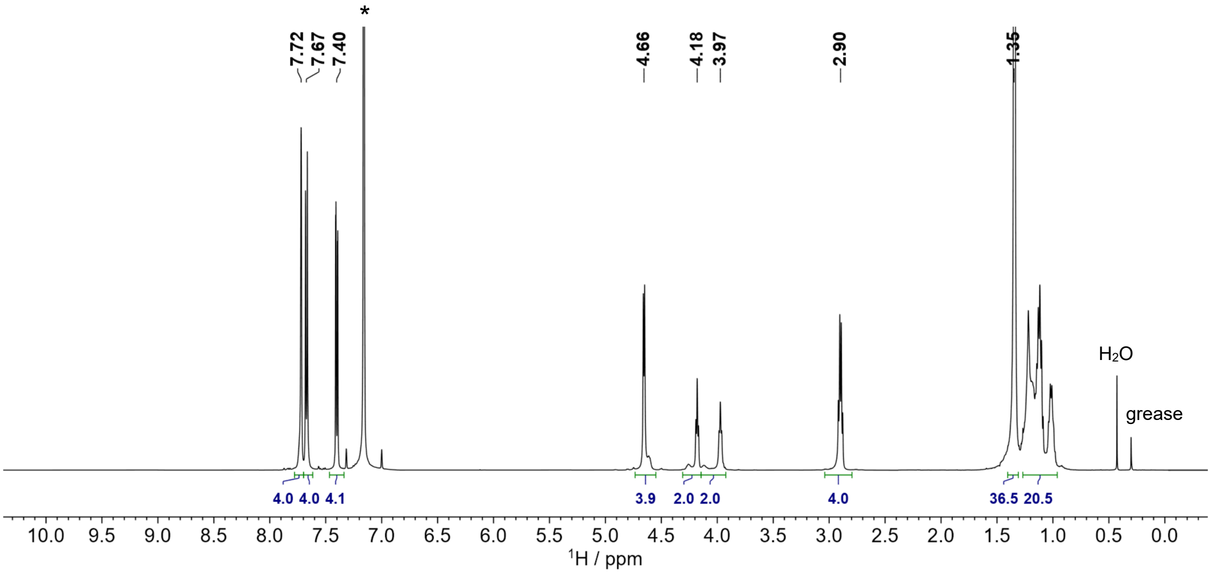

Figure S7 | ^{1}H NMR spectrum (500.1 MHz, 300 K) of **[Fmoc*-NH-(CH2)6]2** in benzene-*d*6 (* = residual solvent peak). The NMR solvent contained traces of water (δ = 0.40 ppm) and silicone grease (δ = 0.29 ppm).

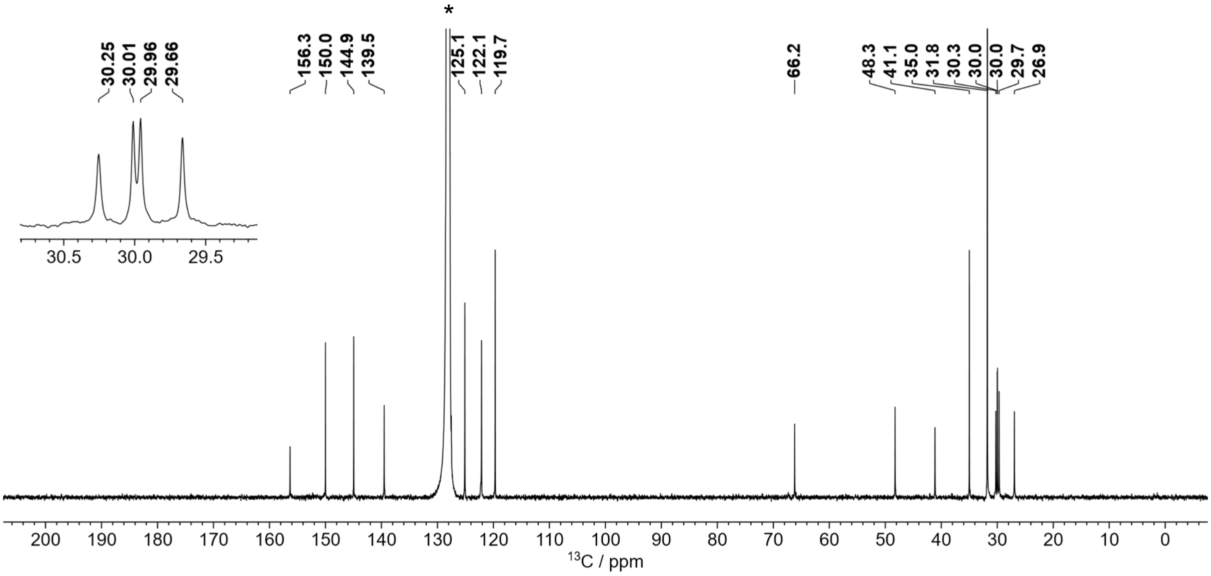

Figure S8 | ^{13}C NMR spectrum (125.8 MHz, 300 K) of **[Fmoc*-NH-(CH2)6]2** in benzene-*d*6 (* = residual solvent peak).

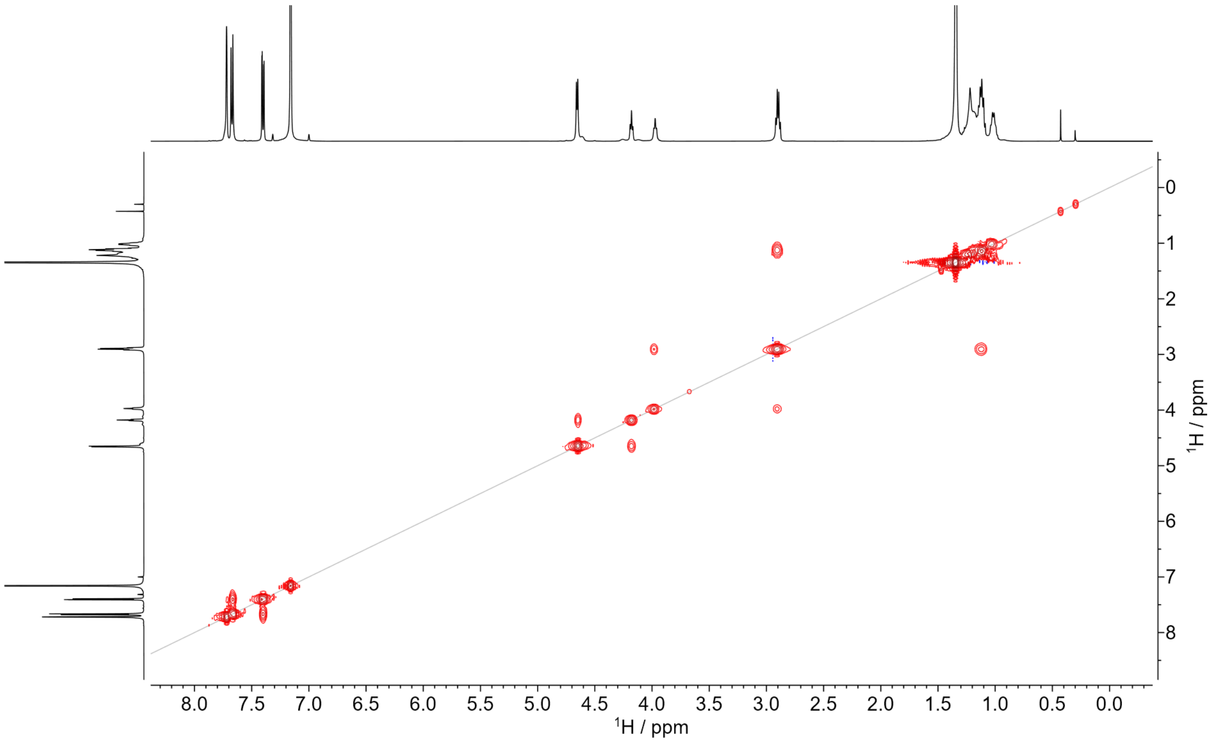

Figure S9 | ^{1}H,^{1}H COSY spectrum (500.1 MHz, 298 K) of **[Fmoc*-NH-(CH$_2$)$_6$]$_2$** in benzene-d_6.

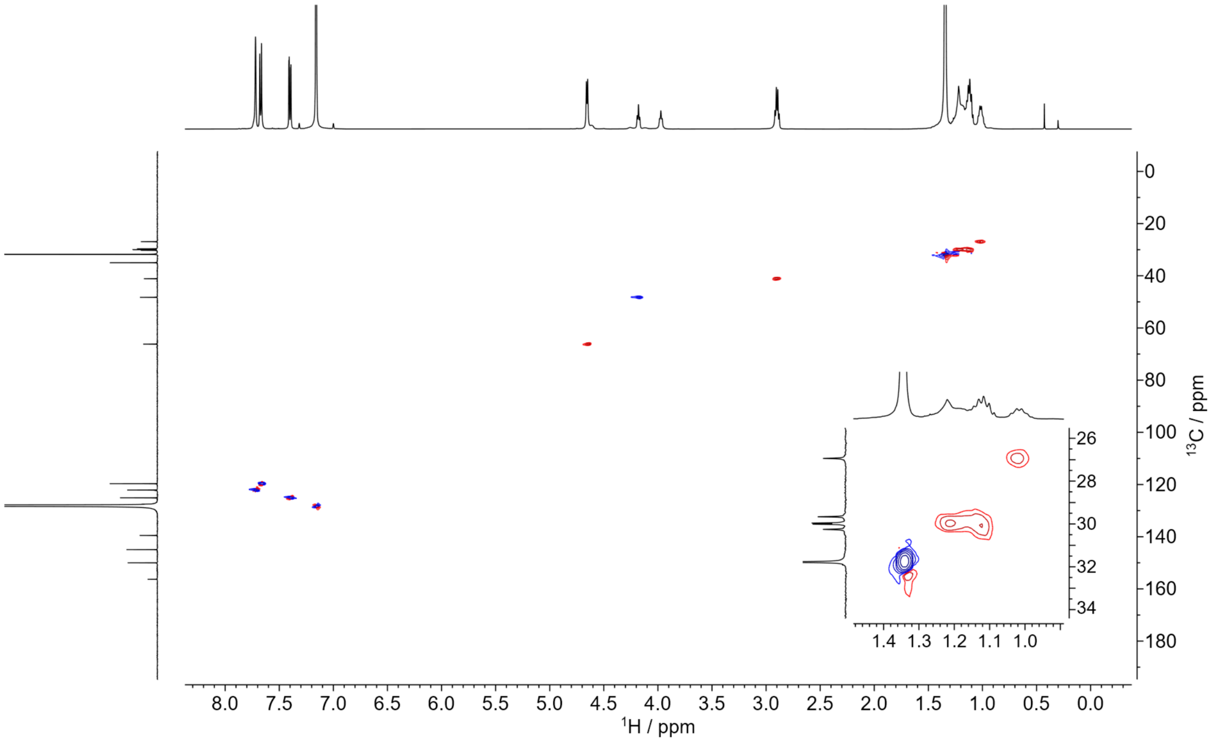

Figure S10 | ^{1}H,^{13}C HSQC spectrum (500.1 MHz, 125.8 MHz, 298 K) of **[Fmoc*-NH-(CH$_2$)$_6$]$_2$** in benzene-d_6.

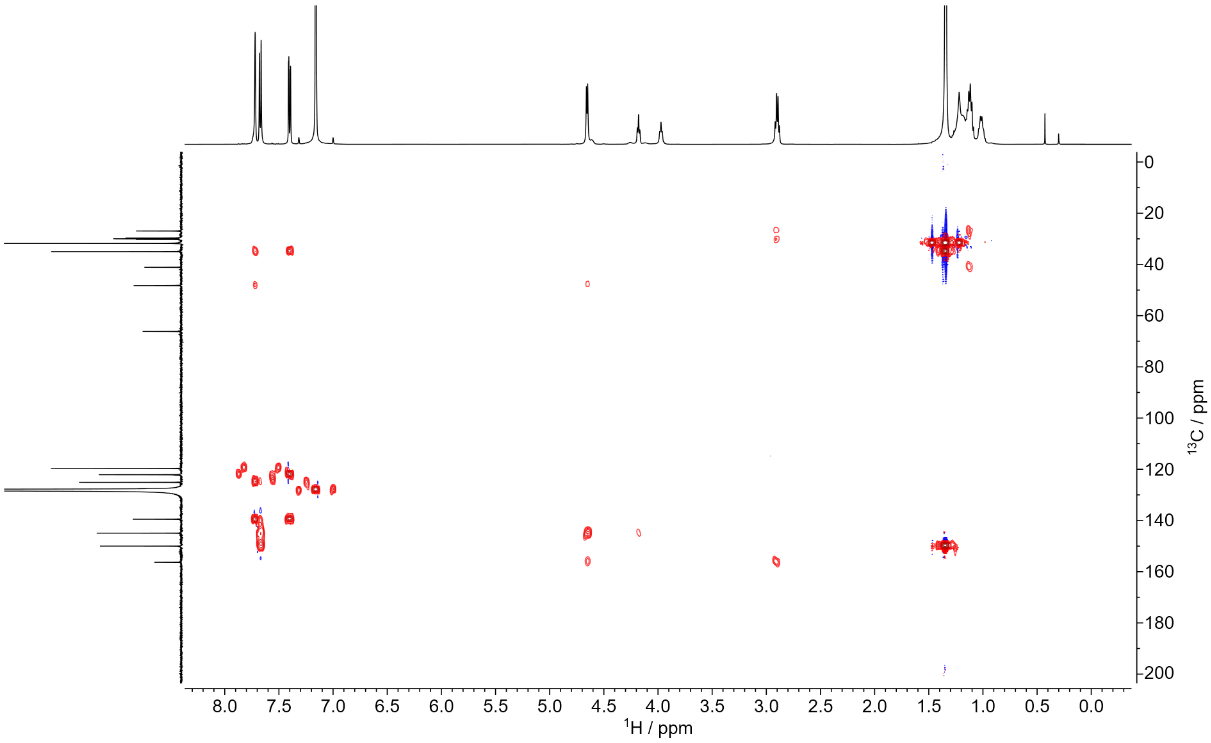

Figure S11 | $^{1}H,^{13}C$ HMBC spectrum (500.1 MHz, 125.8 MHz, 298 K) of **[Fmoc*-NH-(CH$_2$)$_6$]$_2$** in benzene-d_6.

2,7-Di-*tert*-butyl-dibenzofulvene (DBF*)

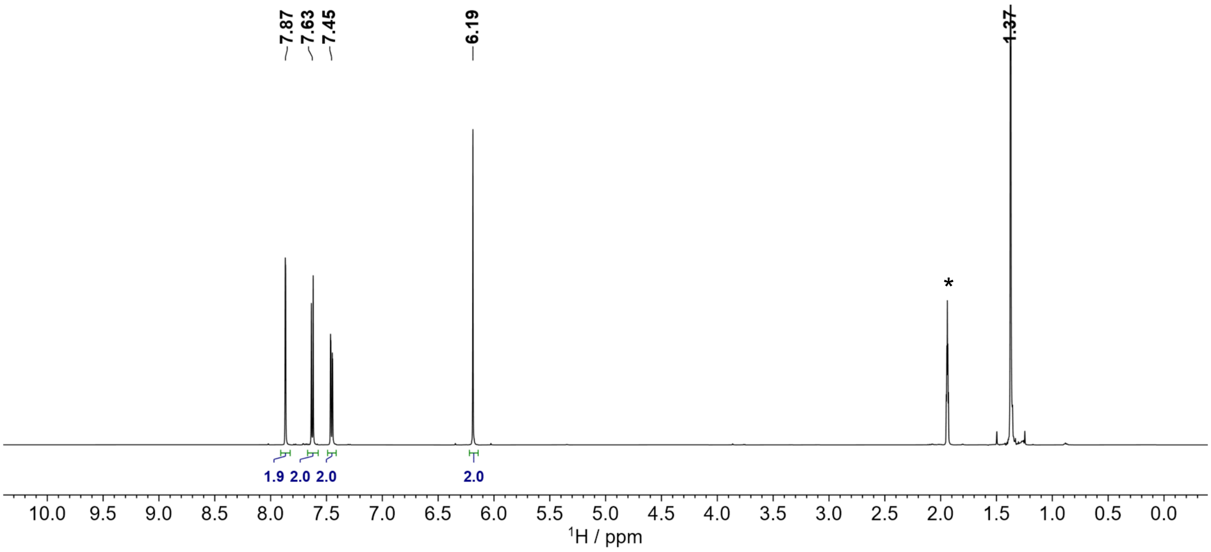

Figure S12 | ^{1}H NMR spectrum (500.1 MHz, 298 K) of **DBF*** in MeCN-*d*3 (* = residual solvent peak).

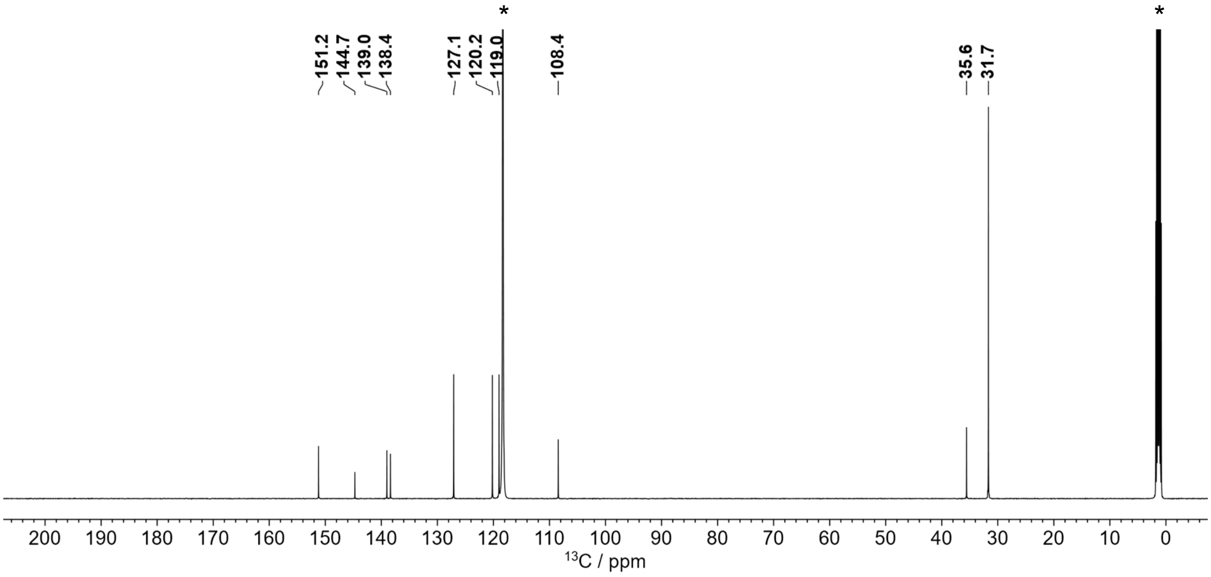

Figure S13 | ^{13}C NMR spectrum (125.8 MHz, 300 K) of **DBF*** in MeCN-*d*3 (* = residual solvent peaks).

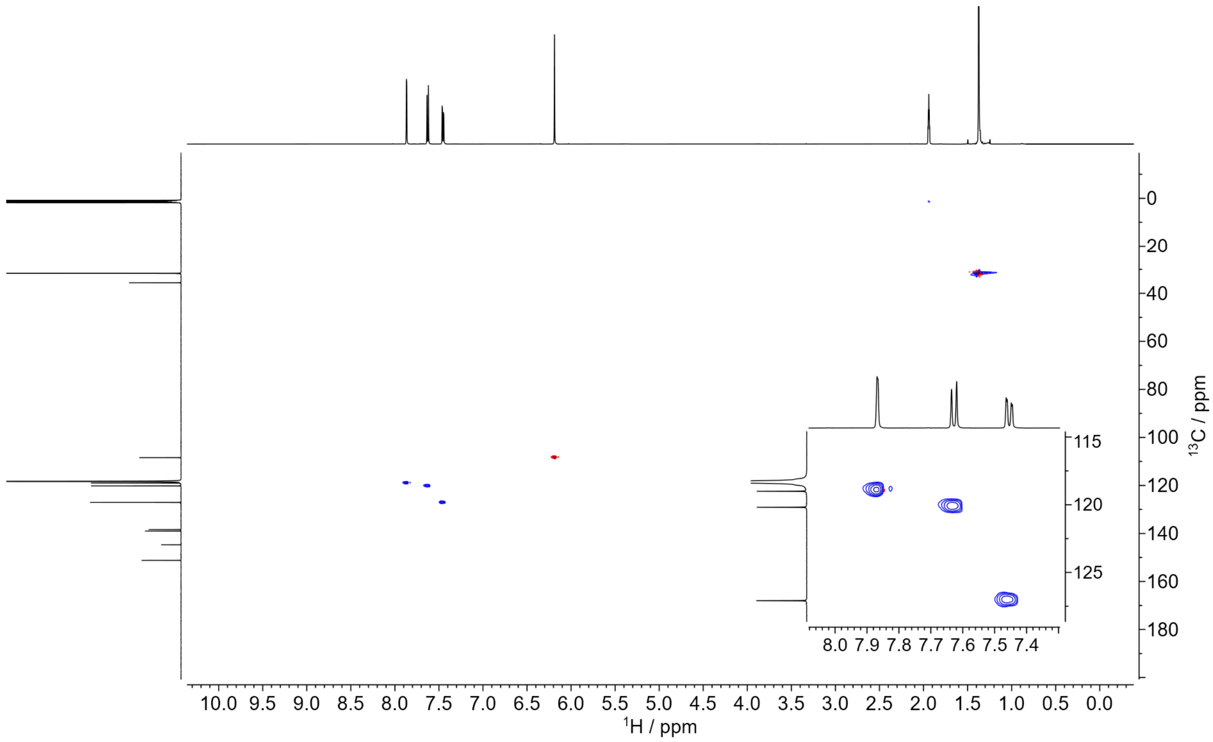

Figure S14 | ^{1}H,^{13}C HSQC spectrum (500.1 MHz, 125.8 MHz, 298 K) of **DBF*** in MeCN-d_3.

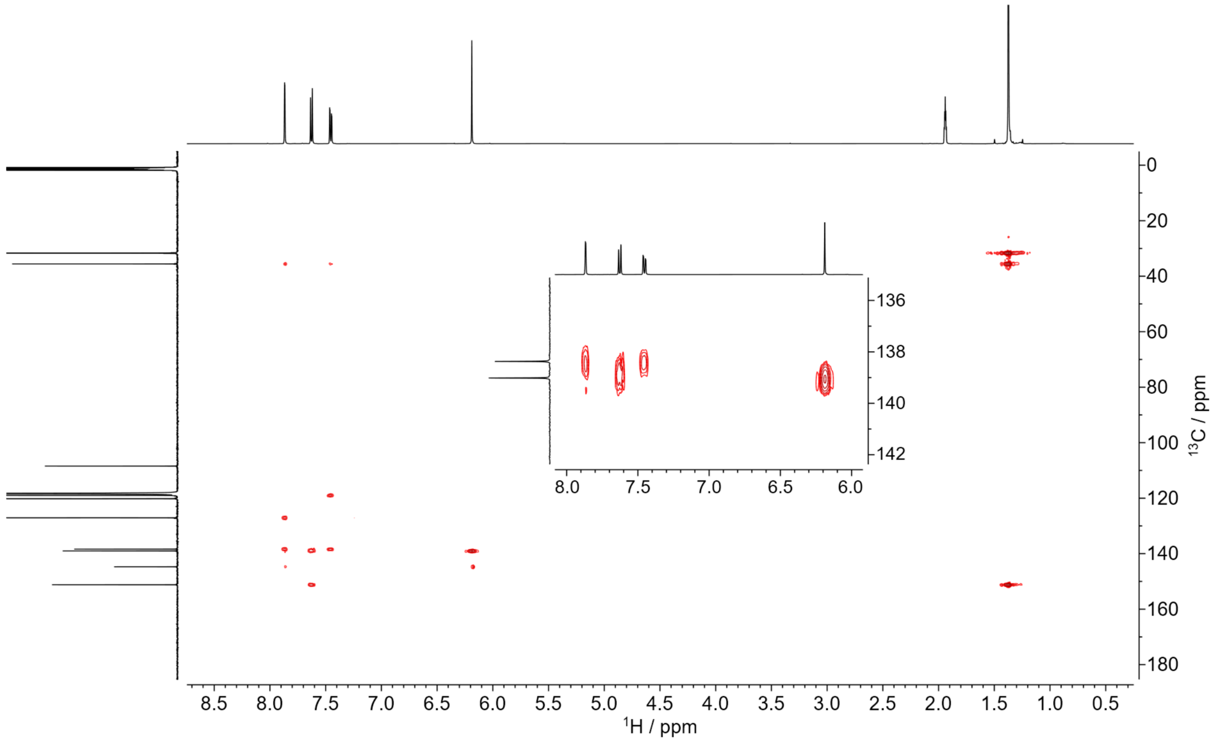

Figure S15 | ^{1}H,^{13}C HMBC spectrum (500.1 MHz, 125.8 MHz, 298 K) of **DBF*** in MeCN-d_3.

[Fmoc*-Rot][Ag$_8$L$_2$](PF$_6$)$_4$

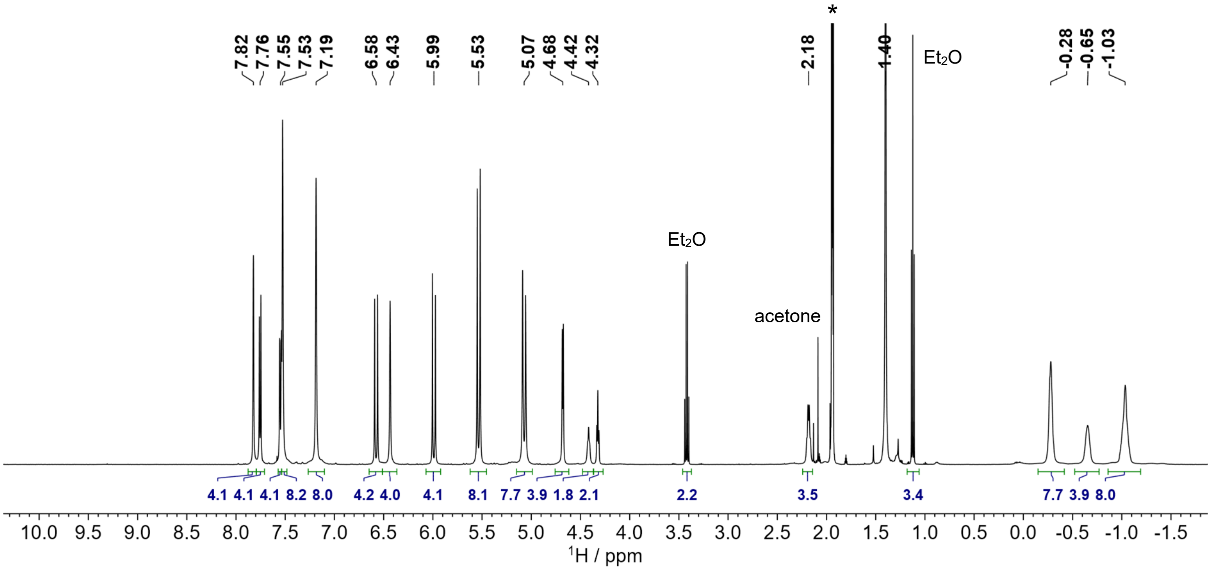

Figure S16 | ^{1}H NMR spectrum (500.1 MHz, 298 K) of **[Fmoc*-Rot][Ag$_8$L$_2$](PF$_6$)$_4$** in MeCN-d_3 (* = residual solvent peak). Residual Et$_2$O (~0.5 eq.) (δ = 1.12 ppm, 3.42 ppm) as well as traces of acetone (δ = 2.08 ppm) could not be removed even after prolonged drying *in vacuo*. The NMR solvent contained traces of water (δ = 2.13 ppm).

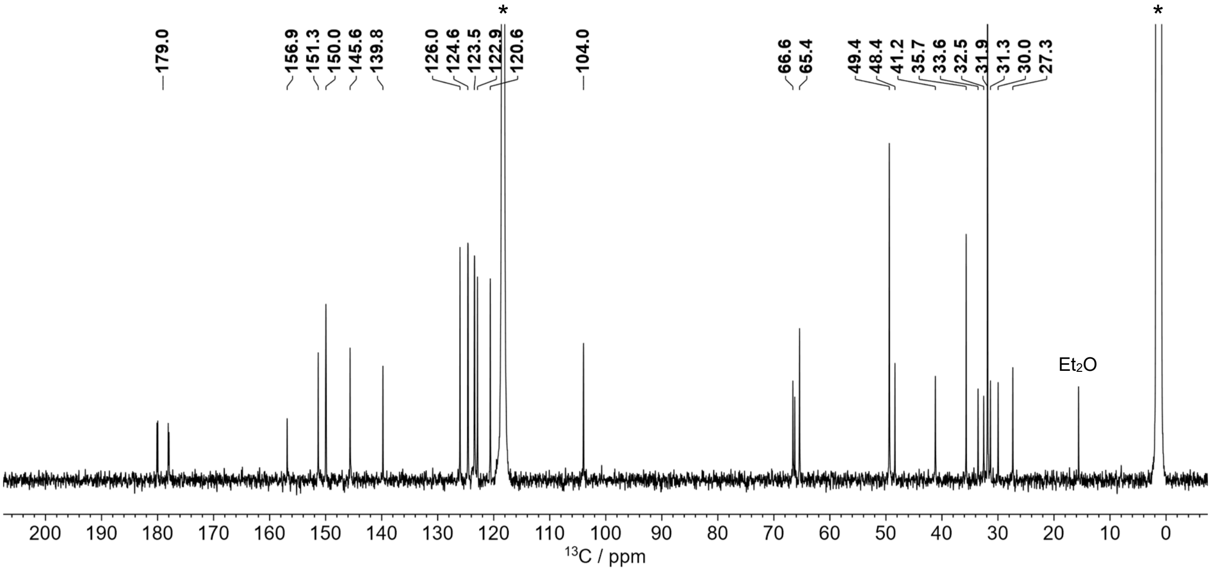

Figure S17 | ^{13}C NMR spectrum (125.8 MHz, 300 K) of **[Fmoc*-Rot][Ag$_8$L$_2$](PF$_6$)$_4$** in MeCN-d_3 (* = residual solvent peaks). Residual Et$_2$O (δ = 15.6 ppm, 66.3 ppm) could not be removed even after prolonged drying *in vacuo*.

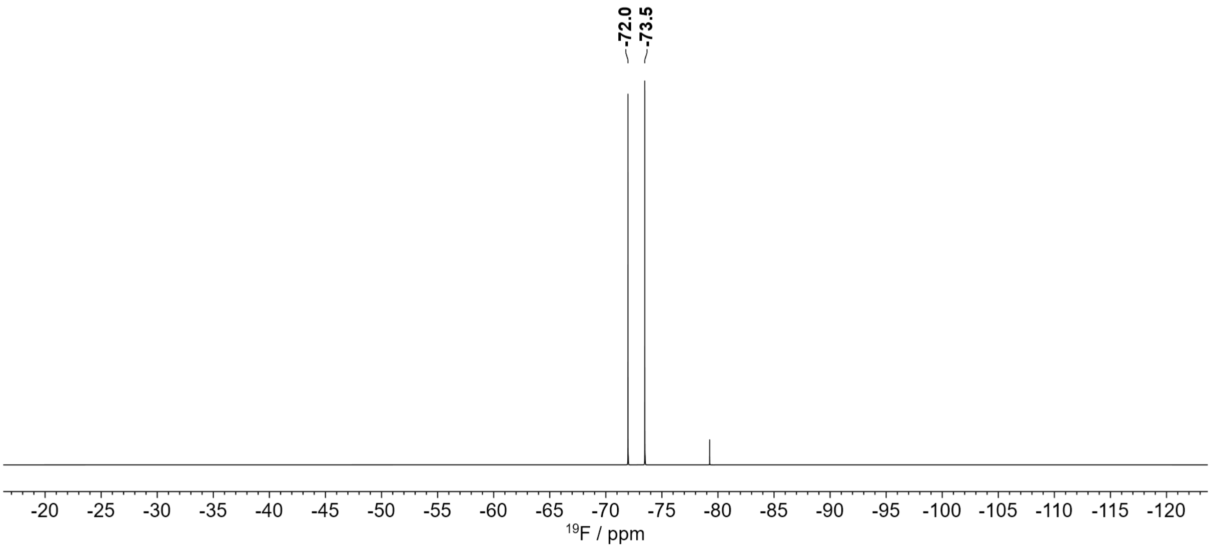

Figure S18 | ^{19}F NMR spectrum (470.8 MHz, 300 K) of **[Fmoc*-Rot][Ag_8L_2](PF_6)$_4$** in MeCN-d_3. Traces of triflate anions (δ = –79.3 ppm, < 7 mol%) originate from the incomplete anion exchange during the synthesis of the ligand precursor **$H_6L(PF_6)_4$**.

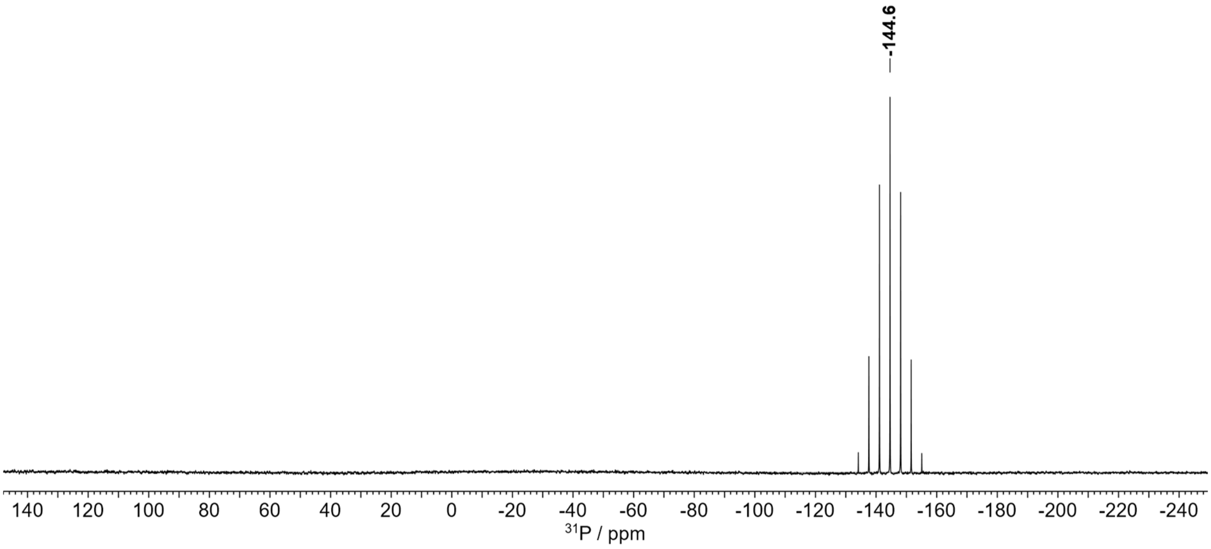

Figure S19 | ^{31}P NMR spectrum (202.6 MHz, 300 K) of **[Fmoc*-Rot][Ag_8L_2](PF_6)$_4$** in MeCN-d_3.

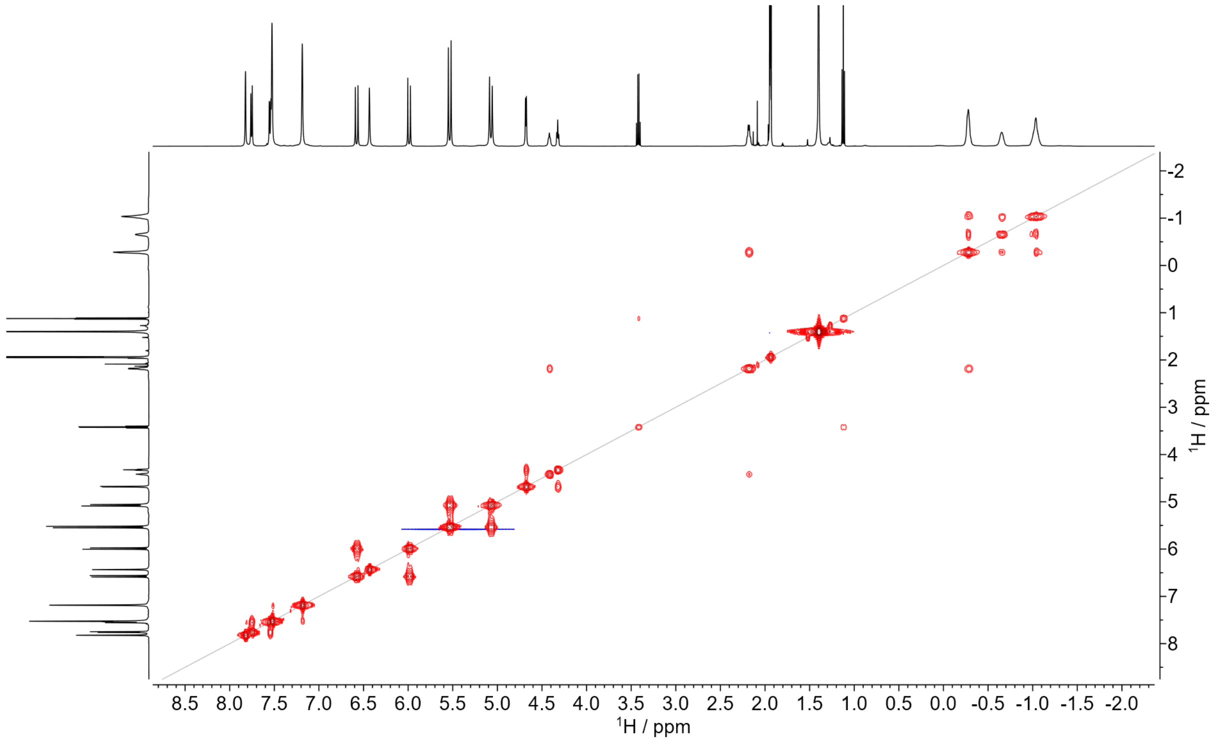

Figure S20 | ^{1}H,^{1}H COSY spectrum (500.1 MHz, 298 K) of **[Fmoc*-Rot][Ag$_8$L$_2$](PF$_6$)$_4$** in MeCN-d_3.

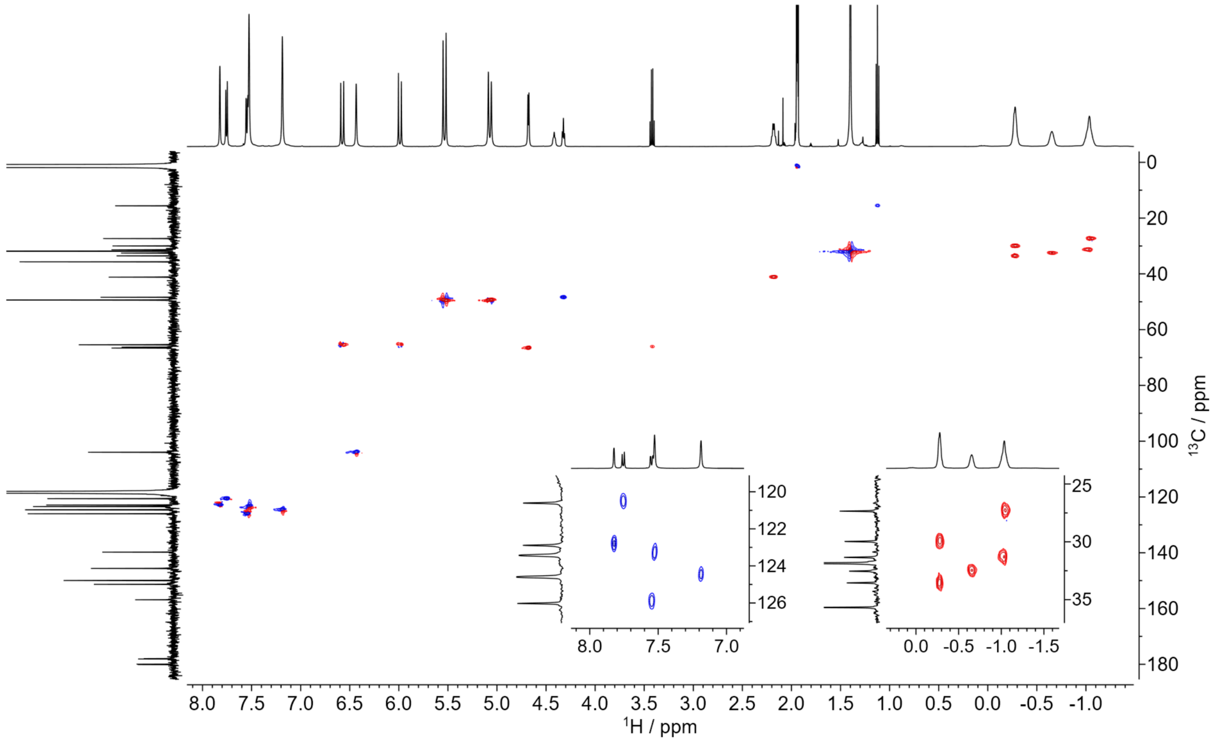

Figure S21 | ^{1}H,^{13}C HSQC spectrum (500.1 MHz, 125.8 MHz, 298 K) of **[Fmoc*-Rot][Ag$_8$L$_2$](PF$_6$)$_4$** in MeCN-d_3.

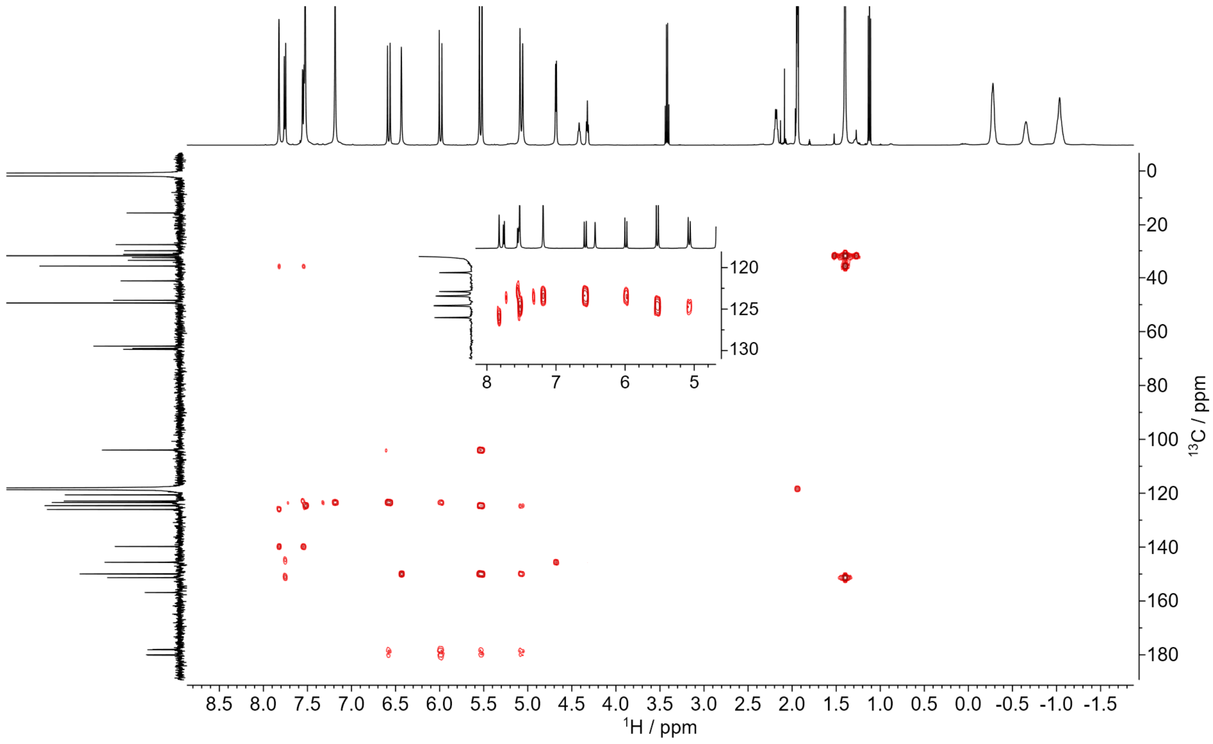

Figure S22 | $^{1}H,^{13}C$ HMBC spectrum (500.1 MHz, 125.8 MHz, 298 K) of **[Fmoc*-Rot][Ag$_8$L$_2$](PF$_6$)$_4$** in MeCN-*d*$_3$.

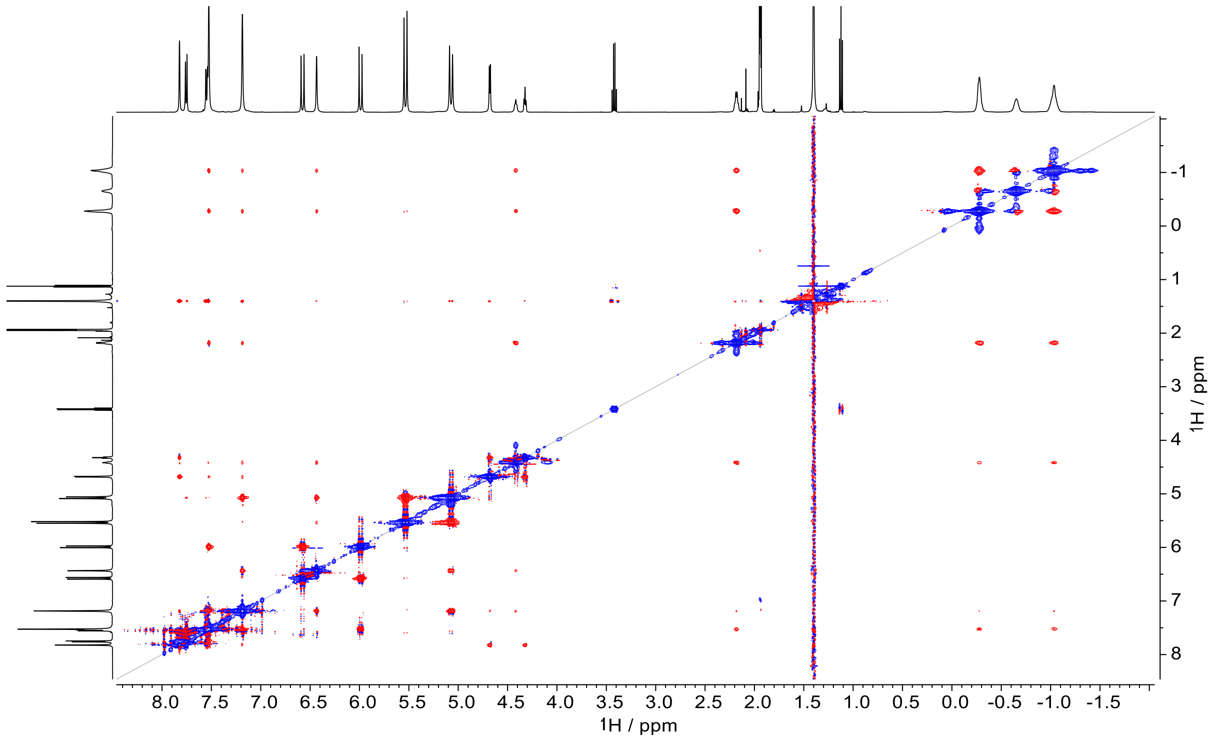

Figure S23 | $^{1}H,^{1}H$ ROESY spectrum (500.1 MHz, 298 K) of **[Fmoc*-Rot][Ag$_8$L$_2$](PF$_6$)$_4$** in MeCN-*d*$_3$ (relaxation delay D1 = 1.9 s, mixing time P15 = 200 ms).

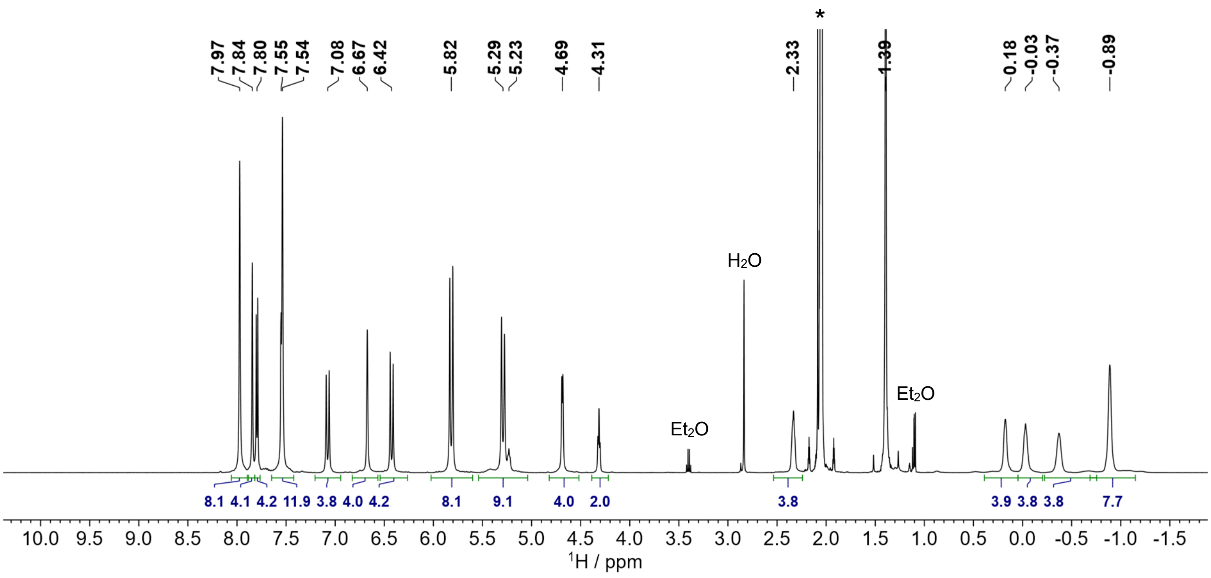

Figure S24 | ^{1}H NMR spectrum (500.1 MHz, 298 K) of **[Fmoc*-Rot][Ag$_8$L$_2$](PF$_6$)$_4$** in acetone-*d*$_6$ (* = residual solvent peak). Traces of Et_2O (~8 mol%) (δ = 1.11 ppm, 3.40 ppm) could not be removed even after prolonged drying *in vacuo*. The NMR solvent contained traces of H_2O (δ = 2.87 ppm) and small amounts of diacetone alcohol (DDA), an aldol reaction product of acetone formed by prolonged storage of the solvent.

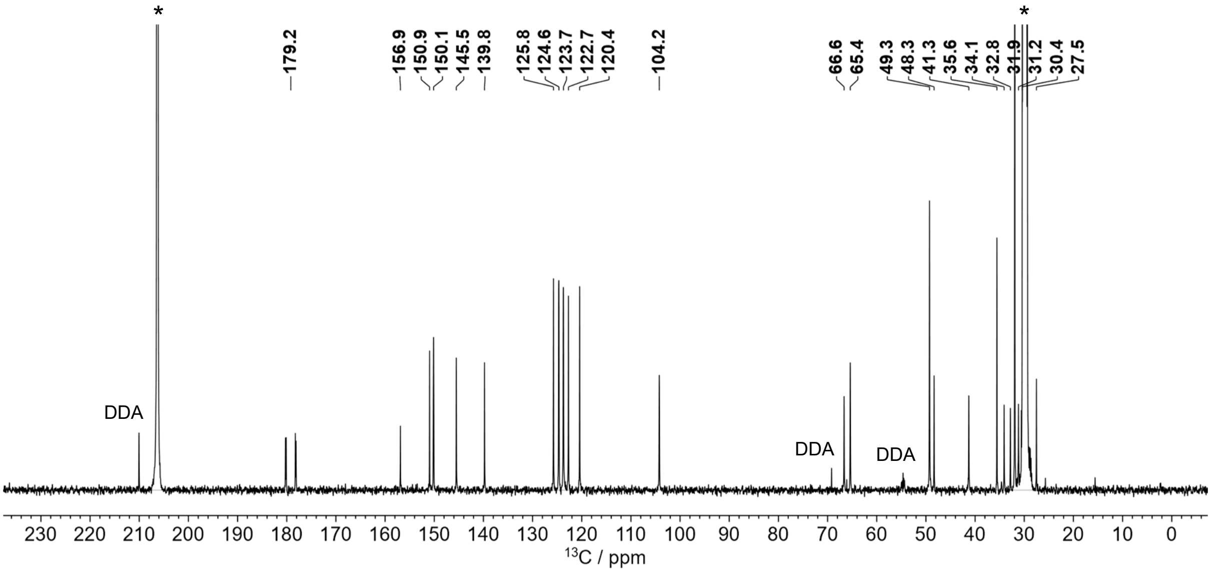

Figure S25 | ^{13}C NMR spectrum (125.8 MHz, 300 K) of **[Fmoc*-Rot][Ag$_8$L$_2$](PF$_6$)$_4$** in acetone-*d*$_6$ (* = residual solvent peaks). The NMR solvent contained traces of small amounts of diacetone alcohol (DDA) (δ = 210.1 ppm, 69.2 ppm, 54.6 ppm), an aldol reaction product of acetone-*d*$_6$ formed by prolonged storage of the solvent.

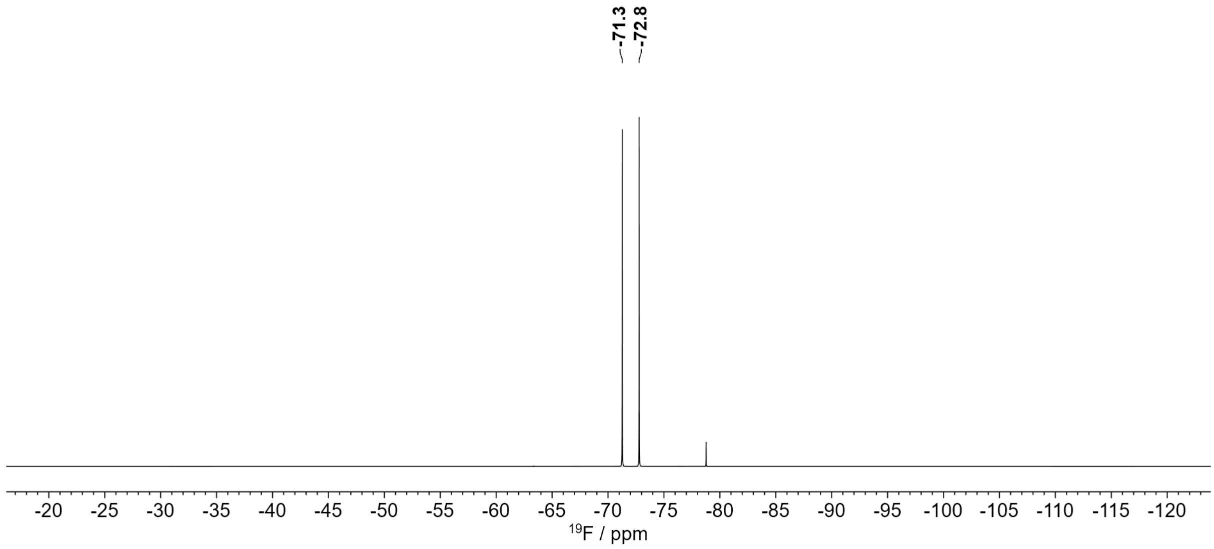

Figure S26 | ^{19}F NMR spectrum (470.8 MHz, 300 K) of **[Fmoc*-Rot][Ag_8L_2](PF_6)$_4$** in acetone-d_6. Traces of triflate anions (δ = –79.3 ppm, < 7 mol%) originate from the incomplete anion exchange during the synthesis of the ligand precursor **$H_6L(PF_6)_4$**.

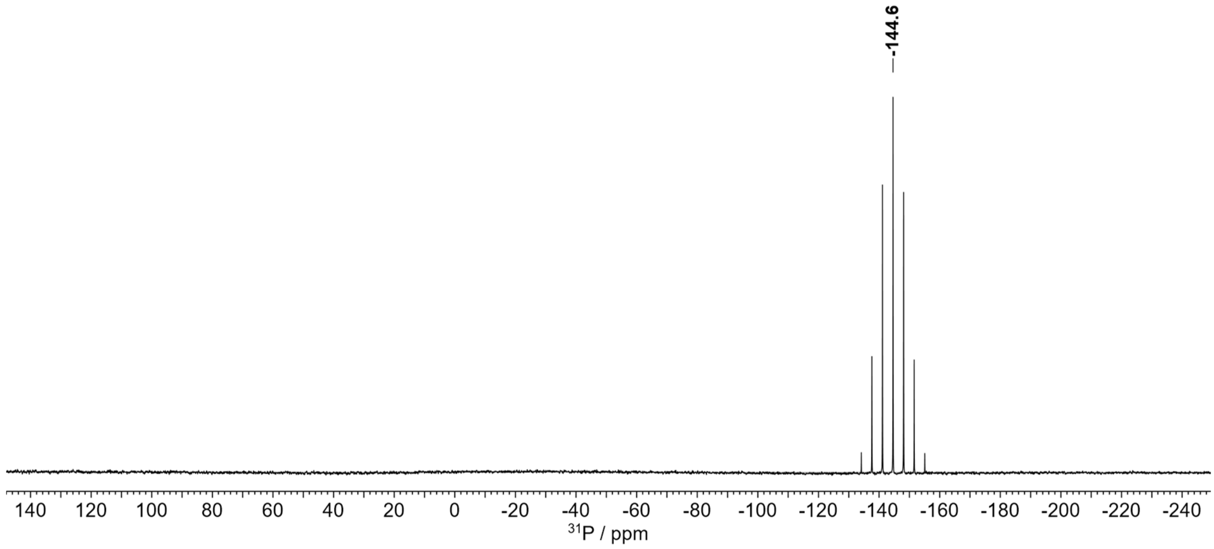

Figure S27 | ^{31}P NMR spectrum (202.6 MHz, 300 K) of **[Fmoc*-Rot][Ag_8L_2](PF_6)$_4$** in acetone-d_6.

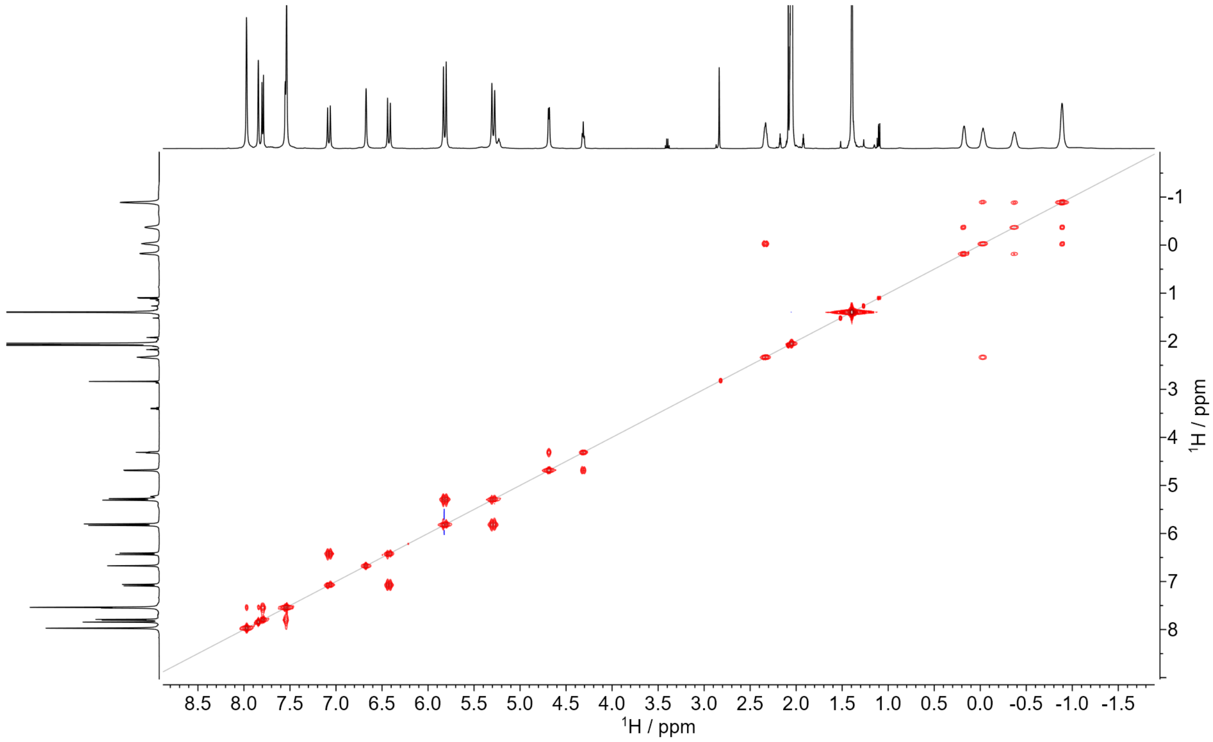

Figure S28 | ^{1}H,^{1}H COSY spectrum (500.1 MHz, 298 K) of **[Fmoc*-Rot][Ag$_8$L$_2$](PF$_6$)$_4$** in acetone-*d*$_6$.

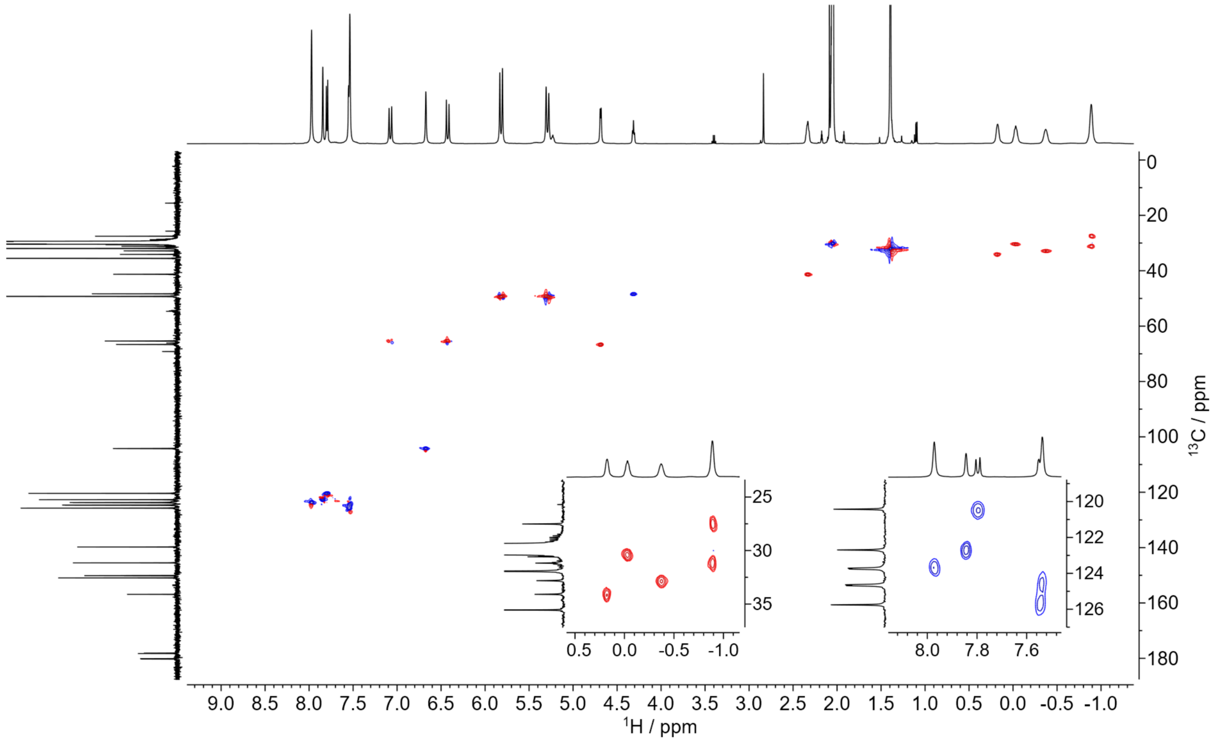

Figure S29 | ^{1}H,^{13}C HSQC spectrum (500.1 MHz, 125.8 MHz, 298 K) of **[Fmoc*-Rot][Ag$_8$L$_2$](PF$_6$)$_4$** in acetone-*d*$_6$.

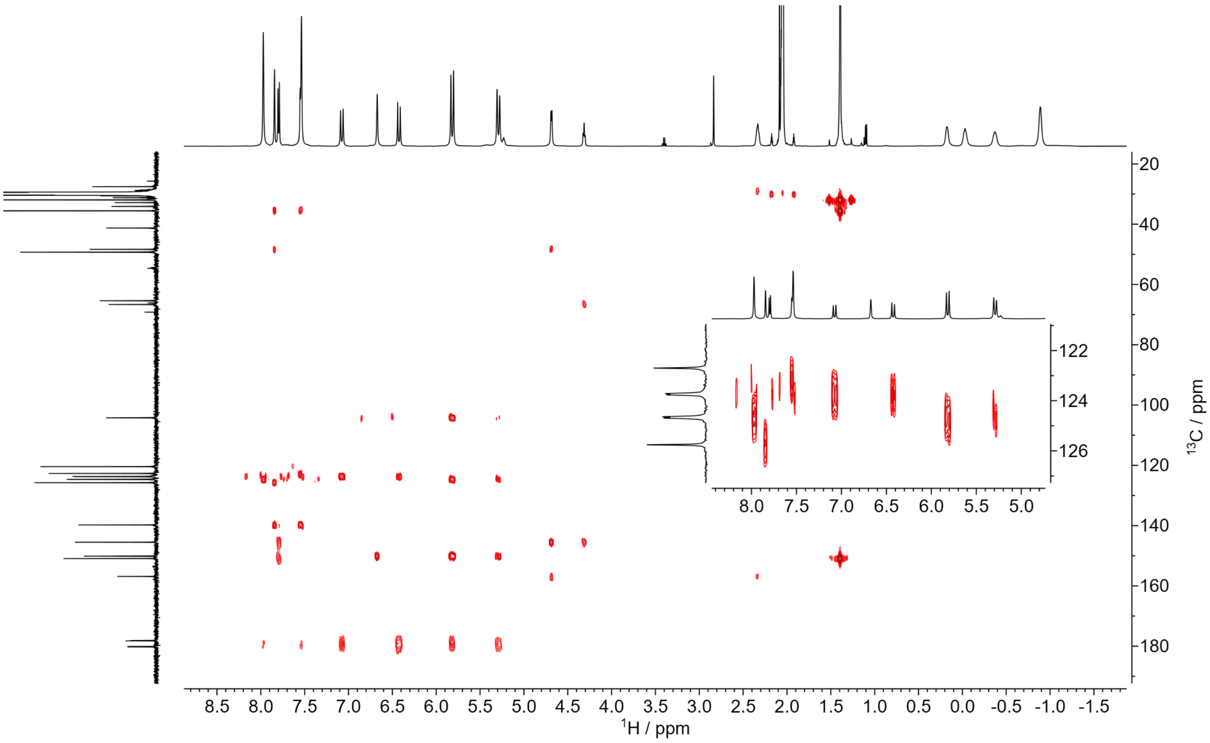

Figure S30 | ^{1}H,^{13}C HMBC spectrum (500.1 MHz, 125.8 MHz, 298 K) of **[Fmoc*-Rot][Ag$_8$L$_2$](PF$_6$)$_4$** in acetone-*d*$_6$.

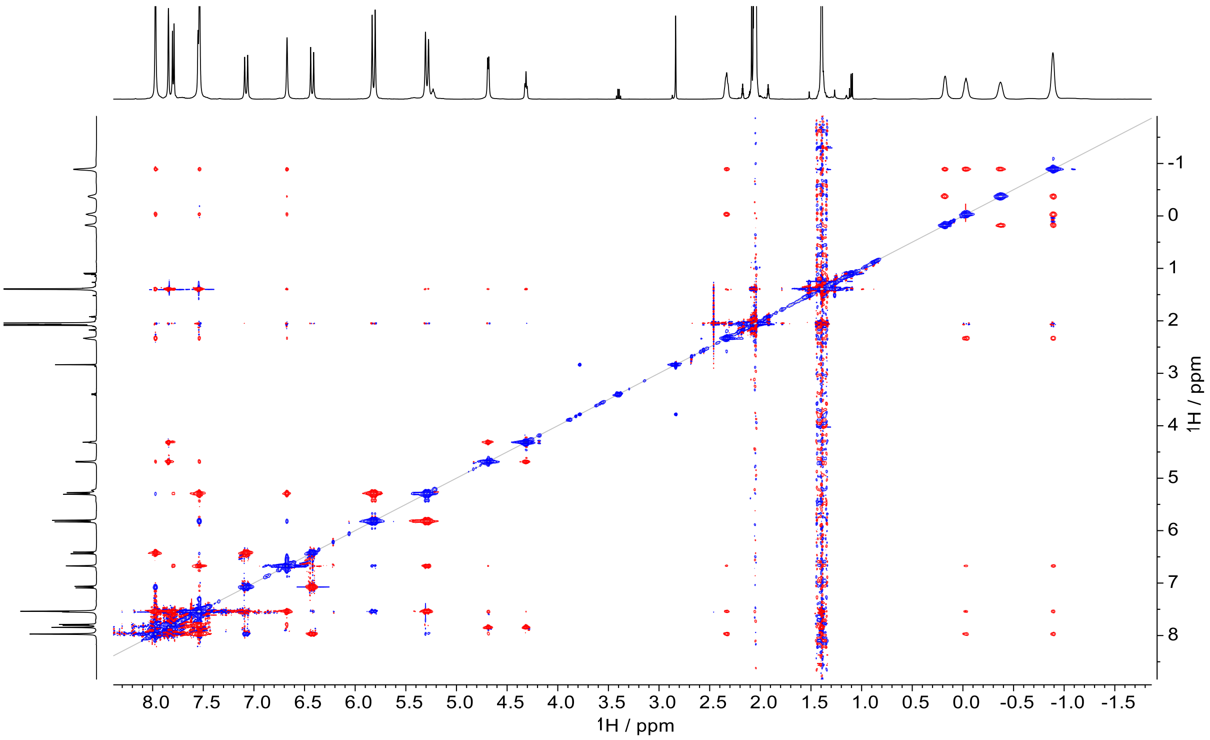

Figure S31 | ^{1}H,^{1}H ROESY spectrum (500.1 MHz, 298 K) of **[Fmoc*-Rot][Ag$_8$L$_2$](PF$_6$)$_4$** in acetone-*d*$_6$ (relaxation delay D1 = 6.0 s, mixing time P15 = 1000 ms).

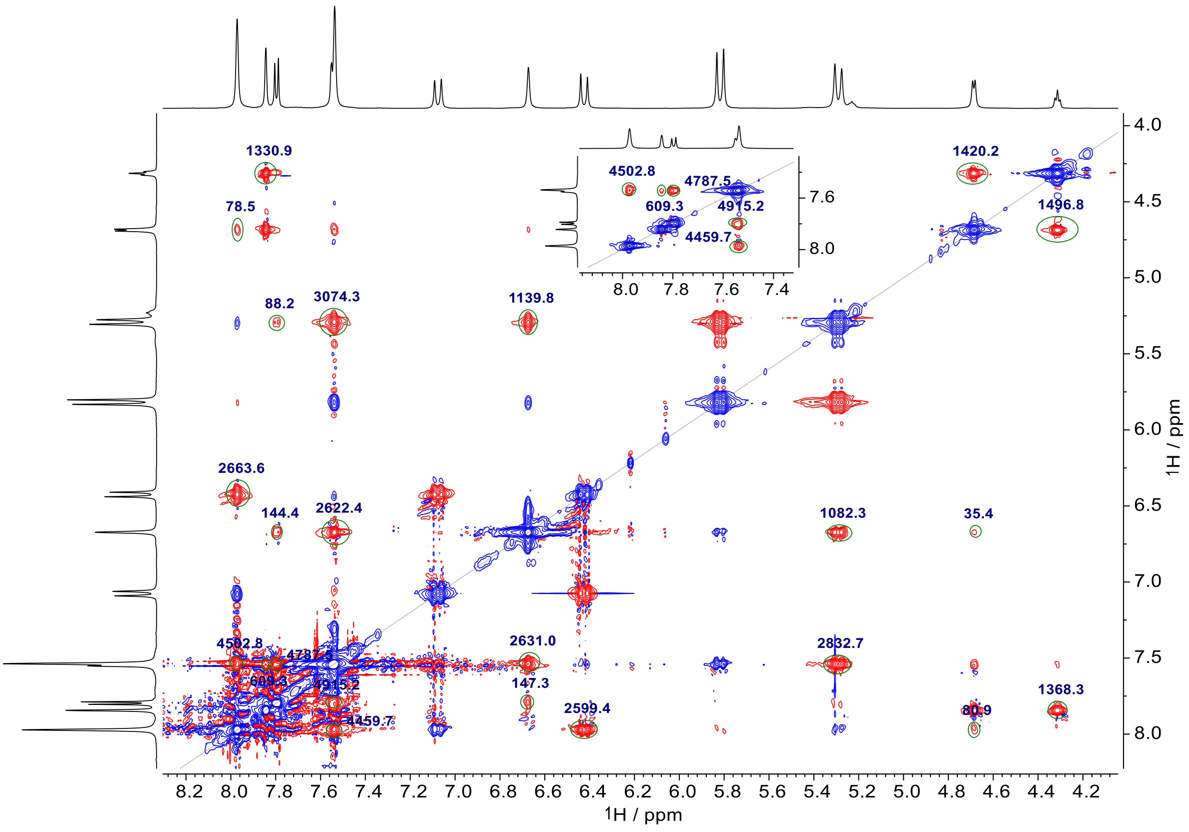

Figure S32 | Cutout of Figure S31, highlighting the cross-peak integrals used for the estimation of interproton distances (*cf.* Section 5).

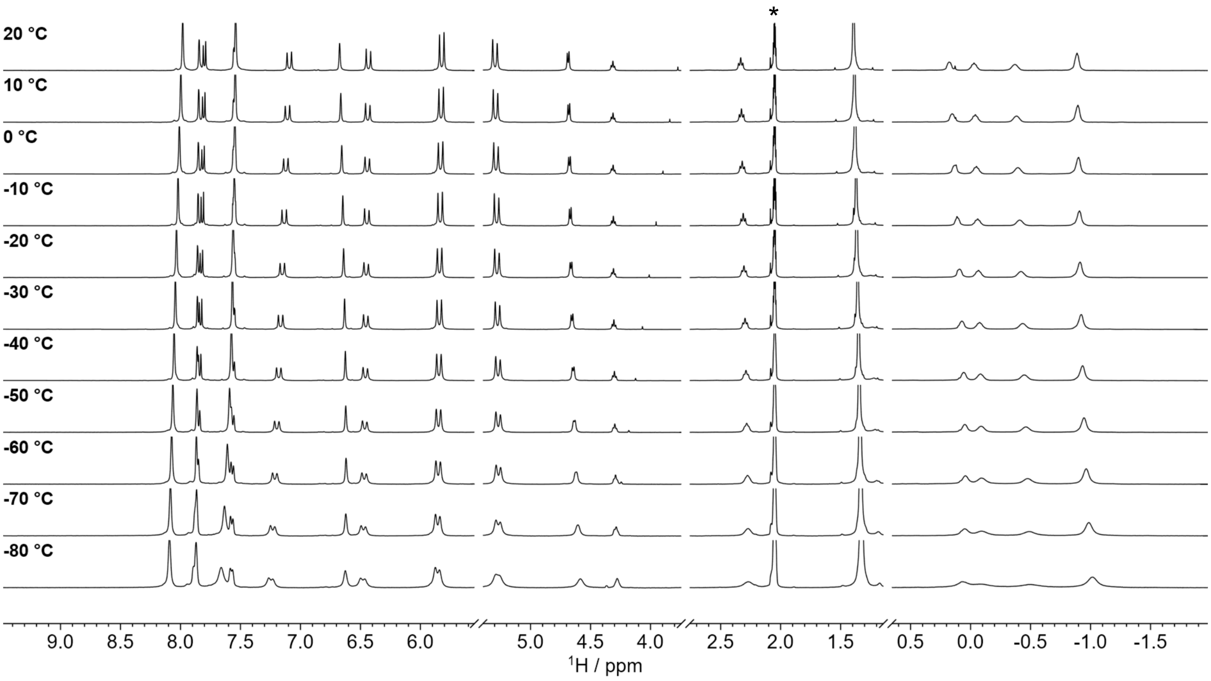

Figure S33 | Temperature-dependent ^{1}H NMR spectra (400.1 MHz) of **[Fmoc*-Rot][Ag$_8$L$_2$](PF$_6$)$_4$** in acetone-d_6 (* = residual solvent peak). Traces of H_2O and Et_2O have been omitted for clarity. Dynamic shuttling of the macrocycle along the axle is expected but too rapid to be resolved even –80 °C. Line broadening observed below –50 °C is attributed either to unavoidably poor shimming or to the presence of slowly interconverting Fmoc* rotamers.

[Amide-Rot][Ag_8L_2](PF_6)$_4$

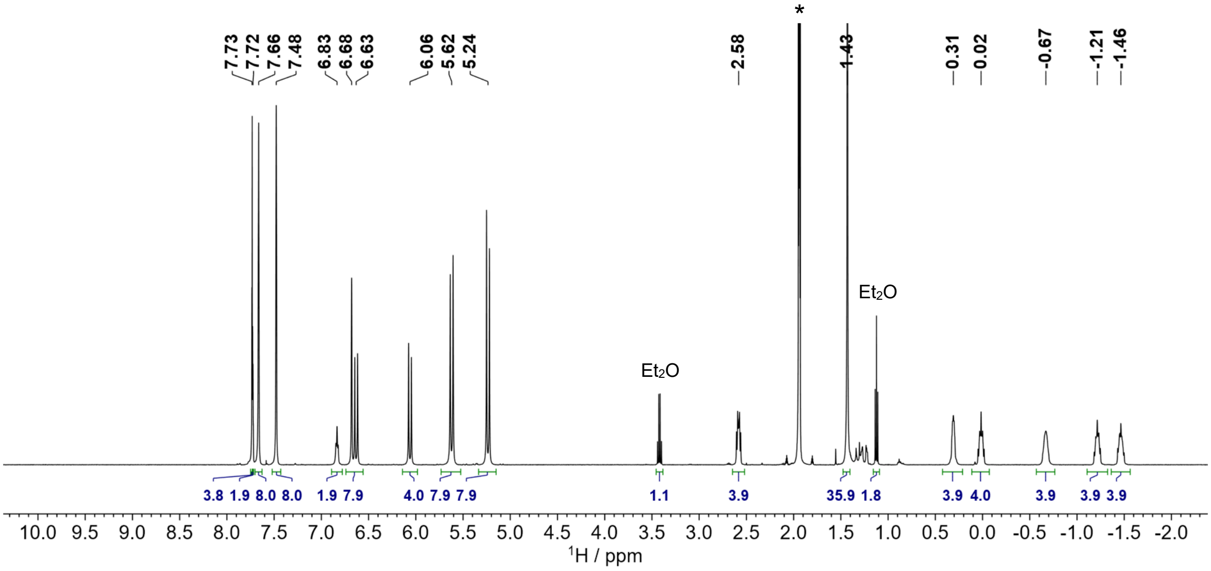

Figure S34 | ^{1}H NMR spectrum (500.1 MHz, 298 K) of **[Amide-Rot][Ag_8L_2](PF_6)$_4$** in MeCN-d_3 (* = residual solvent peak). Residual Et_2O (~0.3 eq.) (δ = 1.12 ppm, 3.42 ppm) could not be removed even after prolonged drying *in vacuo*. The NMR solvent contained traces of grease (δ = 0.86 ppm, 1.27 ppm).

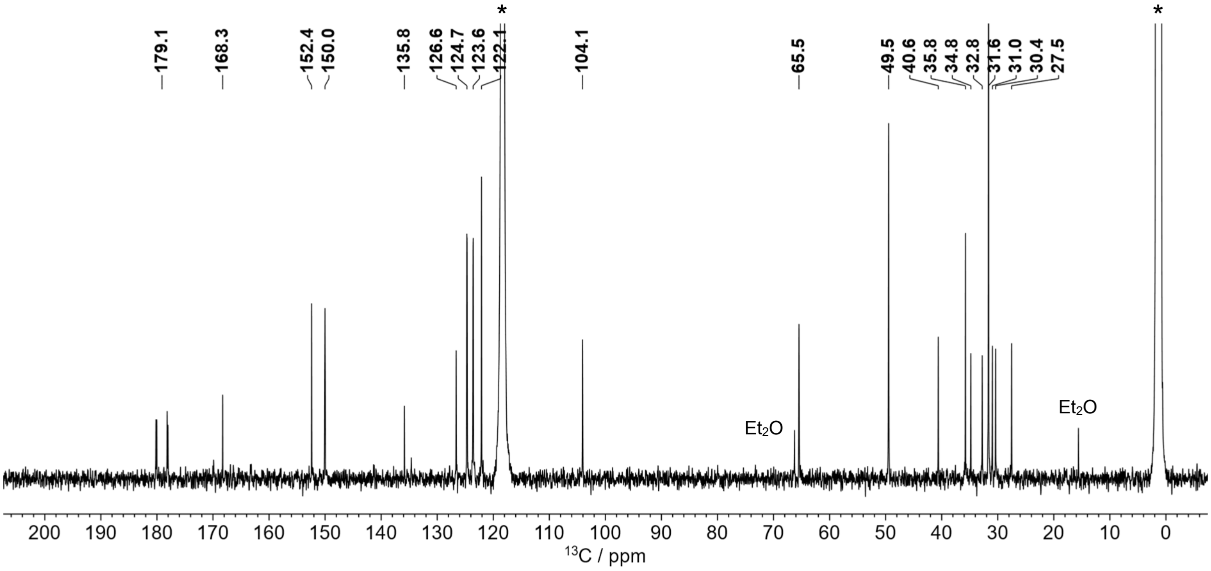

Figure S35 | ^{13}C NMR spectrum (125.8 MHz, 300 K) of **[Amide-Rot][Ag_8L_2](PF_6)$_4$** in MeCN-d_3 (* = residual solvent peaks). Residual Et_2O (δ = 15.6 ppm, 66.3 ppm) could not be removed even after prolonged drying *in vacuo*.

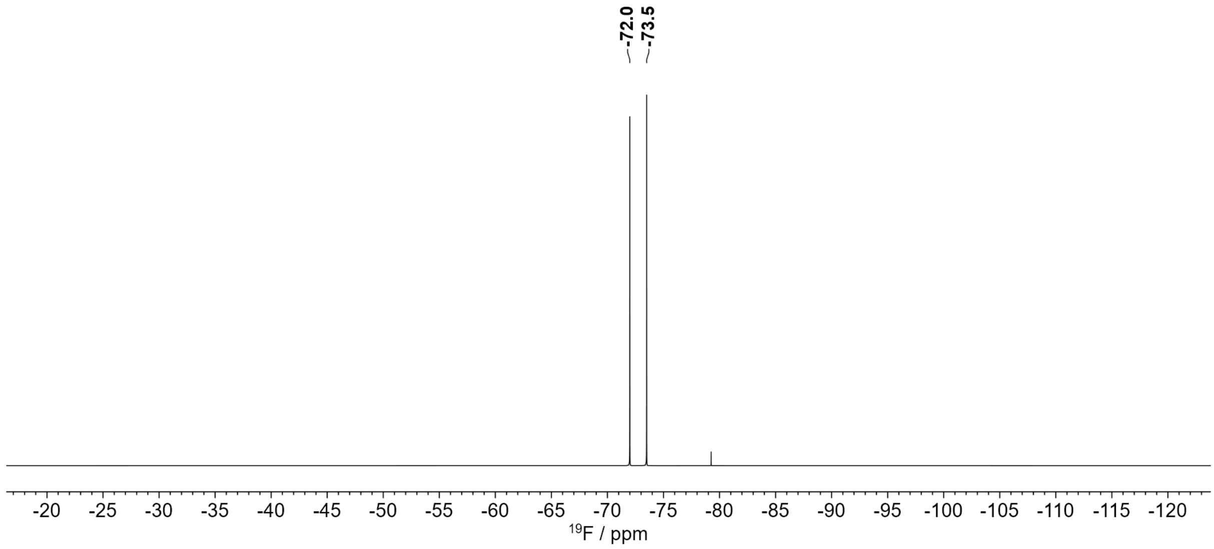

Figure S36 | ^{19}F NMR spectrum (470.8 MHz, 300 K) of **[Amide-Rot][Ag_8L_2](PF_6)$_4$** in MeCN-d_3. Traces of triflate anions (δ = –79.3 ppm, < 5 mol%) originate from the incomplete anion exchange during the synthesis of the ligand precursor **$H_6L(PF_6)_4$**.

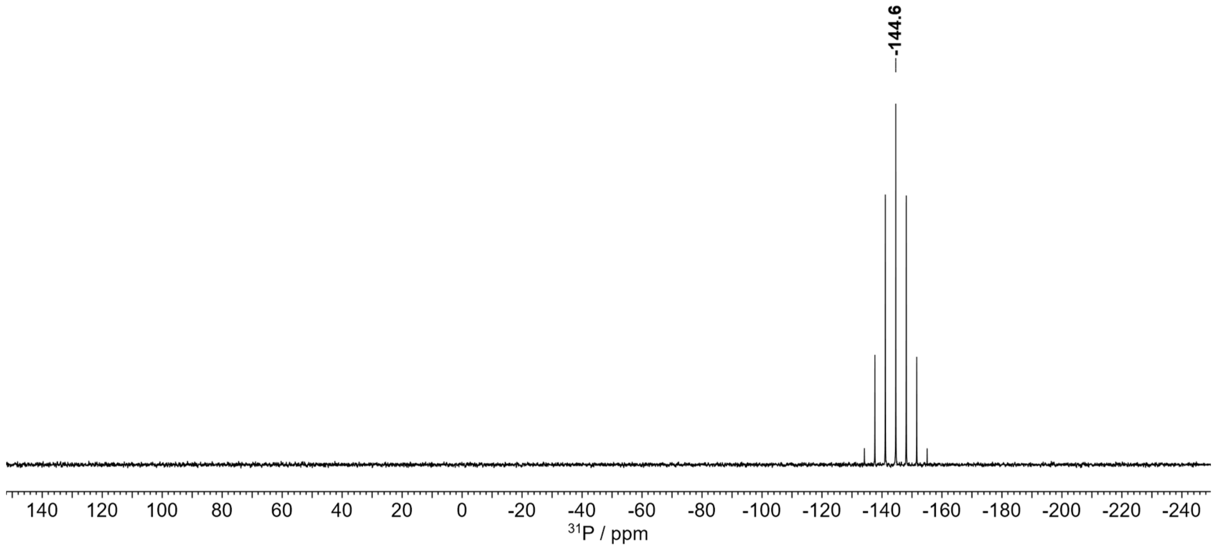

Figure S37 | ^{31}P NMR spectrum (202.6 MHz, 300 K) of **[Amide-Rot][Ag_8L_2](PF_6)$_4$** in MeCN-d_3.

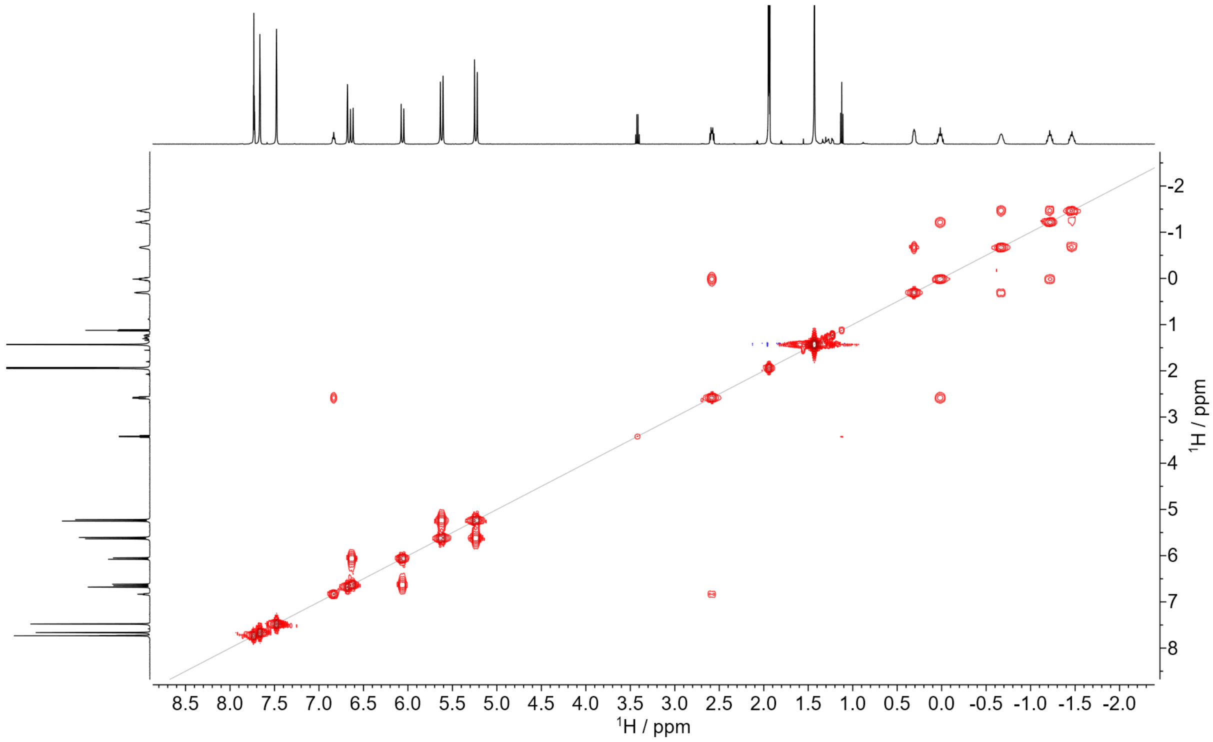

Figure S38 | ^{1}H,^{1}H COSY spectrum (500.1 MHz, 298 K) of **[Amide-Rot][Ag$_8$L$_2$](PF$_6$)$_4$** in MeCN-d_3.

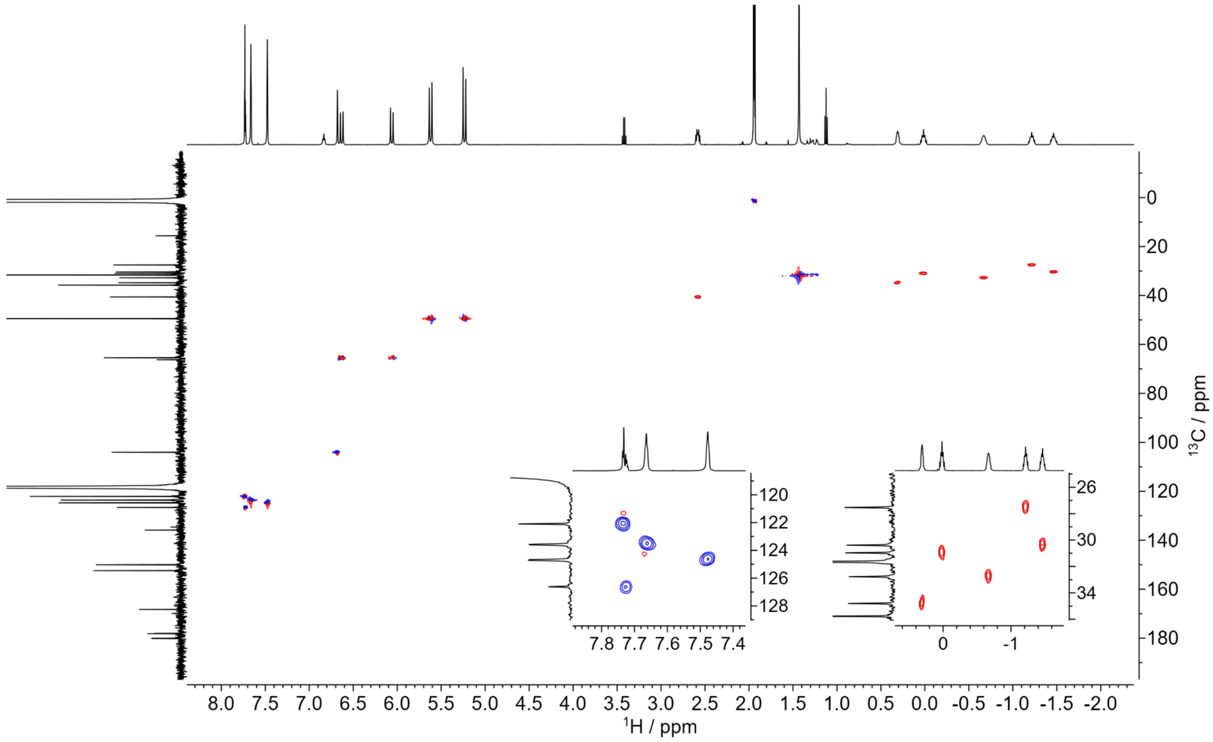

Figure S39 | ^{1}H,^{13}C HSQC spectrum (500.1 MHz, 125.8 MHz, 298 K) of **[Amide-Rot][Ag$_8$L$_2$](PF$_6$)$_4$** in MeCN-d_3.

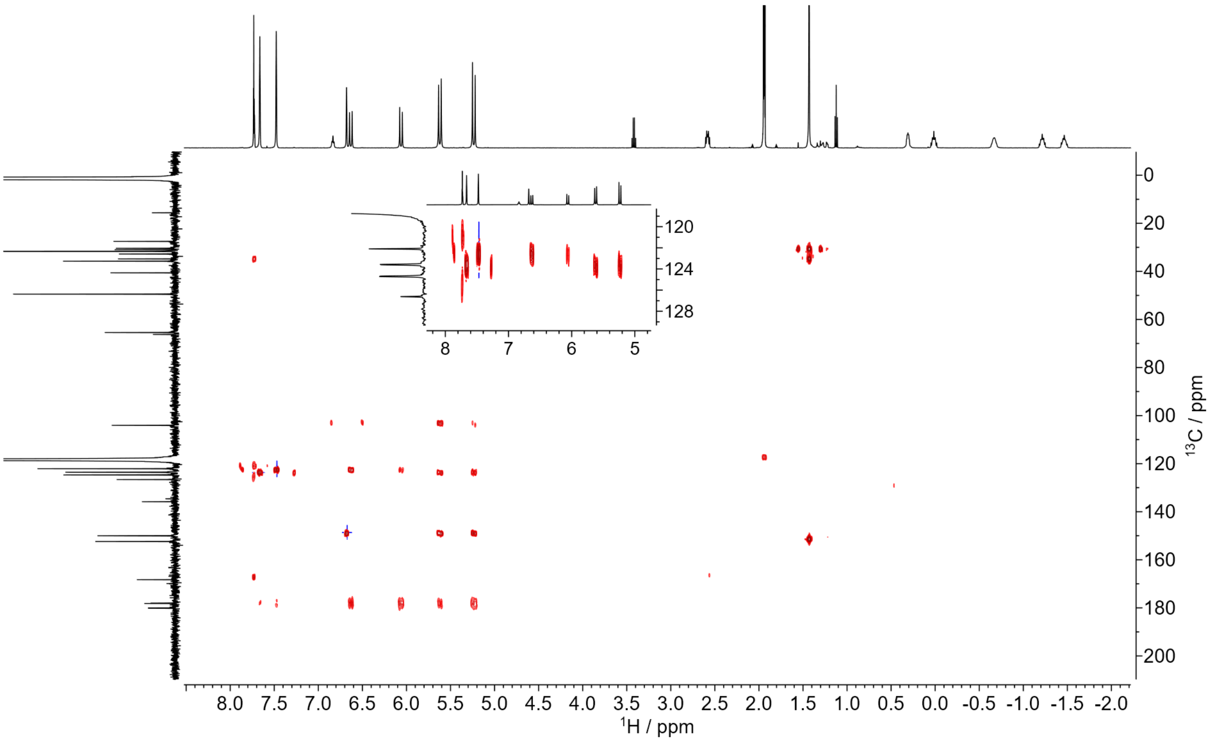

Figure S40 | $^{1}H,^{13}C$ HMBC spectrum (500.1 MHz, 125.8 MHz, 298 K) of **[Amide-Rot][Ag$_8$L$_2$](PF$_6$)$_4$** in MeCN-d_3.

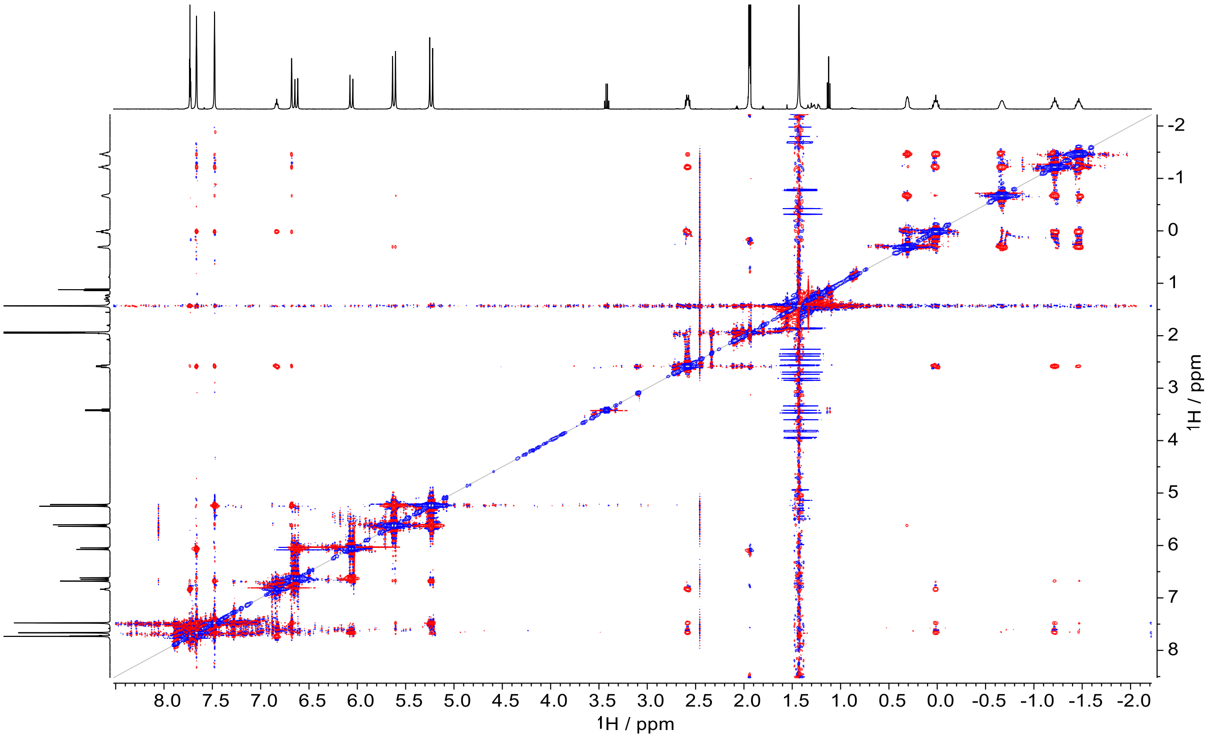

Figure S41 | $^{1}H,^{1}H$ ROESY spectrum (500.1 MHz, 298 K) of **[Amide-Rot][Ag$_8$L$_2$](PF$_6$)$_4$** in MeCN-d_3 (relaxation delay D1 = 1.9 s, mixing time P15 = 200 ms).

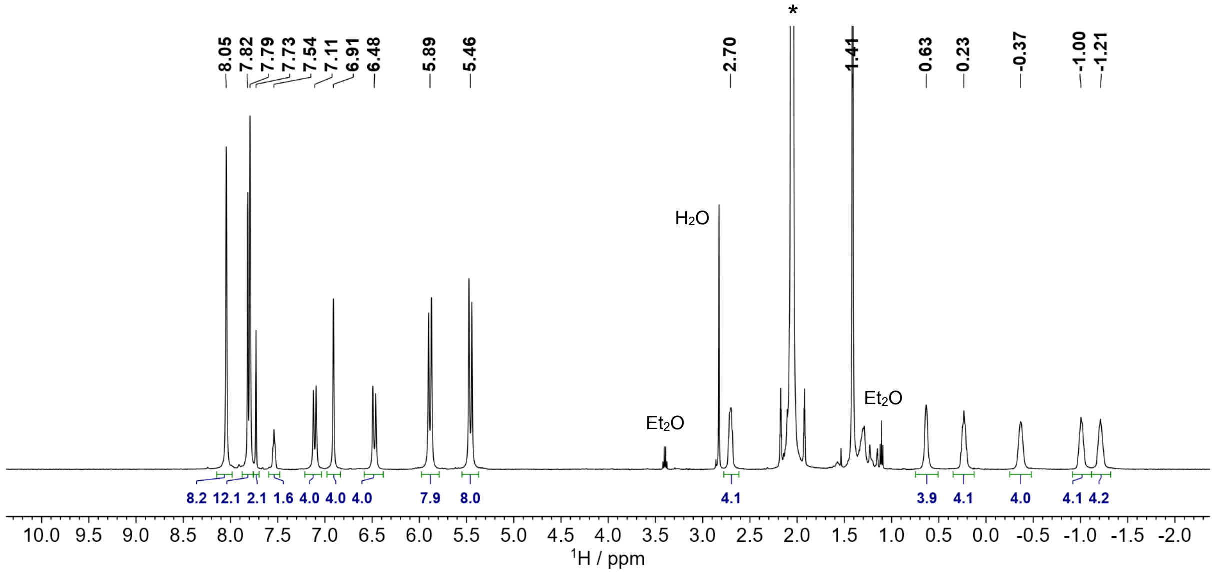

Figure S42 | ^{1}H NMR spectrum (500.1 MHz, 298 K) of **[Amide-Rot][Ag$_8$L$_2$](PF$_6$)$_4$** in acetone-*d*$_6$ (* = residual solvent peak). Residual Et$_2$O (< 0.2 eq.) (δ = 1.10 ppm, 3.42 ppm) could not be removed even after prolonged drying *in vacuo*. The NMR solvent contained traces of H_2O (δ = 2.87 ppm) and small amounts of diacetone alcohol (DDA), an aldol reaction product of acetone-*d*$_6$ formed by prolonged storage of the solvent.

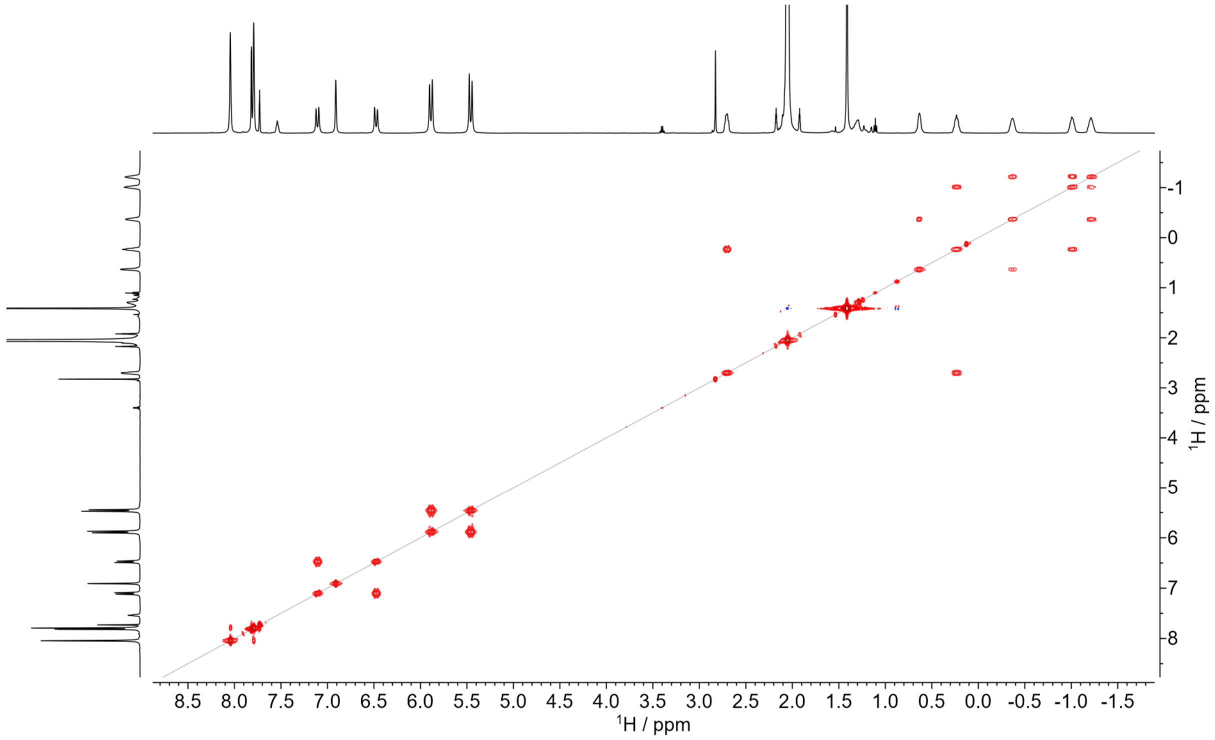

Figure S43 | ^{1}H,^{1}H COSY spectrum (500.1 MHz, 298 K) of **[Amide-Rot][Ag$_8$L$_2$](PF$_6$)$_4$** in acetone-*d*$_6$.

$[Ag_8L_2](PF_6)_4$

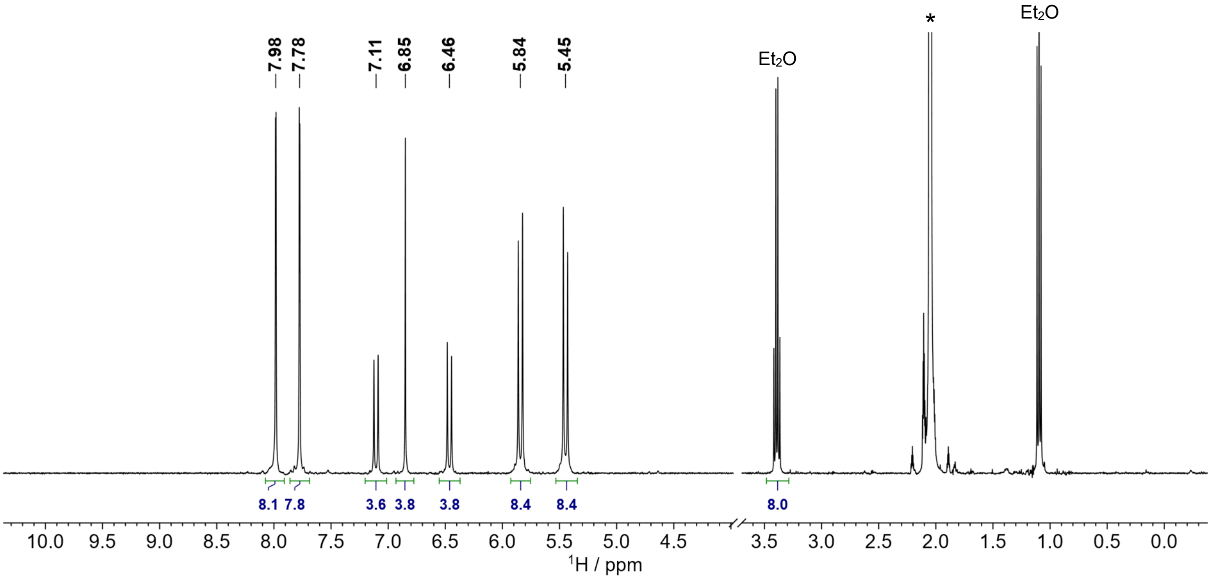

Figure S44 | ^{1}H NMR spectrum (400.1 MHz, 295 K) of **$[Ag_8L_2](PF_6)_4$** in acetone-d_6 (* = residual solvent peak). The sample contained some residual Et_2O (~1.0 eq.) (δ = 1.10 ppm, 3.42 ppm). A peak at 3.79 ppm, presumably originating from water or decomposition products of acetone-d_6 was cut out for clarity.

Comparison of ^{1}H NMR Chemical Shifts of Different Pillarplex Species

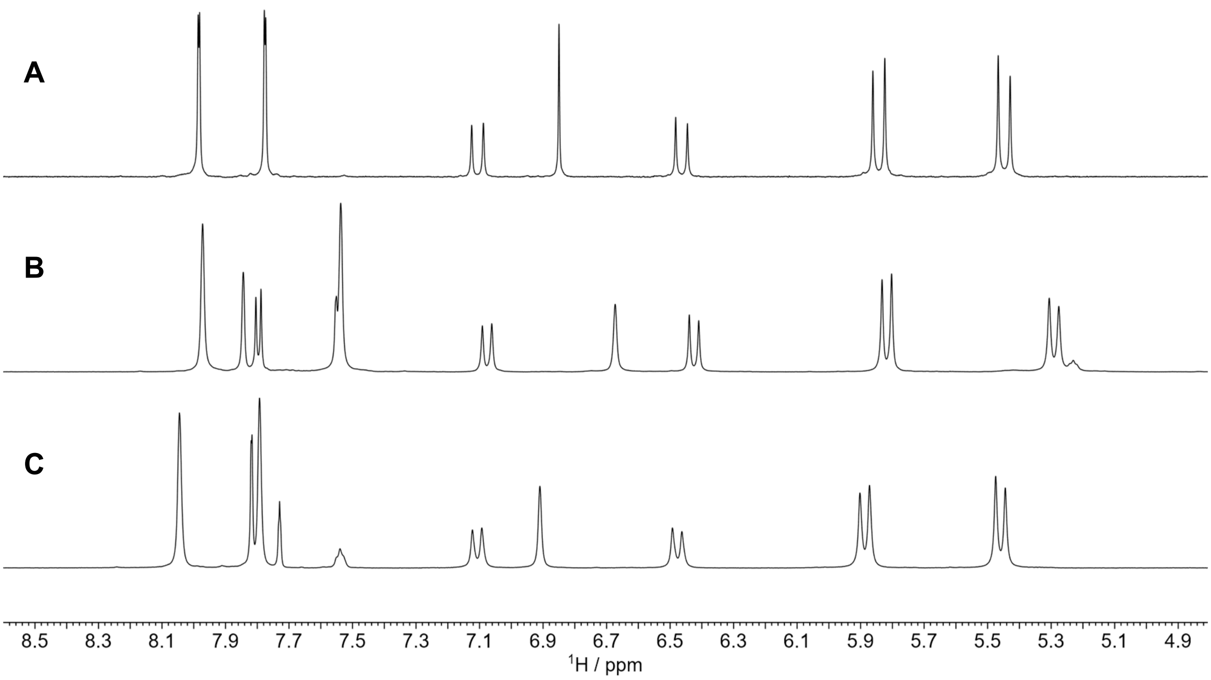

Figure S45 | Partial ^{1}H NMR spectra of **$[Ag_8L_2](PF_6)_4$** (**A**, 400.1 MHz, 295 K), **[Fmoc*-Rot]$[Ag_8L_2](PF_6)_4$** (**B**, 500.1 MHz, 298 K), and **[Amide-Rot]$[Ag_8L_2](PF_6)_4$** (**C**, 500.1 MHz, 298 K) in acetone-d_6. Substantial changes in equivalent NMR chemical shifts are only observed between the Fmoc*-based rotaxane (**B**) and the parent pillarplex (**A**).

Fmoc* Deprotection

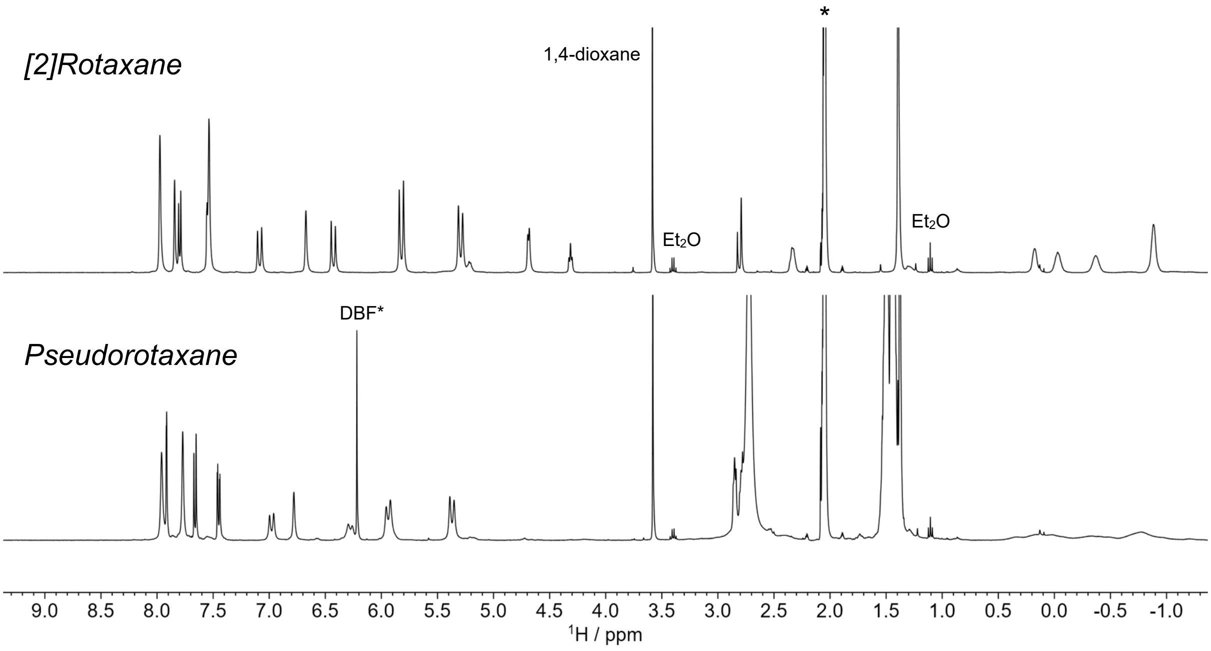

Figure S46 | Representative ^{1}H NMR spectra (500.1 MHz, 293 K) of [2]rotaxane **[Fmoc*-Rot][Ag$_8$L$_2$](PF$_6$)$_4$** before the addition of piperidine (top) and after pseudorotaxane formation *via* Fmoc* deprotection (bottom) in acetone-d_6 (* = residual solvent peak). Piperidine signals (1.2–1.6 ppm, 2.5–3.0 ppm) were vertically truncated for clarity. 1,4-Dioxane was added as an internal standard. The bottom spectrum clearly confirms pseudorotaxane formation.

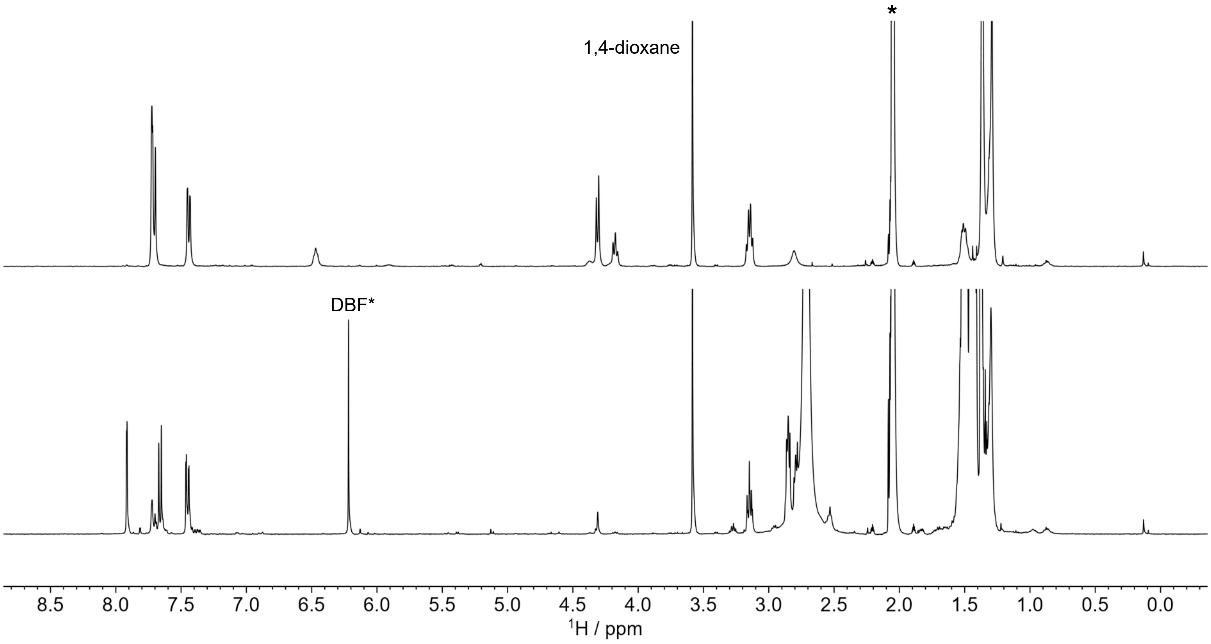

Figure S47 | Representative ^{1}H NMR spectra (500.1 MHz, 294 K) of capped axle **[Fmoc*-NH-(CH$_2$)$_6$]$_2$** before the addition of piperidine (top) and after Fmoc* deprotection (bottom) in acetone-d_6 (* = residual solvent peak). Piperidine signals (1.2–1.6 ppm, 2.5–3.0 ppm) were vertically truncated for clarity. 1,4-Dioxane was added as an internal standard.

7. HR-MS Spectra

[Amide-Rot][Ag_8L_2](PF_6)$_4$

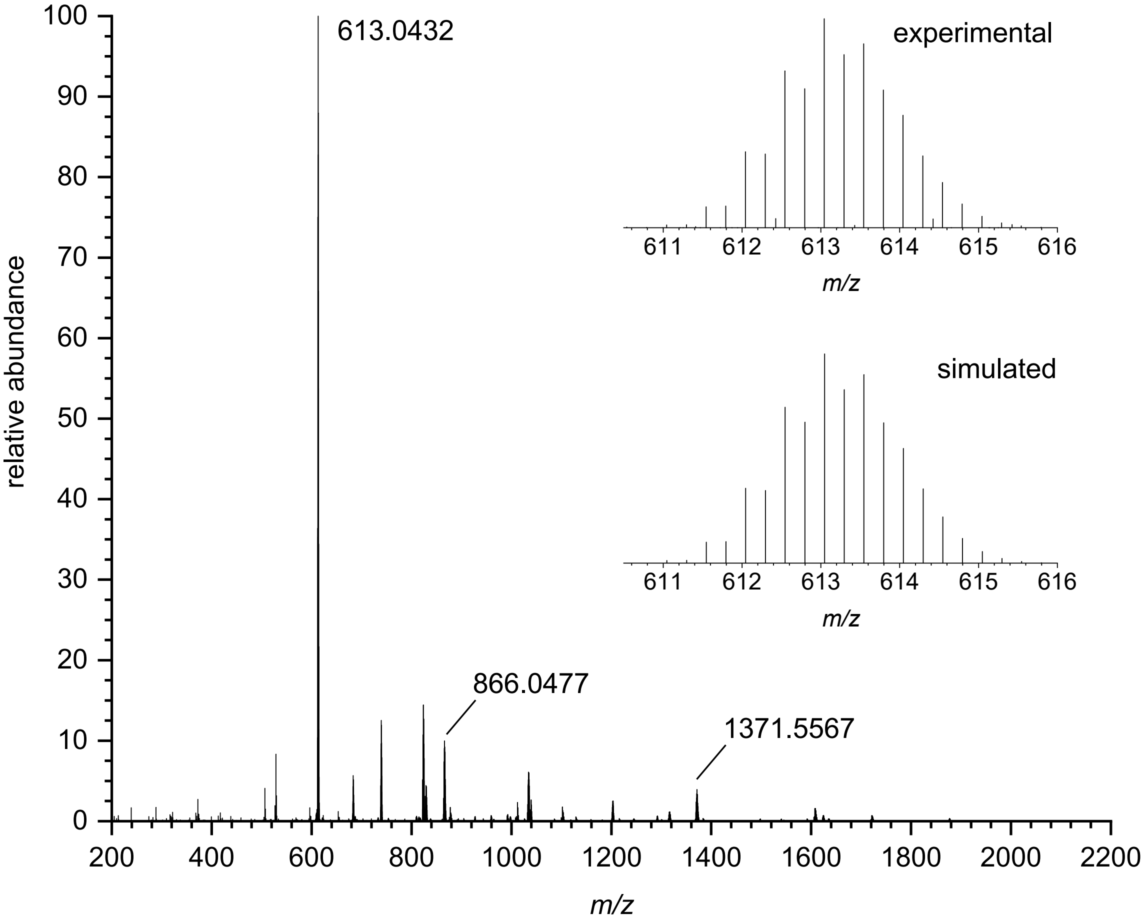

Figure S48 | HR-HESI-MS (ESI$^+$, MeCN) spectrum of **[Amide-Rot][Ag_8L_2](PF_6)$_4$** and excerpts of the **[Amide-Rot][Ag_8L_2]$^{4+}$** cation (top: experimental, bottom: simulated).

8. Crystallographic Details

Sample preparation

Single crystals of **Fmoc*[2]Rot[Ag_8L_2](PF_6)$_4$** were obtained by slow diffusion of either tetrahydropyran (solvatomorph A) or toluene (solvatomorph B) into an acetonitrile solution of the title compound (3 mg / 0.5 mL) over two weeks at ambient temperature.

Measurement

SC-XRD data were collected on a *Bruker* D8 Venture single crystal X-ray diffractometer equipped with a *Bruker* Photon CMOS detector, a TXS rotating anode with MoK_α radiation (λ = 0.71073 Å), and a Helios optic using the APEX4 software package.[12] Measurements were performed on single crystals (solvatomorph A: colorless block, solvatomorph B: colourless plate) coated with perfluorinated ether. The crystals were fixed on top of a Kapton micro sampler and frozen under a stream of cold nitrogen. A matrix scan was used to determine the initial lattice parameters. All data were integrated by SAINT using a narrow-frame algorithm and the reflections were corrected for Lorentz and polarisation effects, scan speed, and background.[13] For solvatomorph A of **Fmoc*[2]Rot[Ag_8L_2](PF_6)$_4$**, the integration of the data using a monoclinic unit cell yielded a total of 232380 reflections within a 2θ range [°] of 3.78 to 52.83 (0.80 Å), of which 30399 were independent. The integration of the data of solvatomorph B using a monoclinic unit cell yielded a total of 221248 reflections within a 2θ range [°] of 3.73 to 46.24 (0.90 Å), of which 21298 were independent. Data were corrected for absorption effects including odd and even ordered spherical harmonics by the multi-scan method SADABS.[14] Space group assignment was based upon systematic absences, E statistics, and successful refinement of the structure.

Structure Solution/Refinement

The structure was solved by direct methods using SHELXT and refined by full-matrix least-squares methods against F^2 by minimizing $\Sigma w(F_o^2–F_c^2)^2$ using SHELXL in conjunction with SHELXLE.[15-17] All non-hydrogen atoms were refined with anisotropic displacement parameters. Hydrogen atoms were refined isotropically on calculated positions using a riding model with their U_{iso} values constrained to 1.5 times the U_{eq} of their pivot atoms for terminal sp^3 carbon atoms and a C–H distance of 0.98 Å. Non-methyl hydrogen atoms were refined using a riding model with methylene, aromatic, and other C–H distances of 0.99 Å, 0.95 Å, and 1.00 Å, respectively, and U_{iso} values constrained to 1.2 times the U_{eq} of their pivot atoms.

Disorder Treatment

As is often observed for supramolecular structures, a significant number of disordered molecules was found in both solvatomorphs of **[Fmoc*-Rot][Ag_8L_2](PF_6)$_4$**, containing disordered hexafluorophosphate counter ions and Fmoc*-associated *tert*-butyl groups. All structures required disorder modelling of the (whole) alkyl axle and Fmoc* stopper groups. The disordered parts were modelled using free variables in conjunction with ISOR, SIMU, RIGU, FLAT, SADI, and SAME restraints, as implemented in the DSR plugin in SHELXLE.[18-19] Moreover, the

structures of all **[Fmoc*-Rot][Ag_8L_2](PF_6)$_4$** solvatomorphs contained disordered co-crystallized solvents (MeCN, tetrahydropyran or toluene) which could not be modelled reasonably and were treated as a diffuse contribution to the overall scattering without specific atom positions using the PLATON/SQUEEZE routine.[20]

Neutral atom scattering factors for all atoms and anomalous dispersion corrections for the non-hydrogen atoms were taken from *International Tables for Crystallography*.[21] Images of the crystal structures were generated with *PyMOL*.[22] CCDC numbers 2432100 (solvatomorph A) and 2432099 (solvatomorph B) contain the supplementary crystallographic data for different conformational isomers of **[Fmoc*-Rot][Ag_8L_2](PF_6)$_4$**. These data are provided free of charge by the joint *Access Structures* service of the *Cambridge Crystallographic Data Centre* and the *Fachinformationszentrum Karlsruhe* (www.ccdc.cam.ac.uk/structures).

Comment on data quality

For solvatomorph B of **[Fmoc*-Rot][Ag_8L_2](PF_6)$_4$**, a complete dataset was collected until 0.80 Å. However, the crystal, along with specimens from other crystallization attempts, diffracted poorly at high angles. The shells ≤ 0.90 Å were dominated by noise [I/sigma(I) < 2.0] and were therefore excluded from refinement. Additionally, electron density maxima resembling the pattern of another pillarplex fragment were observed near the metal ions of the cavitand. This suggests either whole-molecule disorder of the pillarplex or unresolved twinning. Unfortunately, no disorder model yielded satisfactory results, and no unaccounted twin domains could be found.

Table S6 | Crystal data for both solvatomorphs of **[Fmoc*-Rot][Ag_8L_2](PF_6)$_4$**.

**Compound**	**Solvatomorph A**	**Solvatomorph B**
CCDC number	2432100	2432099
Empirical formula	$C_{118}H_{147}Ag_8F_{24}N_{27}O_6P_4$	$C_{106}H_{124}Ag_8F_{24}N_{26}O_4P_4$
Formula weight	3482.46	3269.14
Temperature [K]	100(2)	100(2)
Crystal system	monoclinic	monoclinic
Space group (number)	$P\,2_1/c$ (14)	$P\,2_1/n$ (14)
a [Å]	16.845(2)	14.9398(10)
b [Å]	20.259(3)	46.220(3)
c [Å]	44.039(7)	22.6826(17)
α [°]	90	90
β [°]	95.816(3)	105.426(3)
γ [°]	90	90
Volume [Å^3]	14952(4)	15098.4(19)
Z	4	4
ρ_{calc} [g/cm^3]	1.547	1.438
μ [mm^{-1}]	1.154	1.136
F(000)	6976	6504
Crystal size [mm^3]	0.096×0.153×0.159	0.019×0.078×0.216
Crystal colour	colorless	colorless

Crystal shape	block	plate
Radiation	MoK_α (λ=0.71073 Å)	MoK_α (λ=0.71073 Å)
2θ range [°]	3.78 to 52.83 (0.80 Å)	3.73 to 46.24 (0.90 Å)
Index ranges	$-21 \leq h \leq 21$<br>$-25 \leq k \leq 25$<br>$-54 \leq l \leq 55$	$-16 \leq h \leq 16$<br>$-51 \leq k \leq 48$<br>$-24 \leq l \leq 25$
Reflections collected	232380	221248
Independent reflections	30399<br>R_{int} = 0.0537<br>R_{sigma} = 0.0302	21298<br>R_{int} = 0.1006<br>R_{sigma} = 0.0641
Completeness [%]	99.5 (to θ = 25.242°)	99.8 (to θ = 23.121°)
Data / Restraints / Parameters	30399 / 4950 / 2310	21298 / 8089 / 2094
Goodness-of-fit on F^2	1.071	1.045
Final R indexes [$I \geq 2\sigma(I)$]	R_1 = 0.0473<br>wR_2 = 0.1122	R_1 = 0.1418<br>wR_2 = 0.3716
Final R indexes [all data]	R_1 = 0.0654<br>wR_2 = 0.1239	R_1 = 0.1782<br>wR_2 = 0.4031
Largest peak/hole [eÅ^{-3}]	1.39/−0.80	2.29/−1.26

Supplementary SC-XRD Figures

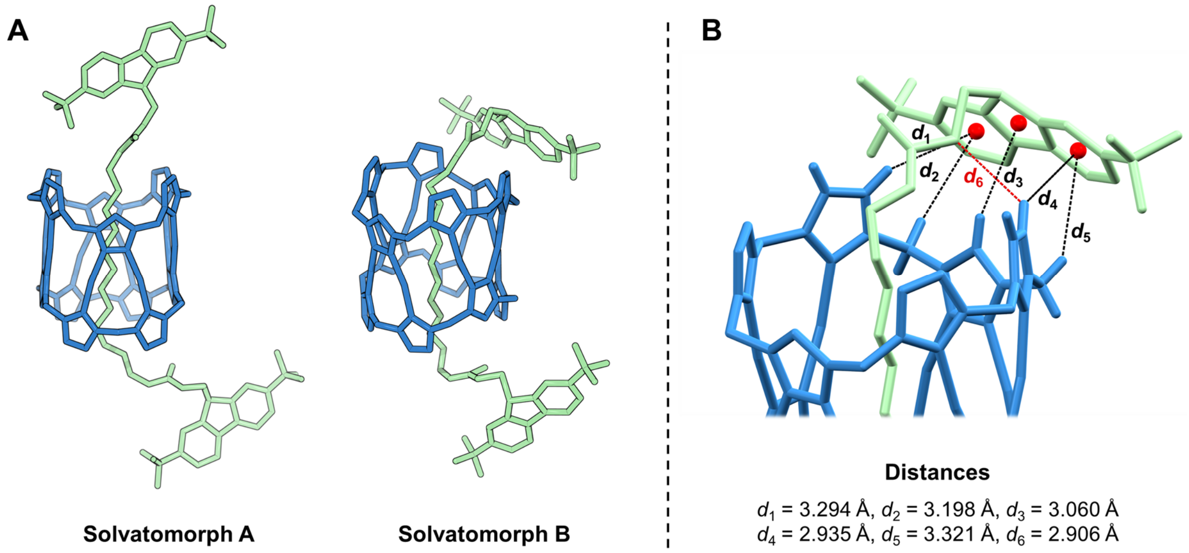

Figure S49 | (**A**) SC-XRD structure of **[Fmoc*-Rot][Ag_8L_2](PF_6)$_4$** (left: solvatomorph A; right: solvatomorph B) in capped sticks representation, showing the cationic rotaxane fragment (*color code:* capped axle = green, pillarplex ring = blue). Counterions, solvent molecules, and hydrogen atoms are omitted for clarity. In solvatomorph B, the disorder of the Fmoc* group not involved in the "wrapped" conformation with the pillarplex rim has been omitted for clarity. (**B**) Perspective view of the rotaxane fragment in solvatomorph B, highlighting selected short CH–π interactions (d_1 – d_5) and hydrogen bonding (d_6) between hydrogen atoms associated with the pillarplex rim and the aromatic system of the adjacent Fmoc* stopper. All other hydrogen atoms, counter ions and solvent molecules are omitted for clarity.

9. Computational Details

Density functional theory (DFT) calculations were performed using the *ORCA 6.0.1* quantum chemistry package.[23-24] For all calculations, tight convergence criteria were applied for self-consistent field calculations (TightSCF) and geometry optimizations (TightOPT) to ensure accuracy. To investigate interactions between the Fmoc stopper and the electron-deficient pillarplex rim, conformer analysis was performed. For computational efficiency, semi-rotaxane **[Fmoc*-Semi-Rot][Ag$_8$L$_2$]$^{4+}$**, namely the host–guest complex formed through insertion of Fmoc*-protected 1-aminododecane into the pore of **[Ag$_8$L$_2$]$^{4+}$**, was used as a model system instead of rotaxane **[Fmoc*-Rot][Ag$_8$L$_2$]$^{4+}$**. To find the energetic minimum of this assembly along with higher-energy conformers, the global geometry optimization and ensemble generator (GOAT) was employed at the GFN2-xTB level of theory.[25-26] Solvation in acetone was accounted for implicitly *via* the ddCOSMO model.[27] Conformers with energy differences up to 12 kcal/mol (50 kJ/mol) relative to the global minimum were included in the ensemble. Based on the SC-XRD structure of rotaxane **[Fmoc*-Rot][Ag$_8$L$_2$]$^{4+}$** (solvatomorph B) as a starting geometry, the algorithm identified a global minimum, as indicated by the convergence of the energy (ΔE ≤ 0.1 kcal/mol) and conformational entropy (ΔS_{conf} ≤ 0.1 cal mol^{-1} K^{-1}) at an ensemble temperature of 298.15 K (Table S7). The final ensemble comprised 43 conformers with electronic energies below 50 kJ/mol (Table S8).

Table S7 | ORCA output of the GOAT run on **[Fmoc*-Semi-Rot][Ag$_8$L$_2$]$^{4+}$** at the ddCOSMO(acetone)/GFN2-xTB level of theory.

Iteration	E_{min} / Hartree	S_{conf} / cal mol^{-1} K^{-1}	G_{conf} kcal/mol
1	-339.205736	2.610	-0.550
2	-339.205736	2.949	-0.648
3	-339.205736	3.667	-0.800
4	-339.205736	3.901	-0.844
5	-339.205736	3.986	-0.860

Table S8 | Final ensemble of the GOAT run on **[Fmoc*-Semi-Rot][Ag$_8$L$_2$]$^{4+}$** at the ddCOSMO(acetone)/GFN2-xTB level of theory.

Conformer	E / Hartree	ΔE / kJ/mol	degeneracy	% total
0	-339.205736	0	4	59.4
1	-339.205052	1.8	1	7.2
2	-339.205026	1.9	2	14.0
3	-339.204389	3.5	2	7.1
4	-339.204095	4.3	2	5.2
5	-339.203594	5.6	1	1.5
6	-339.203509	5.8	1	1.4
7	-339.203421	6.1	1	1.3
8	-339.203124	6.9	1	0.9
9	-339.202677	8.0	1	0.6
10	-339.202256	9.1	1	0.4
11	-339.201897	10.1	1	0.3
12	-339.201652	10.7	1	0.2

13	-339.201279	11.7	1	0.1
14	-339.200952	12.6	1	0.1
15	-339.200343	14.2	1	0.1
16	-339.200185	14.6	1	0.0
17	-339.199871	15.4	1	0.0
18	-339.199833	15.5	1	0.0
19	-339.199598	16.1	1	0.0
20	-339.199384	16.7	1	0.0
21	-339.199343	16.8	1	0.0
22	-339.199328	16.8	1	0.0
23	-339.199075	17.5	1	0.0
24	-339.198564	18.8	1	0.0
25	-339.198438	19.2	1	0.0
26	-339.198336	19.4	1	0.0
27	-339.197526	21.6	1	0.0
28	-339.196872	23.3	1	0.0
29	-339.196830	23.4	1	0.0
30	-339.196830	23.4	1	0.0
31	-339.196181	25.1	1	0.0
32	-339.195715	26.3	1	0.0
33	-339.195600	26.6	1	0.0
34	-339.195372	27.2	1	0.0
35	-339.195041	28.1	1	0.0
36	-339.193877	31.1	1	0.0
37	-339.193317	32.6	1	0.0
38	-339.192862	33.8	1	0.0
39	-339.190851	39.1	1	0.0
40	-339.190306	40.5	1	0.0
41	-339.189776	41.9	1	0.0
42	-339.188471	45.3	1	0.0

A representative subset of the ensemble, selected based on geometric considerations, was re-optimized at the r^{2}SCAN-3c level of theory,[28] implicitly including solvation in acetone by the conductor-like polarizable continuum model (CPCM). Eleven conformers were chosen based on differences in Fmoc orientation and proximity to the pillarplex rim to assess the impact of hydrogen bonding and CH–π interactions on their energy. Rotamers arising from C–C bond rotation (anti/gauche) of the alkyl chain were largely excluded, as their (small) energy differences compared to the selected conformers were not the focus of this analysis.
Note: In contrast, the orientation of the C–N bond involving the carbamate moiety significantly affected Fmoc* orientation, so its rotational (gauche) conformers were included.

All selected conformers were re-ranked based on their Gibbs free energies (Table S9) and frequency analysis was performed to confirm convergence to an energetic minimum. Notably, the global minimum structure (conformer 0) very closely resembles the “wrapped” motif (*cf.* Figure S49, solvatomorph B).

The thermodynamic parameters ΔG, ΔH, and $T\Delta S$ for the interconversion of the higher-energy conformers (G_i) to the global minimum (G_{min}) are summarized in Table S9 and were calculated based on the following relations:

$$\Delta G = \sum G_i - \sum G_{min}$$

$$\Delta H = \sum H_i - \sum H_{min}$$

$$T\Delta \mathrm{S} = \sum TS_i - \sum TS_{min}$$

Table S9 | Electronic energies and thermochemical quantities (Gibbs free energy G, enthalpy H, and temperature-weighted entropy TS) of selected re-optimized conformers of **[Fmoc*-Semi-Rot][Ag_8L_2]$^{4+}$** at the CPCM(acetone)/r^{2}SCAN-3c level of theory).

Conformer	Δ*E* / kJ mol^{-1}	Δ*G* / kJ mol^{-1}	Δ*H* / kJ mol^{-1}	*T*Δ*S* / kJ mol^{-1}
0	0	0	0	0
31	10.0	7.9	10.6	2.6
6	10.6	13.6	11.7	-2.0
36	11.5	14.4	9.0	-5.4
17	17.7	16.6	18.6	2.0
22	21.7	22.7	19.5	-3.2
18	30.1	26.9	31.7	4.8
35	34.5	32.4	35.5	3.0
15	33.7	35.7	32.6	-3.1
33	33.5	36.1	34.9	-1.2
32	53.7	52.0	54.8	2.8

The coordinates of all conformers found in the GOAT run, as well as the re-optimized subset are attached as xyz files. The schematic structures of the re-optimized subset are shown in Figure S50.

Re-Optimized Conformers (r^2SCAN-3c)

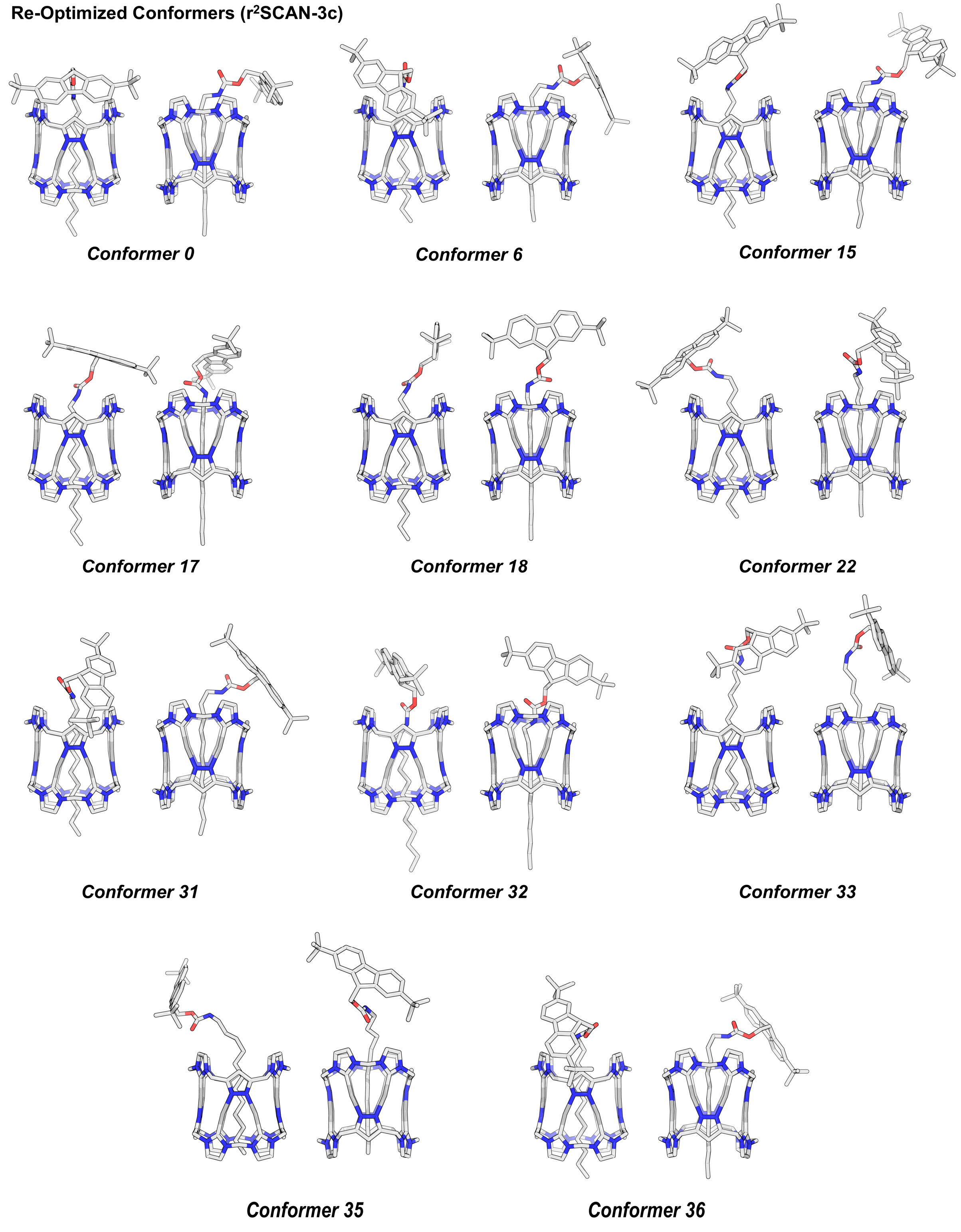

Figure S50 | Front and side views of the re-optimized subset of conformers at the r^2SCAN-3c level of theory.

10. References

[1] P. J. Altmann, C. Jandl, A. Pöthig, "Introducing a pyrazole/imidazole based hybrid cyclophane: a hydrogen bond sensor and binucleating ligand precursor" *Dalton Trans.* **2015**, *44*, 11278.

[2] P. J. Altmann, A. Pöthig, "Pillarplexes: A Metal–Organic Class of Supramolecular Hosts" *J. Am. Chem. Soc.* **2016**, *138*, 13171.

[3] K. D. Stigers, M. R. Koutroulis, D. M. Chung, J. S. Nowick, "Fmoc*: A More Soluble Analogue of the 9-Fluorenylmethoxycarbonyl Protecting Group" *The Journal of Organic Chemistry* **2000**, *65*, 3858.

[4] P. J. Altmann, A. Pöthig, "A pH-Dependent, Mechanically Interlocked Switch: Organometallic [2]Rotaxane vs. Organic [3]Rotaxane" *Angew. Chem. Int. Ed.* **2017**, *56*, 15733.

[5] M. Loos, C. Gerber, F. Corona, J. Hollender, H. Singer, "Accelerated isotope fine structure calculation using pruned transition trees" *Anal. Chem.* **2015**, *87*, 5738.

[6] T. Nakano, K. Takewaki, T. Yade, Y. Okamoto, "Dibenzofulvene, a 1,1-diphenylethylene analogue, gives a π-stacked polymer by anionic, free-radical, and cationic catalysts" *J. Am. Chem. Soc.* **2001**, *123*, 9182.

[7] R. A. M. O'Ferrall, "β-Elimination of 9-fluorenylmethanol in solutions of methanol and t-butyl alcohol" *J. Chem. Soc.* **1970**, *0*, 268.

[8] K. Ralhan, V. G. KrishnaKumar, S. Gupta, "Piperazine and DBU: a safer alternative for rapid and efficient Fmoc deprotection in solid phase peptide synthesis" *RSC Adv.* **2015**, *5*, 104417.

[9] H. Kessler, C. Griesinger, R. Kerssebaum, K. Wagner, R. R. Ernst, "Separation of cross-relaxation and J cross-peaks in 2D rotating-frame NMR spectroscopy" *J. Am. Chem. Soc.* **1987**, *109*, 607.

[10] J. Schleucher, J. Quant, S. J. Glaser, C. Griesinger, "A theorem relating cross-relaxation and Hartmann-Hahn transfer in multiple-pulse sequences. Optimal suppression of TOCSY transfer in ROESY" *J. Magn. Reson. A* **1995**, *112*, 144.

[11] E. Ämmälahti, M. Bardet, D. Molko, J. Cadet, "Evaluation of distances from ROESY experiments with the intensity-ratio method" *J. Magn. Reson. A* **1996**, *122*, 230.

[12] Bruker, APEX 4 (2022.10-0), Madison, Wisconsin (USA), **2022**.

[13] Bruker, SAINT (8.40A), Madison, Wisconsin (USA), **2019**.

[14] Bruker, SADABS (2016/2), Madison, Wisconsin (USA), **2016**.

[15] C. B. Hübschle, G. M. Sheldrick, B. Dittrich, "ShelXle: a Qt graphical user interface for SHELXL" *J. Appl. Crystallogr.* **2011**, *44*, 1281.

[16] G. M. Sheldrick, "SHELXT - integrated space-group and crystal-structure determination" *Acta Crystallogr., Sect. A: Found. Crystallogr.* **2015**, *71*, 3.

[17] G. M. Sheldrick, "Crystal structure refinement with SHELXL" *Acta Crystallogr., Sect. C: Cryst. Struct. Commun.* **2015**, *71*, 3.

[18] D. Kratzert, J. J. Holstein, I. Krossing, "DSR: enhanced modelling and refinement of disordered structures with SHELXL" *J. Appl. Crystallogr.* **2015**, *48*, 933.

[19] D. Kratzert, I. Krossing, "Recent improvements in DSR" *J. Appl. Crystallogr.* **2018**, *51*, 928.

[20] A. Spek, "PLATON SQUEEZE: a tool for the calculation of the disordered solvent contribution to the calculated structure factors" *Acta Crystallogr., Sect. C: Cryst. Struct. Commun.* **2015**, *71*, 9.

[21] *International Tables for Crystallography, Volume C: Mathematical, physical and chemical tables*, International Union of Crystallography, Chester, England, **2006**.

[22] The PyMOL Molecular Graphics System (Version 2.5.2), Schrödinger, LLC.

[23] F. Neese, "The ORCA program system" *Wiley Interdiscip. Rev.: Comput. Mol. Sci.* **2012**, *2*, 73.

[24] F. Neese, "Software update: The ORCA program system—Version 5.0" *Wiley Interdiscip. Rev.: Comput. Mol. Sci.* **2022**, *12*, e1606.

[25] C. Bannwarth, S. Ehlert, S. Grimme, "GFN2-xTB-an accurate and broadly parametrized self-consistent tight-binding quantum chemical method with multipole electrostatics and density-dependent dispersion contributions" *J. Chem. Theory Comput.* **2019**, *15*, 1652.

[26] C. Bannwarth, E. Caldeweyher, S. Ehlert, A. Hansen, P. Pracht, J. Seibert, S. Spicher, S. Grimme, "Extended tight-binding quantum chemistry methods" *Wiley Interdiscip. Rev.: Comput. Mol. Sci.* **2021**, *11*, e1493.

[27] M. Stahn, S. Ehlert, S. Grimme, "Extended conductor-like polarizable continuum solvation model (CPCM-X) for semiempirical methods" *J. Phys. Chem. A* **2023**, *127*, 7036.

[28] S. Grimme, A. Hansen, S. Ehlert, J.-M. Mewes, "r2SCAN-3c: A "Swiss army knife" composite electronic-structure method" *J. Chem. Phys.* **2021**, *154*, 064103.
